# Discovery of Nanomolar-Affinity Pharmacological Chaperones Stabilizing the Oncogenic p53 Mutant Y220C

**DOI:** 10.1021/acsptsci.2c00164

**Published:** 2022-10-11

**Authors:** Joseph R. Stephenson Clarke, Leon R. Douglas, Patrick J. Duriez, Dimitrios-Ilias Balourdas, Andreas C. Joerger, Raniya Khadiullina, Emil Bulatov, Matthias G. J. Baud

**Affiliations:** ^†^School of Chemistry and ^⊗^Institute for Life Sciences, University of Southampton, Southampton SO17 1BJ, United Kingdom; ‡ Cancer Research UK, Somers Building, University Hospital Southampton, Tremona Road, Southampton SO16 6YD, United Kingdom; § Centre for Cancer Immunology, University Hospital Southampton, Coxford Road, Southampton SO16 6YD, United Kingdom; ⊥ Institute of Pharmaceutical Chemistry, Johann Wolfgang Goethe University, Max-von-Laue-Straße 9, 60438 Frankfurt am Main, Germany; ∥ Buchmann Institute for Molecular Life Sciences and Structural Genomics Consortium, Max-von-Laue-Straße 15, 60438 Frankfurt am Main, Germany; # Institute of Fundamental Medicine and Biology, Kazan Federal University, Kazan 420008, Russia

**Keywords:** mutant p53, small molecule, pharmacological chaperoning, structure-based drug design, anticancer therapy

## Abstract

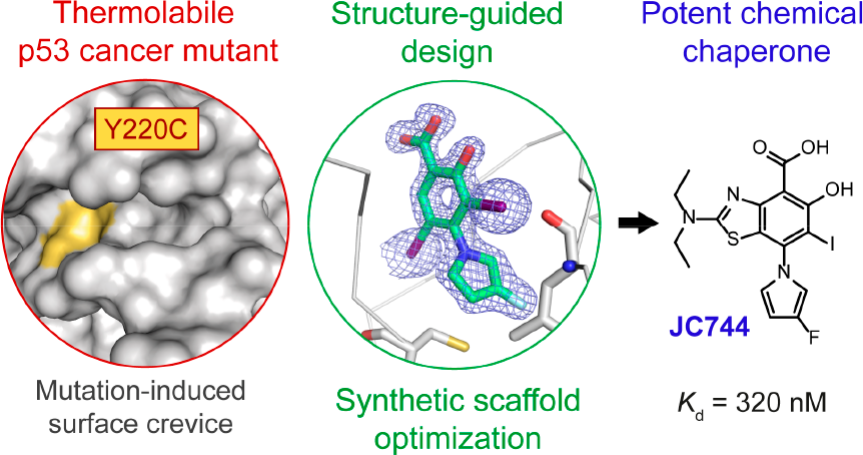

The tumor suppressor protein p53 is inactivated in the majority of human cancers and remains a prime target for developing new drugs to reactivate its tumor suppressing activity for anticancer therapies. The oncogenic p53 mutant Y220C accounts for approximately 125,000 new cancer cases per annum and is one of the most prevalent p53 mutants overall. It harbors a narrow, mutationally induced pocket at the surface of the DNA-binding domain that destabilizes p53, leading to its rapid denaturation and aggregation. Here, we present the structure-guided development of high-affinity small molecules stabilizing p53-Y220C *in vitro*, along with the synthetic routes developed in the process, *in vitro* structure–activity relationship data, and confirmation of their binding mode by protein X-ray crystallography. We disclose two new chemical probes displaying sub-micromolar binding affinity *in vitro*, marking an important milestone since the discovery of the first small-molecule ligand of Y220C in 2008. New chemical probe JC744 displayed a *K*_d_ = 320 nM, along with potent *in vitro* protein stabilization. This study, therefore, represents a significant advance toward high-affinity Y220C ligands for clinical evaluation.

## Introduction

The tumor suppressor p53, also referred to as the “guardian of the genome”, is a tetrameric transcription factor central to the human anticancer response through its regulation of critical gene networks controlling apoptosis, senescence, and DNA repair.^1−4^ Impaired p53 signaling is a hallmark of the estimated 20 million new cancer cases reported yearly worldwide,^5^ and the p53 protein is directly inactivated by mutation in approximately 50% of these, with the majority of point mutations arising in its DNA-binding domain (DBD, aa 94–292).^6−10^ Its crucial role in tumor suppression makes p53 a high-profile target in oncology. Not only does expression of mutant p53 lead to impaired signaling, but p53 mutants can also exert a dominant-negative effect on remaining copies of the wild-type (WT) protein by heterotetramerization or co-aggregation, and also inactivate the paralogous tumor suppressors p63 and p73 by co-aggregation.^11−13^ Many cancers acquire further proliferative advantages by retaining only the mutated p53 allele, exhibiting an oncogenic gain-of-function that is associated with increased cell growth, drug resistance, and metastatic potential.^13−16^

Most p53 single-nucleotide variants are mis-sense mutations and can be classified into “contact” mutations that cause the obstruction or loss of essential DNA-binding contacts and “structural” mutations that inactivate the protein through mutational reduction of thermostability.^4,9−11^ An estimated one-third of common p53 cancer mutants are temperature-sensitive structural mutants and undergo loss of function through rapid denaturation under physiological conditions.^17,18^ Remarkably, some unstable structural mutants display WT-like conformation and residual transcriptional activity at sub-physiological temperatures, leading to the hypothesis that they may be reactivated through modulation of thermal stability.^19−21^ Surface-exposed tyrosine Y220 is the seventh most frequently mutated p53 residue in human cancers, accounting for ca. 1.6% of all mutations and 143,000 new cancer cases per annum worldwide, with the cysteine variant Y220C alone accounting for 125,000 of those cases.^11,22,23^ The Y220C mutation destabilizes the DBD by ca. 4 kcal/mol and reduces its melting temperature from 45 °C (WT) by approximately 8–9 °C, leading to rapid unfolding at body temperature.^7,18,24^ In addition, this large-to-small residue mutation creates a well-defined, narrow surface pocket in the p53 DBD.^25^ This pocket is conveniently located distant from the interfaces involved in DNA-binding and protein–protein interactions, presenting an opportunity for intervention using small molecules stabilizing the DBD functional fold.

Molecular chaperoning is a technique whereby the correct folding of primary protein sequences into their active conformation is assisted using stabilizing partners such as heat-shock proteins (Hsp).^26^ Hsp-mediated stabilization is a highly effective strategy employed by nature and has demonstrated efficacy toward stabilization of thermolabile p53 mutants.^27,28^ The effectiveness of this strategy for protein reactivation reveals an opportunity to achieve a similar effect using synthetic additives (pharmacological chaperoning). Despite recent successes in the development of small-molecule drug candidates that operate through p53 reactivation, many suffer key drawbacks like high toxicity (Nutlins, COTI-2, arsenic trioxide), nonspecific oxidative damage (APR-246, ZMC1, PK11007), or PAIN motifs (ZMC1, COTI-2).^29−41^ The so-called “Holy Grail” of p53 drug discovery, a nontoxic cancer-selective reactivator of transcription, thus remains a prime target, and research into Y220C stabilizers paves the way toward generic chaperones that may be applicable to the 2–3 million annual cancer cases related to unstable mutant p53.^10^

To date, several small-molecule drug discovery campaigns have targeted p53-Y220C, culminating in the development of a small number of chemical probes that selectively bind and stabilize the mutant DBD.^24,42^ Carbazole PK9328 (**1**) binds to the Y220C pocket with high affinity (*K*_d_ = 2 μM) and induces a thermal shift (Δ*T*_m_) to the protein melting temperature of over 3 °C (250 μM [ligand]).^24^**1** also reduces p53 aggregation *in vitro* and induces selective cell viability reduction of cancer cell lines HUH-7 (p53-Y220C) and NUGC-3 (p53-Y220C) versus controls HUH7-F1 (engineered p53-Y220C KO) and NUGC-4 (p53-WT), although only within a narrow concentration range.^24^ We recently reported the early optimization of iodophenol fragments, which led to the discovery of aminobenzothiazole derivatives **2** and **3**.^42^ Leads MB710 (**2**) and MB725 (**3**) stabilize p53-Y220C *in vitro* in a concentration-dependent manner (DSF) and bind potently to the Y220C pocket (*K*_d_ = 4 μM, ITC). Crucially, cell-permeable analogue **3** induces selective cell viability reduction of p53-Y220C cancer cell lines HUH-7 (liver), BXPC-3 (pancreas), and NUGC-3 (stomach), while maintaining comparatively low toxicity in the same concentration range in representative cancer lines NUGC-4 (stomach, p53 WT), SW1088 (brain, p53-R273C), WI38 (human fibroblasts, p53-WT), along with in house CRISPR-engineered HUH-7 p53-Y220C KO cells. It further reduces NUGC-3 viability (<10% at 10 μM MB725 (**3**)), correlating with enhanced and selective transcription of p53 target genes *PUMA*, 9*p21*, *BTG2*, *FAS*, *TNF*, and *TNFRSF10B*, which promote apoptosis or cell cycle arrest, suggesting ligand-mediated transcriptional activation of p53-Y220C (qPCR study of 84 genes under p53 control).^42^ The correlation between *in vitro* thermal shift, selective viability reduction and upregulation of p53 signaling in Y220C cell lines represents an important milestone toward first-in-class anticancer drugs that rescue p53-Y220C function.^24,42^ However, it has also become clear that more potent ligands/stabilizers with enhanced properties will be required to develop a viable treatment in the future.

**Figure 1:**
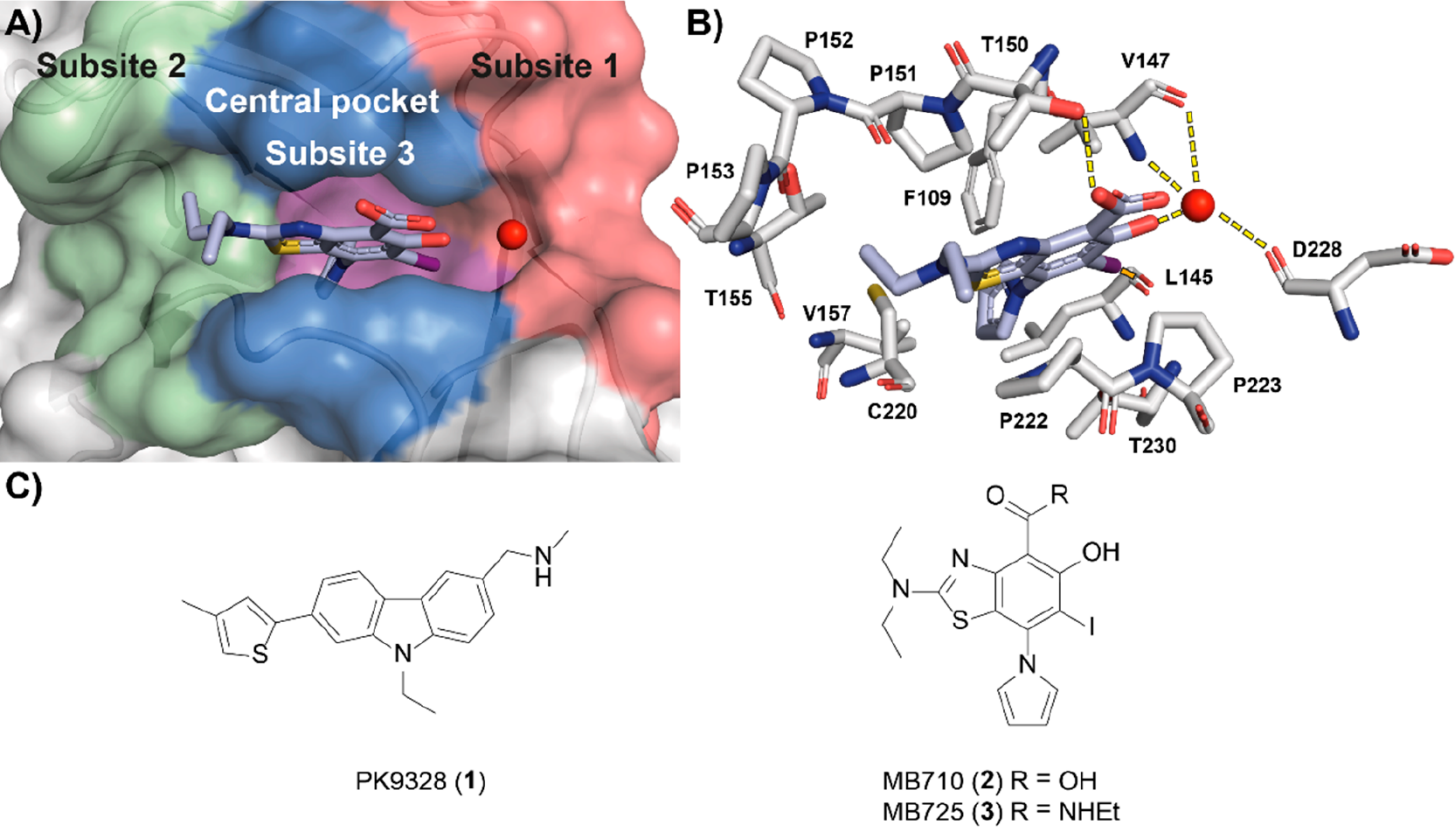
(A) Crystal structure of **2** (blue sticks) in complex with p53-Y220C (PDB: 5O1I, surface representation)^42^ with subsites referred to throughout the text color-coded: subsite 1 = red, subsite 2 = green, buried subsite 3 = purple, central pocket = blue. A structural water molecule interacting with **2** is shown as a red sphere. (B) Zoom-in crystal structure of **2** (blue sticks) in complex with p53-Y220C with interacting residues shown (PDB: 5O1I, gray sticks).^42^ The halogen bond to L145 (dashed orange line) and the hydrogen bond network between the salicylate moiety and the protein are shown (dashed yellow lines). (C) Chemical structures of representative iodophenol and carbazole lead molecules **1**–**3**.

The Y220C pocket is divided into three subsites that interact with different parts of the ligand (Figure 1). The benzothiazole core of **2** occupies the central region of the pocket and engages in extensive hydrophobic and CH−π contacts with surrounding residues V147, P151, P222, and P223. The carboxylic acid is solvent-exposed and hydrogen bonds to T150 while conferring aqueous solubility to the molecule. The hydroxyl forms an H-bond to a conserved structural water molecule tri-coordinated by V147 and D228 in subsite 1, and the iodine engages in hydrophobic interactions with L145 and V147, and a key halogen bond with the L145 carbonyl. Oxygen–halogen bonding is highly directional and is critical to binding of this series; modification to lighter halogens reduces binding affinity approximately 20-fold (I→Cl).^43^ The diethylamino group extends to access subsite 2 and forms contacts with a hydrophobic hotspot, formed by P151, P152, P153, T155, and P222. The pyrrole is buried at the bottom of the pocket in subsite 3, engaging in extensive hydrophobic interactions with F109, L145, V147, P151, V157, C220, and L257 side chains in addition to CH−π interactions with polarized C220 β-hydrogens. Incorporation of a pyrrole at this position was shown to boost affinity by ca. 40-fold compared with an unsubstituted iodophenol. Comparatively, H-bonding of the phenol and carboxylate modulate *in vitro* binding affinity by less than 10-fold.^42,44^

Affinity optimization on aminobenzothiazole and carbazole scaffolds to date have focused on subsite 2, while subsite 1 modifications moderately improved affinity (ca. 10-fold).^24,42,43^ Conversely, subsite 3 modification has remained relatively untapped beyond initial incorporation of the pyrrole, presenting an opportunity for further optimization toward higher-affinity leads. Here, we report the structure-based design, chemical synthesis, and biophysical and structural characterization of libraries of MB710/725 analogues carrying diverse substitution patterns targeting subsite 3. Critically, this involved developing new synthetic routes that circumvent the limited scalability of routes we previously reported. In-depth *in vitro* structure–activity relationship (SAR) studies identified two fluorinated derivatives displaying nanomolar binding affinity *in vitro*, with protein–fluorine contacts unambiguously characterized by protein X-ray crystallography.

## Results and Discussion

### Modeling and Library Design

**Figure 2:**
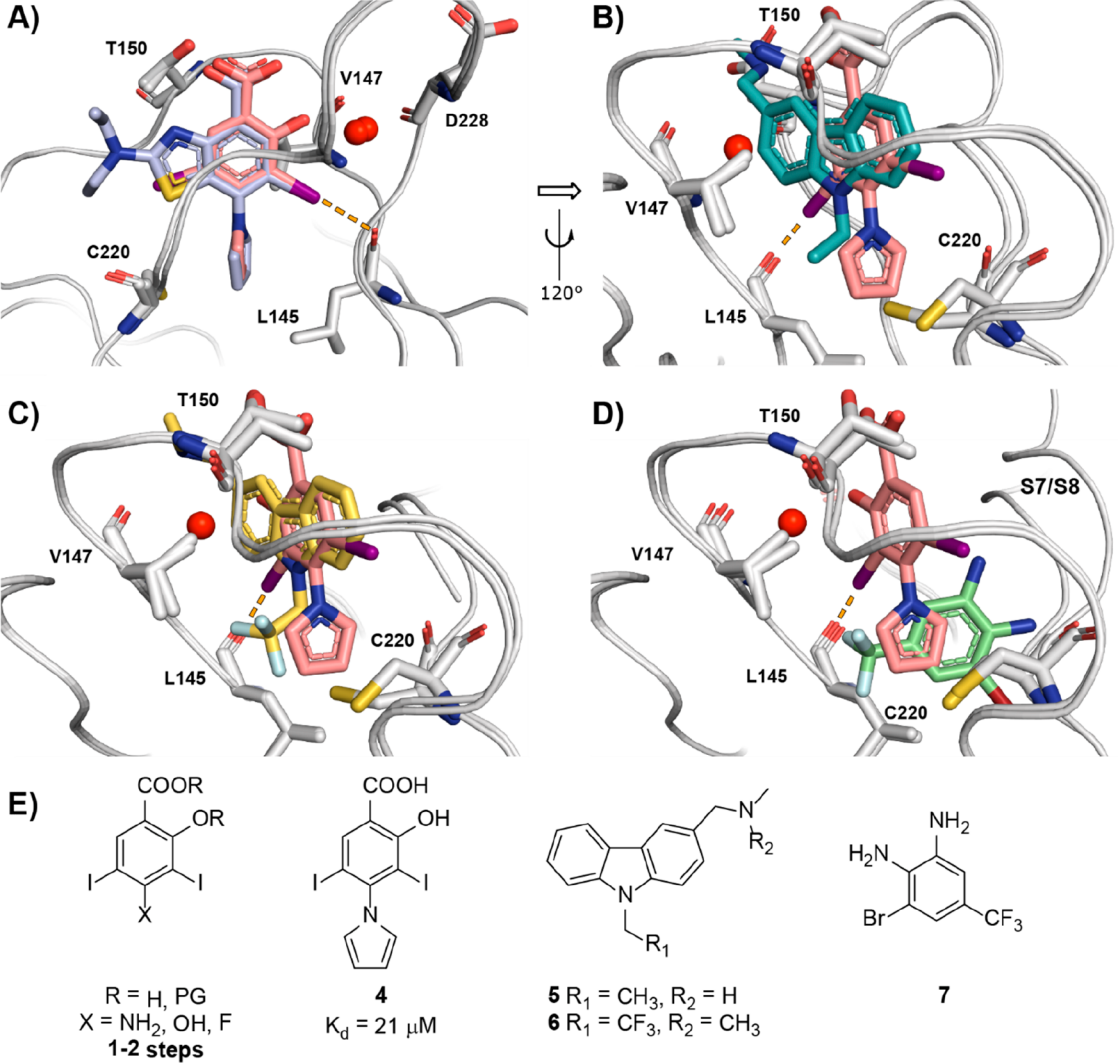
(A) Overlay of bound **2** and **4**. (B) Overlay of bound **4** and **5**. (C) Overlay of bound **4** and **6**. (D) Overlay of bound **4** and **7**. (E) Structures of iodophenol precursors and Y220C fragment ligands **4**–**7**. Color coding: **2**, light blue (PDB: 5O1I);^42^**4**, pink sticks (PDB: 5AOJ);^44^**5**, gray sticks (PDB: 2VUK);^51^**6** yellow sticks (PDB: 5G4O);^45^**7**, green sticks (PDB: 5AOL).^44^ The halogen bond to L145 is shown as dashed orange lines.

We selected the diiodosalicylic acid derivative **4** for initial library design and SAR development (Figure 2A). Diiodination of commercial and cheap salicylate precursors provided high-yielding and scalable access to diiodosalicylate precursors with diverse functionalizable handles at the 4-position (*vide infra*) (Figure 2E). Furthermore, we have previously reported high-resolution (1.4 Å) X-ray crystal structures of **2** and **4** bound to Y220C and shown that they display virtually identical binding modes and positioning of the pyrrole unit in the pocket (Figure 2A). Hence, we envisaged that SAR and fruitful modifications emerging from derivatization of the pyrrole unit in **4** would be transposable onto more potent benzothiazole analogues to yield optimized ligands.

Alignment of bound structures of **4** with representative carbazoles **5** and **6** highlighted short-chain aliphatic groups that could be incorporated into the iodophenol scaffold by pyrrole substitution or linked by a tractable *N-* or *O-* handle (Figure 2B,C). We reasoned that introducing diverse methylation patterns on the pyrrole ring to perform a “methyl scan” would allow probing for new hydrophobic hotspots within subsite 3. We previously reported on the structural dynamics of the Y220C binding pocket using X-ray crystallography and molecular dynamics simulations that highlighted the flexibility of the binding pocket in the subsite 3 region in the immediate environment of C220. This suggested that, while seemingly narrow in most bound crystal structures, subsite 3 may display some degree of flexibility in solution and be able to accommodate short hydrophobic groups protruding from main lead scaffolds.^44^ This is further supported by the partial opening/rearrangement of the pocket, involving the S7/S8 loop, upon binding of certain fragments, including trifluoromethylated dianiline **7**. Alignment of bound crystal structures of **4** with dianiline **7** suggested merging the pyrrole with the benzene ring of **7** toward a 1-arylindole, potentially conferring significant growth into subsite 3 (Figure 2D). Evaluation of such analogues may shed light on whether flexibility of the S7/S8 loop is tolerated with our ligand series, or whether its movement perturbs binding of the iodophenol scaffold. Alignment of **4** with **6** and **7** (Figure 2C,D) further identified fluorinated motifs that may be accommodated in subsite 3 such as fluoro-/trifluoromethyl-pyrroles and fluoroalkyl groups. Introduction of fluorine to subsite 3 groups has been shown to enhance the binding affinity of non-fluorinated parent **5** by up to 4-fold by invoking multipolar interactions with receptor carbonyls and the C220 sulfhydryl group.^45^ Manipulation of fluorination patterns also has the potential to allow for modulation of key physical properties (LogD/P, solubility, metabolic stability).^46^ Modification of pyrrole electronics is of particular interest as the heterocycle is electron-rich (aromaticity 61–68% of PhH) and thought to be readily oxidized by cytochrome P450 enzymes.^47−49^ Difluorination of 1*H*-pyrrole was estimated to reduce its p*K*_a_ by ca. 4.7 units (50,000-fold, in THF), suggesting that incorporation of electron-withdrawing substituents is highly effective for modulating pyrrole ring electronics and may confer improved metabolic stability to our ligands.^50^

The rational design was further supported by Glide (Schrödinger) docking studies.^52^ All derivatives performed well during *in silico* studies, attaining consistent docking scores in a similar range as the positive controls **2** and **4**. The best-scoring analogues were selected for synthesis and SAR evaluation. Conservation of key interactions (halogen/H-bonds) and binding mode were used as additional informant to prioritize compounds selected for synthesis (examples in Figure S1).

### Synthetic Chemistry

**Scheme 1:**
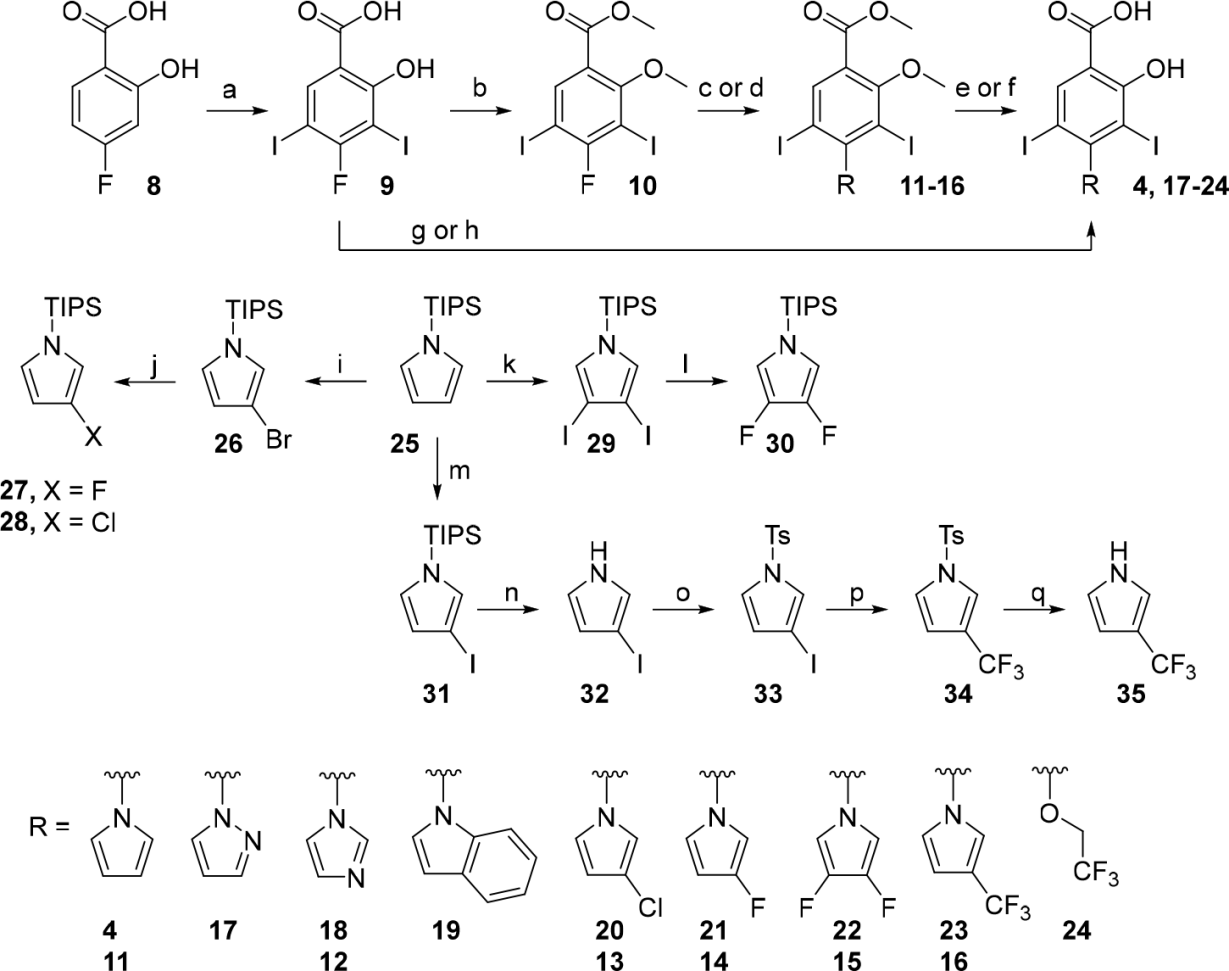
S_N_Ar Chemistry toward Subsite 3 Iodophenol Analogues Conditions: (a) NIS, AcOH, 25 °C, 6 h, 86%; (b) K_2_CO_3_, Me_2_SO_4_, NMP, 80 °C, 1 h, 68%; (c) heterocycle, Cs_2_CO_3_, DMSO, 70 °C, 1 h, 45–68%; (d) KF, 1-TIPS-pyrrole derivative, Cs_2_CO_3_, DMSO, 70 °C, 1 h, 73–80%; (e) BBr_3_, CH_2_Cl_2_, 0 to 25 °C, 24 h, 27–62%; (f) TMSI, CH_2_Cl_2_, 50 °C, sealed tube, 24 h, 31%; (g) Cs_2_CO_3_, heterocycle, DMSO, 150 °C, 2 h, 55–58%; (h) CF_3_CH_2_OH, NaH, DMF, 0 to 150 °C, 20 h, 22%; (i) NBS, THF, −78 to 25 °C, 3 h, 89%; (j) *n*BuLi, NFSI or NCS, THF, −78 to 25 °C, 1.5 h, 48–50%; (k) I_2_, H_5_IO_6_, Et_2_O, 25 °C, 1 h, 86%; (l) *n*BuLi, NFSI, THF, −78 to 25 °C, then *n*BuLi, NFSI, THF, −78 to 25 °C, 1 h, 32%; (m) NIS, acetone, −78 to 25 °C, 5 h; (n) TBAF, THF, 25 °C, 1 h; (o) NaH, TsCl, THF, 0 to 25 °C, 1 h, 84% over 3 steps; (p) FSO_2_CF_2_COOCH_3_, CuI, HMPA, DMF, 80 °C, 16 h, 62%; (q) Mg, MeOH, 25 °C, 0.5 h, 73%.

The synthesis of *N*-heterocyclic subsite 3 analogues hinged on manipulation of commercial *N*-TIPS-pyrrole and coupling to an activated 4-fluorosalicylic acid-derived partner via nucleophilic aromatic substitution (S_N_Ar, Scheme 1). Transition-metal-based Ullman and Buchwald protocols were incompatible with the reducible 3,5-diiodo motif, and iodination had to precede introduction of the electron-rich 5-membered heterocycles. Therefore, an S_N_Ar strategy was devised using 4-fluorobenzoic acids **9** and **10**. This convergent synthetic route provided access to a range of new derivatives functionalized with substituted pyrrole and other *N*-heterocyclic units not accessible by the dicarbonyl condensation chemistry we previously reported.^42^

3-Fluoropyrrole **27** was prepared as reported, via bromination of *N*-TIPS-pyrrole **25** followed by halogen–lithium exchange and quenching the aryllithium with NFSI.^53^ 3-Chloropyrrole **28** was accessed similarly by reacting the lithiopyrrole intermediate with NCS.^54^ 3,4-Difluoropyrrole **30** was prepared using similar chemistry but utilizing 3,4-diiodopyrrole **29**, which can be formed in higher yield than the dibromo equivalent and was expected to lithiate more efficiently.^55,56^ A new, scalable and regioselective route to 3-trifluoromethylpyrrole was devised by sterically controlled mono-iodination of **25** at the 3-position, protecting group exchange and gram-scale trifluoromethylation using methyl fluorosulfonyl-2,2-difluoroacetate. Subsequent mild desulfonation using magnesium in methanol afforded 3-(trifluoromethyl)-1*H*-pyrrole **35** in 98% yield as a ∼1:1 mixture (NMR) with Et_2_O; however, due to its volatility (bp ≈ 168–170 °C), the isolated (dry) yield was reduced to 73%.^57^ This preparation afforded iodinated intermediate **33** in 84% yield over 3 steps and 3-(trifluoromethyl)-1*H*-pyrrole **35** in 51% contained yield (38% isolated) over 5 steps. Importantly, this route circumvented the requirement for high temperatures (>200 °C), long reaction times (>5 days), and particularly 2- vs 3- selectivity issues encountered in past syntheses of **35** because separation of the isomers is challenging.^57−60^

4-Fluorosalicylic acid **8** was readily iodinated to coupling partner **9** in 86% yield. S_N_Ar reactions using **9** proceeded in 55–58% yields to afford salicylate derivatives in 2 steps, precluding the use of protecting groups. For heterocycles that were unstable under these conditions (150 °C), a protection strategy was employed that permitted the use of milder conditions. **9** was dimethylated using dimethyl sulfate, and subsequent S_N_Ar proceeded in moderate yields at 70 °C (45–68%).

One-pot deprotection of *N*-TIPS-pyrroles **27**, **28**, and **30** and S_N_Ar by treatment with KF/Cs_2_CO_3_ and the aryl fluoride **10** afforded methoxy-protected analogues **13**–**16** in 73–80% yields (Scheme 1). Interestingly, either omission of Cs_2_CO_3_ from the reaction conditions or replacement of KF/Cs_2_CO_3_ with CsF led to drastic reduction in conversion to the *N*-arylpyrrole. Cs^+^ has been proposed to accelerate nucleophilic substitution reactions by exergonic formation of cesium halide salts, rationalizing this observation.^61^ Demethylation using BBr_3_ or TMSI furnished deprotected salicylates **4** and **17**–**23**. 2,2,2-Trifluoroethoxy analogue **24** was prepared using a modified S_N_Ar procedure because the chemistries used for the oxyether library were unsuccessful (*vide infra*).

**Scheme 2:**
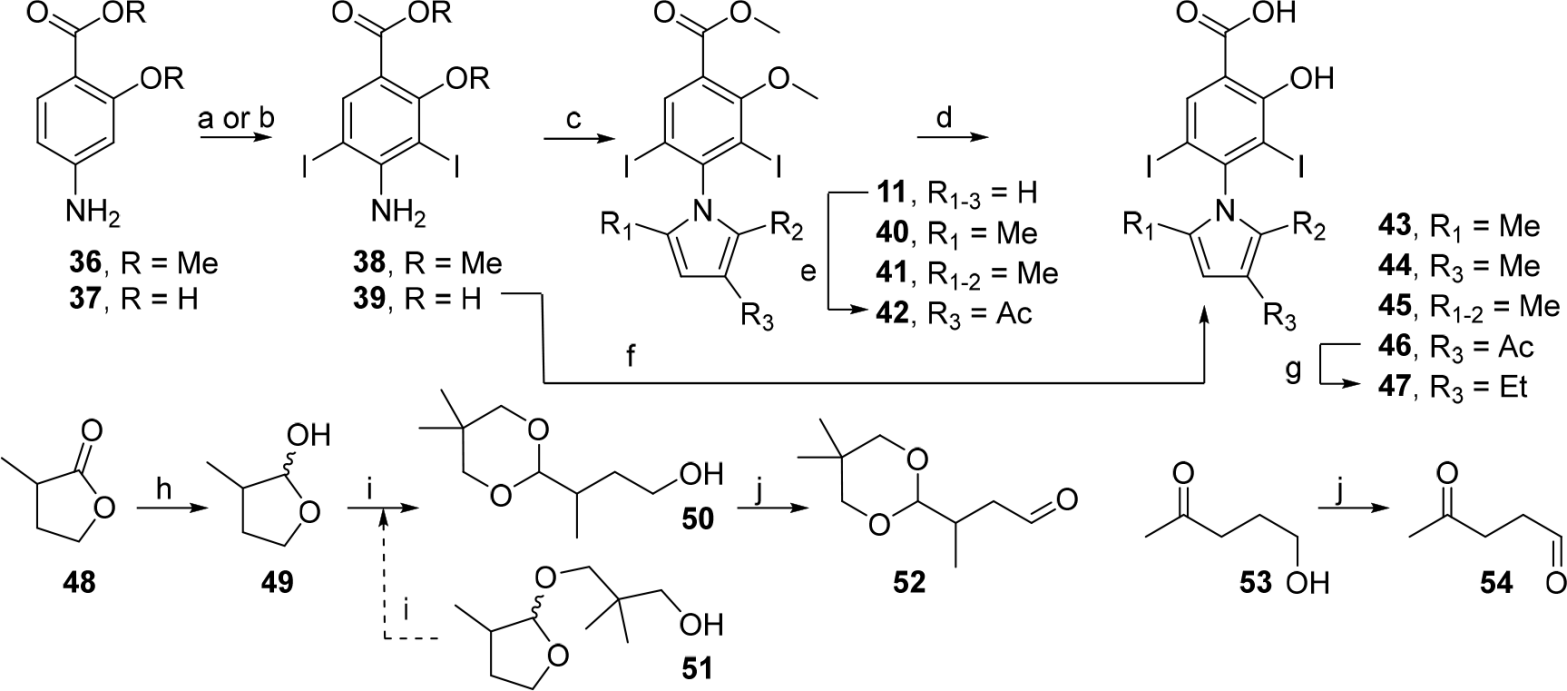
Carbonyl Condensation Chemistry toward Subsite 3 Iodophenol Analogues R_1–3_ = H unless defined. Conditions: (a) NIS, AcOH, 25 °C, 1 h, 98%; (b) NIS, MeCN, 25 °C, 0.5 h, quant.; (c) dicarbonyl, conc. aq. HCl (cat.), EtOH, reflux, 18 h, 22–35%; (d) BBr_3_, CH_2_Cl_2_, 0 to 25 °C, 24 h, 27–59%; (e) Ac_2_O, BF_3_·OEt_2_, CH_2_Cl_2_, 0 to 25 °C, 2 h, 57%; (f) **52**, AcOH, 100 °C, 6 h, 26%; (g) Et_3_SiH, TFA, 50 °C, 1 h, 48%; (h) DIBAL-H, Et_2_O, −78 °C, 30 min, 81%; (i) neopentyl glycol, TsOH·H_2_O, PhMe, 100 °C, 2 h, 46–50% **50**, 19–28% **51**; (j) SO_3_·py, Et_3_N, DMSO, CH_2_Cl_2_, 0 to 25 °C, 15 h, 69%–quant.

A range of alkylpyrrole analogues were accessible through Paal–Knorr chemistry with commercial and synthesized 1,4-dicarbonyl compounds (Scheme 2). α-Methyl-γ-butyrolactone **48** was reduced in high yield to the lactol **49**, which was ring-opened under acetalization conditions to give **50** in 46% yield. Conveniently, the byproduct **51** could be recycled to yield a second crop of **50** by exposure to the same reaction conditions. Final Parikh–Doering oxidation of **50** and commercially available γ-hydroxyketone **53** afforded the condensation partners **52** and **54**, respectively.

Aniline precursors **38** and **39** were prepared by iodination of commercial salicylic acids **36** and **37**. Paal–Knorr condensation of 1,4-dicarbonyl precursors with **38** or **39**, followed by demethylation using BBr_3_, afforded methylated pyrrole derivatives **43**–**45** (Scheme 2). Although **39** was reactive to masked dialdehyde **52**, ketones did not react under the same conditions and required protected analogue **38**. The reason behind this observation is unclear, although it is possible that **38** benefits from greater amine nucleophilicity due to reduced conjugation of the ester with the aniline ring as a result of sterics (OMe–MeO). Exploiting the bulky iodine atoms for steric deactivation of the pyrrole 2-position, acetylation of **11** produced the electronically disfavored 3-acetylpyrrole **42** in good yield. Subsequent methoxy deprotection and reduction with triethylsilane afforded the 3-ethylpyrrole **47**.

**Scheme 3:**
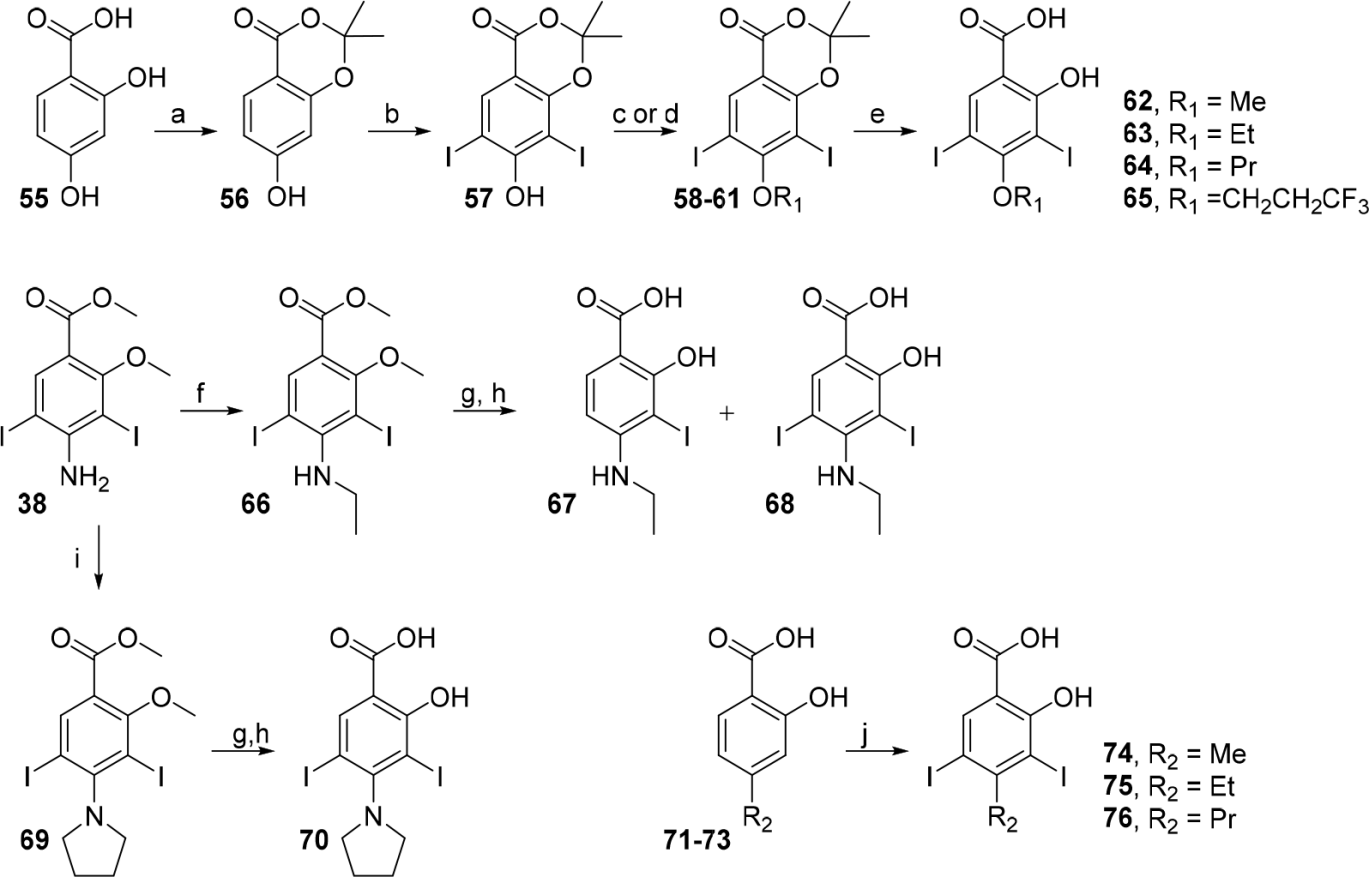
Synthetic Routes toward Subsite 3 Iodophenol Analogues Bearing Alkyl Chains Conditions: (a) acetone, TFAA, TFA, 0 to 25 °C, 24 h, 45%; (b) NIS, THF, 0 °C, 1 h, 73%; (c) R_1_OH, DIAD, PPh_3_, THF, 0 °C, 2.5 h, 63–68%; (d) EtI, Cs_2_CO_3_, DMF, 25 °C, 20 h, 57%; (e) 1 N aq. NaOH, THF, 25 °C, 1 h, 77–88%; (f) R-I Cs_2_CO_3_, DMF, 80 °C, 2–4 h, 57–72%; (g) BCl_3_, CH_2_Cl_2_, 0 to 25 °C, 30 min; (h) 1 N NaOH (aq.), MeOH, THF, 25 °C, 2–5 days, 33–64% over 2 steps, (i) I(CH_2_)_4_I, Cs_2_CO_3_, DMF, 80 °C, 2.5 h, 34%; (j) NIS, AcOH, 25 °C, 1 h, 88–98%.

Protected diiodosalicylic acid derivative **57** was prepared in 2 steps from resorcylic acid **55** by acetalization then iodination. Oxyether analogues were accessible in high yield by either alkylation or Mitsunobu coupling with phenol **57** followed by ester hydrolysis (Scheme 3). **57** was unreactive toward 1,1,1-trifluoro-2-haloethane electrophiles, and 2,2,2-trifluoroethanol is typically unreactive under Mitsunobu conditions.^62^ As an alternative, the 2,2,2-trifluoroethyl analogue **24** was prepared by full deprotonation and treatment under S_N_Ar conditions similar to those used for heterocycles (*vide supra*) (Scheme 1). **38** was readily alkylated to *N*-ethylaniline **66** using ethyl iodide. Despite extensive optimization attempts, **68** (and methyl and propyl analogues, data not shown) underwent partial *in situ* degradation during methoxy deprotection to the corresponding monoiodo byproduct **67**, although this was not observed for the pyrrolidine analogue **70**. **68** was thus tested as a mixture (9:1 diiodo:monoiodo) and the calculated (NMR) yield reported. *N*-Methyl and propyl analogues had worse profiles of deiodination and were deemed too low purity to give meaningful biophysical data. Commercial 4-alkylsalicylic acids **71**–**73** were iodinated using standard conditions in high yield to give analogues **74**–**76**.

### Biophysical Evaluation and SAR Studies

Our iodophenol library was assessed for thermal stabilization of p53-Y220C using differential scanning fluorimetry (DSF), and affinities (*K*_d_ values) of selected analogues with the highest thermal shifts (Δ*T*_m_) were then determined by isothermal titration calorimetry (ITC) (Table 1). Affinity (p*K*_d_) and Δ*T*_m_ showed generally good correlation, in line with our previous reports (Figure S3).^42^

**Table 1:**
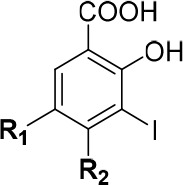

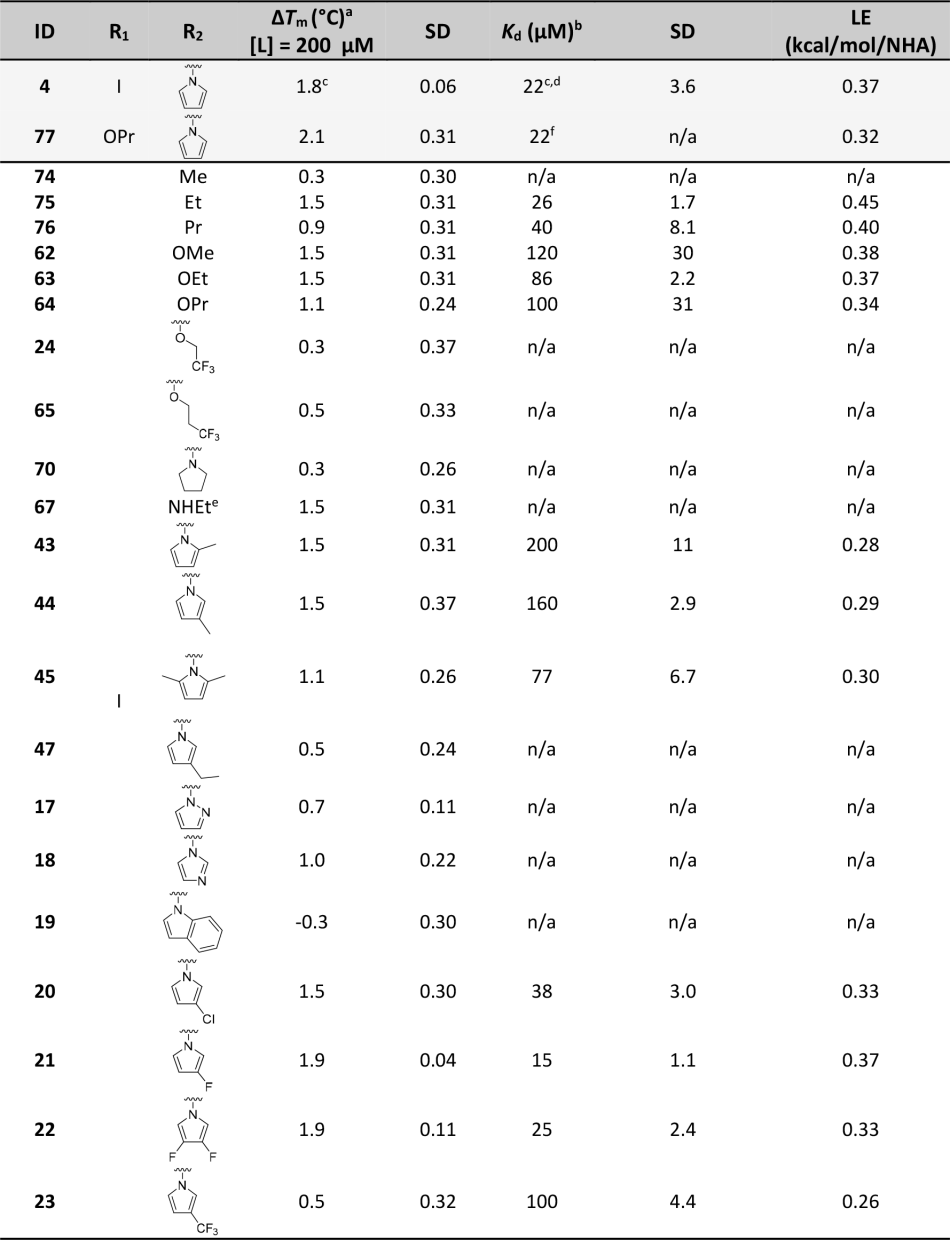
Thermal Stabilization (Δ*T*_m_) and Binding Affinities of Subsite 3 Targeting Analogues against p53-Y220C, Determined by DSF and ITC, Respectively (Previously Reported Analogues **4** and **77** Included as Positive Controls)

a Measured by DSF using 8 μM protein and 10× SYPRO Orange. Δ*T*_m_ values were calculated as the average of at least three independent measurements.

b Measured by ITC ([prot] = 50 μM). *K*_d_ values were calculated as the average of at least three independent measurements.

c Consistent with literature values Δ*T*_m_ = 1.8 °C ([L] = 250 μM), *K*_d_ = 21 μM.^44^

d Average of two repeats.

e Tested as a 90% pure mixture with monoiodinated analogue. *K*_d_ not recorded for mixture.

f Literature value.^42^ Abbreviations: SD, standard deviation; LE, ligand efficiency; NHA, number of heavy (non-hydrogen) atoms.

#### Short Alkyl Chains

Saturated pyrrolidine analogue **70** did not exhibit measurable binding nor thermal stabilization, possibly suggesting unfavorable steric constraints imposed by the 3D conformation of the pyrrolidine ring. Conversely, more flexible short alkyl groups linked by a *C*-, *N*-, or *O*- atom were widely tolerated. This is consistent with the over 10-fold affinity gain from addition of the 9-ethyl group to previously reported carbazole series.^51^ 2- and 3-atom chains gave the best affinities/thermal shifts (**62**, **63**, **67**, **75**, **76**), after which point a cutoff and a marked reduction in affinity/stabilization was observed (**64**, **75** vs **76**). Curiously, and despite the known fluorophilicity of the subsite 3 pocket, terminal trifluoromethylation of *O*-alkyl substituents abrogated affinity almost entirely in each case. This may be a result of conformational preorganization of the ether chain imparted by trifluoromethylation, contributing to an entropic penalty of binding, or steric clashes. The reduced affinity of oxyethers **62**–**64** compared with alkyl derivatives **75** and **76** can be ascribed to mesomeric donation of the ether oxygen onto the aromatic ring. Increasing electron density on the iodinated carbon is likely to impact the Lewis acidity of the C–I bond and therefore halogen bonding strength, which is closely related to affinity.^63^ Overall, all alkyl-based motifs investigated resulted in at least 4-fold decreased potency, with the exception of ethyl derivative **75** (*K*_d_ = 26 μM) that was similarly potent as the parent pyrrole derivative **4**.

#### Alkylpyrroles and Other N-Heteroaromatics

A methyl scan of the pyrrole moiety revealed that substitution was tolerated at the 2- and 3-positions, in addition to disubstitution at the 2- and 5-positions, although all resulting in reduced affinity and thermal stabilization compared with the unsubstituted pyrrole reference **4**. 2- and 3-methylpyrrole derivatives afforded 1.5 °C stabilization and bound approximately 7–9 times more weakly than **4** (*K*_d_ = 150–200 μM), while dimethylated **45** afforded weaker stabilization and 3-fold affinity reduction. These data likely indicate steric clashes between the ligand and the narrow subsite 3 pocket, and suggest a size limit for substituents at this position. 2-position substitution is predicted to impart a high degree of control over the dihedral angle between the pyrrole and phenyl groups, which may contribute to reduced shape complementarity between the ligand and the protein. This is further supported by results from bulkier 3-ethylpyrrole and indole derivatives **47** and **19**, which induced virtually no stabilization. The bulky indole group may be too sterically encumbered to fit in subsite 3, despite showing good shape complementarity in docking and crystal structure overlays (Figure 2D, Figure S1). The crystal structure of dianiline **7** suggests that accommodation of a benzene ring at this site requires rearrangement of the S7/S8 loop, which may disturb key contacts made by the iodophenol unit, including the key halogen bond to the L145 backbone carbonyl. Exchanging the pyrrole heterocycle with a pyrazole (**17**) or imidazole (**18**) also resulted in significantly lower affinity and stabilization, further highlighting the pyrrole as the heterocycle of choice toward subsite 3. This may be explained by the relatively hydrophobic nature of the subsite 3 pocket, which is well-known to bind nonpolar moieties like ethyl and pyrrole groups, leading to unfavorable interactions with the more polar pyrazole and imidazole groups.^44^

#### Halogenated Pyrroles

Pleasingly, halogenated pyrroles **20**–**22** bound with affinities in the low micromolar range (15–38 μM) and displayed strong stabilization of the mutant. Notably, 3-fluoropyrrole derivative **21** reproducibly displayed the highest protein stabilization and affinity from our library (Δ*T*_m_ = 1.9 °C, *K*_d_ = 15 μM) and represents a moderate improvement on the unsubstituted parent **4**. Difluoropyrrole **22** induced stabilization and affinity similar to those of monofluoropyrrole **21**. This is consistent with previous reports by us and colleagues on the fluorophilicity of the subsite 3.^45^ Terminal trifluoromethylation enhanced the binding affinity of previously reported carbazole lead series by approximately 5-fold, via interactions with residues C220 (S–F), L145 ((O)C–F), and W146 ((O)C–F) (Figure 2B,C). Conversely, trifluoromethylpyrrole analogue **23** showed significantly lower stabilization and approximately 5-fold reduction in binding affinity compared with unsubstituted reference **4**, possibly again reflecting a size limit for substituents targeting subsite 3. It is likely that the dihedral angle (C–N_pyrrole_–C–C_Ph_, θ > 80°) imposed by the biaryl scaffold prevents the pyrrole accessing a suitable orientation for the trifluoromethyl group to engage these residues.

**Figure 3:**
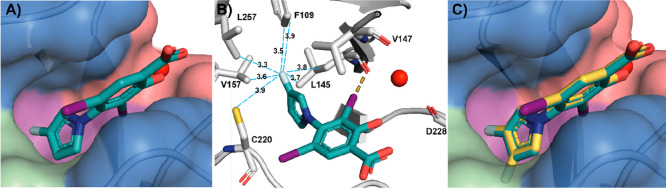
(A) Bound structure of **21** (turquoise sticks, PDB: 8A31) with p53-Y220C (surface representation). (B) Key subsite 3 residues and S–F and C–F distances (Å) are shown (blue dashes). The halogen bond to L145 is shown in orange. (C) Overlaid co-crystal structures of **21** (turquoise sticks) and difluorinated **22** (yellow sticks, PDB: 8A32, chain B) with p53-Y220C (surface representation).

We determined high-resolution (≤1.5 Å) crystal structures of Y220C with bound mono- and difluorinated derivatives **21** and **22** (Figure 3A–C; PDB: 8A31, 8A32). These experimental structures unambiguously confirmed that their binding mode was virtually identical to that of unsubstituted parent compound **4**, consistent with the computationally predicted structures (Figure S1, Figure S4). Interestingly, a preferential orientation of the 1-aryl-3-fluoropyrrole in the pocket as the a*R* atropisomer was observed. In this preferred axially chiral conformation, the fluorine engages in hydrophobic contacts with F109, L145, V157, and L257. C220 adopts a “flipped” conformation with the sulfur atom 3.9 Å from the fluorine, probably precluding fluorine–sulfur contacts due to the positioning of the biaryl scaffold. The a*R* conformer was the only one observable in chain B of the asymmetric unit, with the a*S* conformer only detected as a minor conformation in the electron density in chain A at ca. 25% occupancy (Figure S5). There was clear electron density for both fluorine atoms of **22** in the bound structure, and they occupied almost identical positions as observed for monofluorinated **21**.

### Lead Optimization: SAR Transfer to Aminobenzothiazole Leads

Fluorination of the 3-position of the pyrrole provided a modest although reproducible enhancement of Y220C thermal stabilization. We sought to introduce this optimized motif to the more potent benzothiazole scaffold along with 3-chloro and 3,4-difluoro analogues for comparison.

**Scheme 4:**
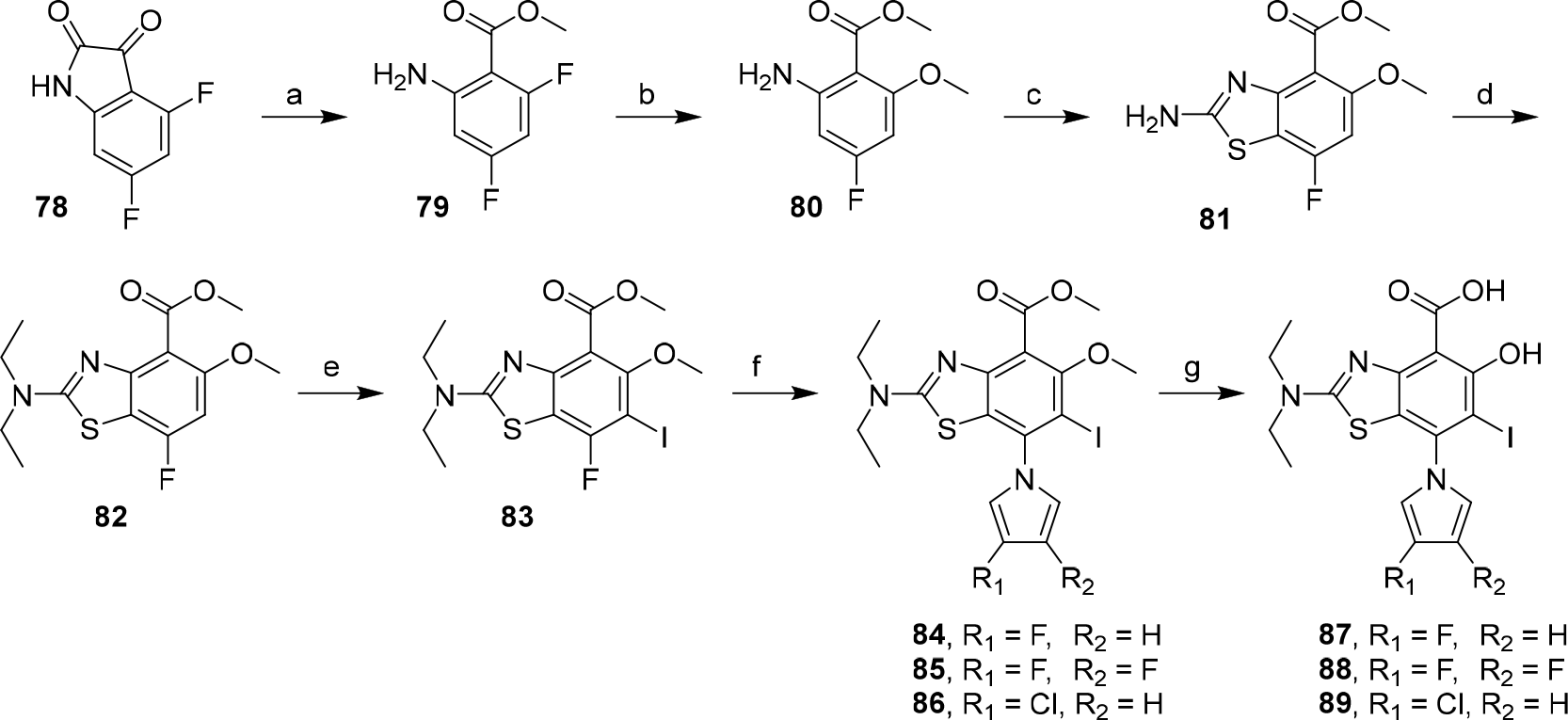
Synthetic Route toward Novel Subsite 3 Aminobenzothiazole Derivatives Conditions: (a) *t*BuOOH, Cs_2_CO_3_, MeOH, 20–30 °C, 2 h, 81%; (b) NaOMe, MeOH, 1,4-dioxane, 80 °C, 2 h, 70%; (c) KSCN, Br_2_, AcOH, 10 to 65 °C, 2.5 h, 61%; (d) CH_3_CH_2_Br, Cs_2_CO_3_, DMF, 60 °C, 4 h, 57%; (e) NIS, AcOH, 25 °C, 1 h, 96%; (f) *N*-TIPS-pyrrole derivative (**27**, **28**, **30**), KF, Cs_2_CO_3_, DMSO, 70 °C, 1 h, 50–73%; (g) BBr_3_, CH_2_Cl_2_, 0 to 25 °C, 24 h, 73–79%.

The synthetic route toward the target aminobenzothiazole derivatives is shown in Scheme 4. To access subsite 3 analogues of chemical probe MB710 (**2**), we devised a new route that allowed mild, late-stage derivatization at the benzothiazole 7-position using the S_N_Ar protocol optimized on the diiodo library (*vide supra*). The critical aryl fluoride intermediate **83** could be accessed from anthranillic ester building block **80**, which was prepared in 2 steps from commercial isatin **78**.

Oxidative ring-opening of isatin **78** with TBHP in methanol afforded ester **79** in good yield, which was converted to **80** by regioselective S_N_Ar using NaOMe in dioxane.^64,65^ Subsequent treatment with bromine/KSCN gave protected aminobenzothiazole **81** in 61% yield. Alkylation of intermediate **81** with bromoethane followed by ring iodination afforded precursor **83** for subsite 3 derivatization. Late-stage installation of substituted pyrroles was effected by *in situ* desilylation and S_N_Ar using 1-TIPS-pyrroles, affording analogues **84**–**86** in yields ranging from 50 to 73%. Finally, protected aminobenzothiazoles **84**–**86** could be converted to their corresponding salicylic acid derivatives **87**–**89** by demethylation with BBr_3_. Critically, our new route offers convergent access to late-stage derivatized subsite 3 analogues of MB710 in up to 10% overall yield, marking a significant improvement to the linear route to **2** (1% overall yield).

The new aminobenzothiazoles stabilized the Y220C mutant by over 2.0 °C at 200 μM concentration in DSF measurements (Table 2). In line with the SAR data gathered in Table 1, 3-chloro analogue **89** displayed slightly weaker (ca. 2-fold) binding affinity and stabilization compared with parent compound MB710 (**2**). Pleasingly, 3-fluoro derivative **87** displayed an approximately 4-fold improvement in affinity (*K*_d_ = 320 nM) compared with parent **2**, while maintaining a comparable ligand efficiency. Difluorinated analogue **88** was similarly potent, showing a consistent effect of pyrrole fluorination on *in vitro* potency. Overall, **87** and **88** are the first ligands reported in the scientific literature binding to the Y220C pocket with sub-micromolar *in vitro* affinity, marking an important milestone in the search of drug candidates targeting p53-Y220C in cancer.

**Table 2:**
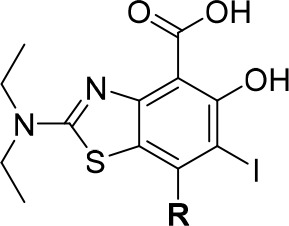

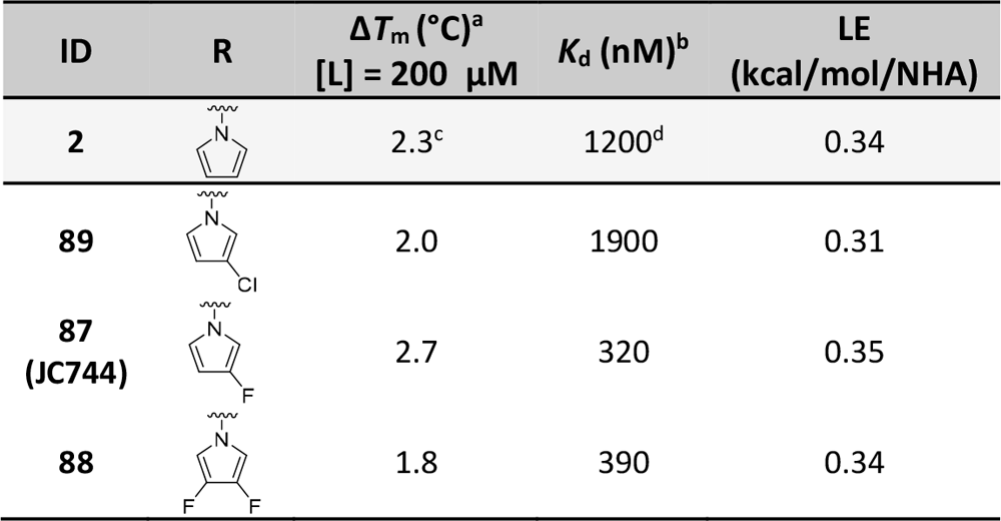
Thermal Shift and ITC Data for Aminobenzothiazole Analogues **2** and **87**–**89** against p53-Y220C

a Measured by DSF using 8 μM protein and 10× SYPRO Orange.

b Measured by ITC (reverse titrations) using 4–15 μM ligand and 100–147 μM protein. Δ*T*_m_ and *K*_d_ values were calculated as the average of at least three independent measurements.

c Consistent with literature value Δ*T*_m_ = 2.0 °C ([L] = 250 μM).

d Lit.^42^*K*_d_ = 4.1 μM.

## Conclusions

We report the SAR evaluation of libraries of small-molecule stabilizers of the p53 cancer mutant Y220C and new convergent synthetic routes to access MB710 (**2**) analogues targeting the subsite 3 sub-pocket. This subsite has remained relatively unexplored in terms of lead optimization, notably due to the lack of suitable synthetic routes to derivatize existing lead compounds. SAR development using a diiodinated salicylate model system identified several halogenated pyrrole motifs as promising substructures to target the buried subsite 3 in the Y220C pocket. This was supported by high-resolution X-ray crystal structures of fluoropyrrole derivatives, highlighting fluorine–protein contacts in subsite 3. Incorporation into the more potent aminobenzothiazole scaffold and biophysical evaluation identified two fluorinated derivatives displaying sub-micromolar binding affinity. In particular, new chemical probe JC744 (**87**) potently stabilizes Y220C *in vitro* and displays Δ*T*_m_ = 2.7 °C and *K*_d_ = 320 nM (ITC), around 4 times more potent than the non-fluorinated analogue MB710. To our knowledge, this is the first example of a nanomolar ligand of p53-Y220C in the scientific literature, and this represents an important step toward novel, potent classes of Y220C ligands for clinical evaluation in oncology. Noteworthy, the S_N_Ar chemistry we describe led to significant improvements to the scalability, tractability (convergence), and yield (7 steps, ∼10% overall yield) compared with previous routes toward MB710 (9 steps, <1% overall yield). Furthermore, this new chemistry also promises to unlock access to new derivatives to target other Y220 mutants, including Y220S and Y220N, which together account for another 10,000–20,000 new cancer cases per year worldwide.^22^ Overall, this study validates two new chemical probes for the prominent p53 cancer mutant Y220C with nanomolar *in vitro* binding affinity and opens exciting opportunities for chemically addressing a range of p53-Y220X mutants for which high-affinity ligands currently do not exist.

## Supporting Information Available

Synthetic procedures and compound characterization data, protocols for recombinant protein production, protocols for docking studies, protocols for biophysical studies, ITC titrations of small molecules, X-ray crystallographic data, and representative HPLC traces (PDF)
Molecular formula strings (CSV)

## Accession Codes

The atomic coordinates and structure factors of the Y220C mutant in complex with **21** and **22** have been deposited in the Protein Data Bank (PDB) under accession codes 8A31 and 8A32, respectively.

## Notes

Milligram quantities of the small molecules described in this manuscript are available upon request from the corresponding author.

## Abbreviations Used

μM: micromolar
DBD: DNA-binding domain
DSF: differential scanning fluorimetry
ITC: isothermal titration calorimetry
kcal: kilocalorie
K_d_: dissociation constant
LE: ligand efficiency
nM: nanomolar
NHA: non-hydrogen atom
SD: standard deviation
S_*N*_Ar: nucleophilic aromatic substitution
*T*_m_: protein melting temperature

## References

[ref1] Hainaut P.; Hollstein M. p53 and Human Cancer: The First Ten Thousand Mutations. In Advances in cancer research; Vande Woude G. F.; Klein G., Eds.; Academic Press, 1999; Vol. 77, pp 81–137. 10.1016/s0065-230x(08)60785-x 10549356

[ref2] Lane D. P. p53, guardian of the genome. Nature 1992, 358 (6381), 15–6. 10.1038/358015a0. 1614522

[ref3] Vogelstein B.; Lane D.; Levine A. J. Surfing the p53 network. Nature 2000, 408 (6810), 307–310. 10.1038/35042675. 11099028

[ref4] Joerger A. C.; Fersht A. R. The p53 Pathway: Origins, Inactivation in Cancer, and Emerging Therapeutic Approaches. Annu. Rev. Biochem. 2016, 85, 375–404. 10.1146/annurev-biochem-060815-014710. 27145840

[ref5] Sung H.; Ferlay J.; Siegel R. L.; Laversanne M.; Soerjomataram I.; Jemal A.; Bray F. Global Cancer Statistics 2020: GLOBOCAN Estimates of Incidence and Mortality Worldwide for 36 Cancers in 185 Countries. CA: A Cancer Journal for Clinicians 2021, 71 (3), 209–249. 10.3322/caac.21660. 33538338

[ref6] Leroy B.; Anderson M.; Soussi T. TP53 Mutations in Human Cancer: Database Reassessment and Prospects for the Next Decade. Human Mutation 2014, 35 (6), 672–688. 10.1002/humu.22552. 24665023

[ref7] Joerger A. C.; Fersht A. R. Structure-function-rescue: the diverse nature of common p53 cancer mutants. Oncogene 2007, 26 (15), 2226–2242. 10.1038/sj.onc.1210291. 17401432

[ref8] Duffy M. J.; Synnott N. C.; McGowan P. M.; Crown J.; O’Connor D.; Gallagher W. M. p53 as a target for the treatment of cancer. Cancer Treatment Reviews 2014, 40 (10), 1153–60. 10.1016/j.ctrv.2014.10.004. 25455730

[ref9] Brown C. J.; Lain S.; Verma C. S.; Fersht A. R.; Lane D. P. Awakening guardian angels: drugging the p53 pathway. Nature Reviews Cancer 2009, 9 (12), 862–73. 10.1038/nrc2763. 19935675

[ref10] Joerger A. C.; Fersht A. R. The tumor suppressor p53: from structures to drug discovery. Cold Spring Harbor Perspectives in Biology 2010, 2 (6), a000919–a000919. 10.1101/cshperspect.a000919. 20516128 PMC2869527

[ref11] Petitjean A.; Mathe E.; Kato S.; Ishioka C.; Tavtigian S. V.; Hainaut P.; Olivier M. Impact of mutant p53 functional properties on TP53 mutation patterns and tumor phenotype: lessons from recent developments in the IARC TP53 database. Human Mutation 2007, 28 (6), 622–629. 10.1002/humu.20495. 17311302

[ref12] de Oliveira A. P. G.; Petronilho C. E.; Pedrote M. M.; Marques A. M.; Vieira C. R. G. T.; Cino A. E.; Silva L. J. The Status of p53 Oligomeric and Aggregation States in Cancer. Biomolecules 2020, 10 (4), 548 10.3390/biom10040548. PMC7226498 32260447

[ref13] Xu J.; Reumers J.; Couceiro J. R.; De Smet F.; Gallardo R.; Rudyak S.; Cornelis A.; Rozenski J.; Zwolinska A.; Marine J.-C.; Lambrechts D.; Suh Y.-A.; Rousseau F.; Schymkowitz J. Gain of function of mutant p53 by coaggregation with multiple tumor suppressors. Nat. Chem. Biol. 2011, 7, 285–295. 10.1038/nchembio.546. 21445056

[ref14] Dittmer D.; Pati S.; Zambetti G.; Chu S.; Teresky A. K.; Moore M.; Finlay C.; Levine A. J. Gain of function mutations in p53. Nature genetics 1993, 4 (1), 42–46. 10.1038/ng0593-42. 8099841

[ref15] Oren M.; Rotter V. Mutant p53 gain-of-function in cancer. Cold Spring Harbor Perspectives in Biology 2010, 2 (2), a001107–a001107. 10.1101/cshperspect.a001107. 20182618 PMC2828285

[ref16] Stiewe T.; Haran T. E. How mutations shape p53 interactions with the genome to promote tumorigenesis and drug resistance. Drug Resistance Updates: Reviews and Commentaries in Antimicrobial and Anticancer Chemotherapy 2018, 38, 27–43. 10.1016/j.drup.2018.05.001. 29857816

[ref17] Wang G.; Fersht A. R. Multisite aggregation of p53 and implications for drug rescue. Proc. Natl. Acad. Sci. U. S. A. 2017, 114 (13), E2634–E2643. 10.1073/pnas.1700308114. 28292898 PMC5380089

[ref18] Bullock A. N.; Henckel J.; Fersht A. R. Quantitative analysis of residual folding and DNA binding in mutant p53 core domain: definition of mutant states for rescue in cancer therapy. Oncogene 2000, 19 (10), 1245–1256. 10.1038/sj.onc.1203434. 10713666

[ref19] Bullock A. N.; Henckel J.; DeDecker B. S.; Johnson C. M.; Nikolova P. V.; Proctor M. R.; Lane D. P.; Fersht A. R. Thermodynamic stability of wild-type and mutant p53 core domain. Proc. Natl. Acad. Sci. U.S.A. 1997, 94 (26), 14338–14342. 10.1073/pnas.94.26.14338. 9405613 PMC24967

[ref20] Dearth L. R.; Qian H.; Wang T.; Baroni T. E.; Zeng J.; Chen S. W.; Yi S. Y.; Brachmann R. K. Inactive full-length p53 mutants lacking dominant wild-type p53 inhibition highlight loss of heterozygosity as an important aspect of p53 status in human cancers. Carcinogenesis 2007, 28 (2), 289–298. 10.1093/carcin/bgl132. 16861262

[ref21] Di Como C. J.; Prives C. Human tumor-derived p53 proteins exhibit binding site selectivity and temperature sensitivity for transactivation in a yeast-based assay. Oncogene 1998, 16 (19), 2527–2539. 10.1038/sj.onc.1202041. 9627118

[ref22] Bauer M. R.; Krämer A.; Settanni G.; Jones R. N.; Ni X.; Khan Tareque R.; Fersht A. R.; Spencer J.; Joerger A. C. Targeting Cavity-Creating p53 Cancer Mutations with Small-Molecule Stabilizers: the Y220X Paradigm. ACS Chem. Biol. 2020, 15 (3), 657–668. 10.1021/acschembio.9b00748. 31990523 PMC7307883

[ref23] Bouaoun L.; Sonkin D.; Ardin M.; Hollstein M.; Byrnes G.; Zavadil J.; Olivier M. TP53 Variations in Human Cancers: New Lessons from the IARC TP53 Database and Genomics Data. Human Mutation 2016, 37 (9), 865–876. 10.1002/humu.23035. 27328919

[ref24] Bauer M. R.; Jones R. N.; Tareque R. K.; Springett B.; Dingler F. A.; Verduci L.; Patel K. J.; Fersht A. R.; Joerger A. C.; Spencer J. A structure-guided molecular chaperone approach for restoring the transcriptional activity of the p53 cancer mutant Y220C. Future Medicinal Chemistry 2019, 11 (19), 2491–2504. 10.4155/fmc-2019-0181. 31633398 PMC6803818

[ref25] Joerger A. C.; Ang H. C.; Fersht A. R. Structural basis for understanding oncogenic p53 mutations and designing rescue drugs. Proc. Natl. Acad. Sci. U.S.A. 2006, 103 (41), 15056–15061. 10.1073/pnas.0607286103. 17015838 PMC1635156

[ref26] Hartl F. U.; Bracher A.; Hayer-Hartl M. Molecular chaperones in protein folding and proteostasis. Nature 2011, 475 (7356), 324–332. 10.1038/nature10317. 21776078

[ref27] Müller P.; Ceskova P.; Vojtesek B. Hsp90 Is Essential for Restoring Cellular Functions of Temperature-sensitive p53 Mutant Protein but Not for Stabilization and Activation of Wild-type p53: IMPLICATIONS FOR CANCER THERAPY. J. Biol. Chem. 2005, 280 (8), 6682–6691. 10.1074/jbc.M412767200. 15613472

[ref28] Alex Merrick B.; He C.; Witcher L. L.; Patterson R. M.; Reid J. J.; Miki Pence-Pawlowski P.; Selkirka J. K. HSP binding and mitochondrial localization of p53 protein in human HT1080 and mouse C3H10T1/2 cell lines. Biochimica et Biophysica Acta (BBA) - Protein Structure and Molecular Enzymology 1996, 1297 (1), 57–68. 10.1016/0167-4838(96)00089-1. 8841381

[ref29] Chasov V.; Mirgayazova R.; Zmievskaya E.; Khadiullina R.; Valiullina A.; Stephenson Clarke J.; Rizvanov A.; Baud M. G. J.; Bulatov E. Key Players in the Mutant p53 Team: Small Molecules, Gene Editing, Immunotherapy. Frontiers in Oncology 2020, 10, 1460 10.3389/fonc.2020.01460. 32974171 PMC7461930

[ref30] Yee K.; Martinelli G.; Assouline S.; Kasner M.; Vey N.; Kelly K. R.; Drummond M. W.; Dennis M.; Seiter K.; Blotner S.; Jukofsky L.; Middleton S.; Zhi J.; Chen G.; Zhong H.; Nichols G. Phase 1b Study Of The MDM2 Antagonist RG7112 In Combination With 2 Doses/Schedules Of Cytarabine. Blood 2013, 122 (21), 498–498. 10.1182/blood.V122.21.498.498.

[ref31] Cluzeau T.; Sebert M.; Rahmé R.; Cuzzubbo S.; Walter-petrich A.; Lehmann che J.; Peterlin P.; Beve B.; Attalah H.; Chermat F.; Miekoutima E.; Beyne-Rauzy O.; Recher C.; Stamatoullas A.; Willems L.; Raffoux E.; Berthon C.; Quesnel B.; Carpentier A.; Sallman D. A.; Chevret S.; Ades L.; Fenaux P. APR-246 Combined with Azacitidine (AZA) in TP53 Mutated Myelodysplastic Syndrome (MDS) and Acute Myeloid Leukemia (AML). a Phase 2 Study By the Groupe Francophone Des Myélodysplasies (GFM). Blood 2019, 134 (Suppl-1), 677–677. 10.1182/blood-2019-125579.

[ref32] Kogan S.; Carpizo D. R. Zinc Metallochaperones as Mutant p53 Reactivators: A New Paradigm in Cancer Therapeutics. Cancers 2018, 10 (6), 166 10.3390/cancers10060166. PMC6025018 29843463

[ref33] Salim K. Y.; Maleki Vareki S.; Danter W. R.; Koropatnick J. COTI-2, a new anticancer drug currently under clinical investigation, targets mutant p53 and negatively modulates the PI3K/AKT/mTOR pathway. Eur. J. Cancer 2016, 69, S19 10.1016/S0959-8049(16)32638-7.

[ref34] Burgess A.; Chia K. M.; Haupt S.; Thomas D.; Haupt Y.; Lim E. Clinical Overview of MDM2/X-Targeted Therapies. Frontiers in Oncology 2016, 6, 7 10.3389/fonc.2016.00007. 26858935 PMC4728205

[ref35] de Weger V. A.; de Jonge M.; Langenberg M. H. G.; Schellens J. H. M.; Lolkema M.; Varga A.; Demers B.; Thomas K.; Hsu K.; Tuffal G.; Goodstal S.; Macé S.; Deutsch E. A phase I study of the HDM2 antagonist SAR405838 combined with the MEK inhibitor pimasertib in patients with advanced solid tumours. Br. J. Cancer 2019, 120 (3), 286–293. 10.1038/s41416-018-0355-8. 30585255 PMC6354023

[ref36] Silva J. L.; Lima C. G. S.; Rangel L. P.; Ferretti G. D. S.; Pauli F. P.; Ribeiro R. C. B.; da Silva T. d. B.; da Silva F. C.; Ferreira V. F. Recent Synthetic Approaches towards Small Molecule Reactivators of p53. Biomolecules 2020, 10 (4), 635 10.3390/biom10040635. PMC7226499 32326087

[ref37] Khurana A.; Shafer D. A. MDM2 antagonists as a novel treatment option for acute myeloid leukemia: perspectives on the therapeutic potential of idasanutlin (RG7388). Oncol. Targets Ther. 2019, 12, 2903–2910. 10.2147/OTT.S172315. PMC6563714 31289443

[ref38] Bykov V. J.; Wiman K. G. Mutant p53 reactivation by small molecules makes its way to the clinic. FEBS Lett. 2014, 588 (16), 2622–7. 10.1016/j.febslet.2014.04.017. 24768524

[ref39] Bauer M. R.; Joerger A. C.; Fersht A. R. 2-Sulfonylpyrimidines: Mild alkylating agents with anticancer activity toward p53-compromised cells. Proc. Natl. Acad. Sci. U.S.A. 2016, 113 (36), E5271–E5280. 10.1073/pnas.1610421113. 27551077 PMC5018792

[ref40] Tang Y.; Song H.; Wang Z.; Xiao S.; Xiang X.; Zhan H.; Wu L.; Wu J.; Xing Y.; Tan Y.; Liang Y.; Yan N.; Li Y.; Li J.; Wu J.; Zheng D.; Jia Y.; Chen Z.; Li Y.; Zhang Q.; Zhang J.; Zeng H.; Tao W.; Liu F.; Wu Y.; Lu M. Repurposing antiparasitic antimonials to noncovalently rescue temperature-sensitive p53 mutations. Cell Reports 2022, 39 (2), 110622 10.1016/j.celrep.2022.110622. 35417717

[ref41] Chen S.; Wu J. L.; Liang Y.; Tang Y. G.; Song H. X.; Wu L. L.; Xing Y. F.; Yan N.; Li Y. T.; Wang Z. Y.; Xiao S. J.; Lu X.; Chen S. J.; Lu M. Arsenic Trioxide Rescues Structural p53 Mutations through a Cryptic Allosteric Site. Cancer Cell 2021, 39 (2), 225–239.e8. 10.1016/j.ccell.2020.11.013. 33357454

[ref42] Baud M. G. J.; Bauer M. R.; Verduci L.; Dingler F. A.; Patel K. J.; Horil Roy D.; Joerger A. C.; Fersht A. R. Aminobenzothiazole derivatives stabilize the thermolabile p53 cancer mutant Y220C and show anticancer activity in p53-Y220C cell lines. Eur. J. Med. Chem. 2018, 152, 101–114. 10.1016/j.ejmech.2018.04.035. 29702446 PMC5986712

[ref43] Wilcken R.; Liu X.; Zimmermann M. O.; Rutherford T. J.; Fersht A. R.; Joerger A. C.; Boeckler F. M. Halogen-enriched fragment libraries as leads for drug rescue of mutant p53. J. Am. Chem. Soc. 2012, 134 (15), 6810–6818. 10.1021/ja301056a. 22439615 PMC3789257

[ref44] Joerger A. C.; Bauer M. R.; Wilcken R.; Baud M. G. J.; Harbrecht H.; Exner T. E.; Boeckler F. M.; Spencer J.; Fersht A. R. Exploiting Transient Protein States for the Design of Small-Molecule Stabilizers of Mutant p53. Structure 2015, 23 (12), 2246–2255. 10.1016/j.str.2015.10.016. 26636255 PMC4671956

[ref45] Bauer M. R.; Jones R. N.; Baud M. G.; Wilcken R.; Boeckler F. M.; Fersht A. R.; Joerger A. C.; Spencer J. Harnessing Fluorine-Sulfur Contacts and Multipolar Interactions for the Design of p53 Mutant Y220C Rescue Drugs. ACS Chem. Biol. 2016, 11 (8), 2265–2274. 10.1021/acschembio.6b00315. 27267810 PMC4994063

[ref46] Swallow S. Fluorine in Medicinal Chemistry. Progress in Medicinal Chemistry 2015, 54, 65–133. 10.1016/bs.pmch.2014.11.001. 25727703

[ref47] Maria; Ayub K. Aromaticities of Five Membered Heterocycles through Dimethyldihydropyrenes Probe by Magnetic and Geometric Criteria. Journal of Chemistry 2015, 2015, 456961 10.1155/2015/456961.

[ref48] Mucsi Z.; Viskolcz B.; Csizmadia I. G. A Quantitative Scale for the Degree of Aromaticity and Antiaromaticity: A Comparison of Theoretical and Experimental Enthalpies of Hydrogenation. J. Phys. Chem. A 2007, 111 (6), 1123–1132. 10.1021/jp0657686. 17286363

[ref49] Dalvie D. K.; Kalgutkar A. S.; Khojasteh-Bakht S. C.; Obach R. S.; O’Donnell J. P. Biotransformation Reactions of Five-Membered Aromatic Heterocyclic Rings. Chem. Res. Toxicol. 2002, 15 (3), 269–299. 10.1021/tx015574b. 11896674

[ref50] Woller E. K.; Smirnov V. V.; DiMagno S. G. A Straightforward Synthesis of 3,4-Difluoropyrrole. Journal of Organic Chemistry 1998, 63 (16), 5706–5707. 10.1021/jo9805798.

[ref51] Boeckler F. M.; Joerger A. C.; Jaggi G.; Rutherford T. J.; Veprintsev D. B.; Fersht A. R. Targeted rescue of a destabilized mutant of p53 by an in silico screened drug. Proc. Natl. Acad. Sci. U.S.A. 2008, 105 (30), 10360–10365. 10.1073/pnas.0805326105. 18650397 PMC2492497

[ref52] Friesner R. A.; Banks J. L.; Murphy R. B.; Halgren T. A.; Klicic J. J.; Mainz D. T.; Repasky M. P.; Knoll E. H.; Shelley M.; Perry J. K.; Shaw D. E.; Francis P.; Shenkin P. S. Glide: A New Approach for Rapid, Accurate Docking and Scoring. 1. Method and Assessment of Docking Accuracy. J. Med. Chem. 2004, 47 (7), 1739–1749. 10.1021/jm0306430. 15027865

[ref53] Barnes K. D.; Hu Y.; Hunt D. A. Electrophilic Fluorination of a Highly Functionalized Pyrrole. Synth. Commun. 1994, 24 (12), 1749–1755. 10.1080/00397919408010180.

[ref54] Bray B. L.; Mathies P. H.; Naef R.; Solas D. R.; Tidwell T. T.; Artis D. R.; Muchowski J. M. N-(Triisopropylsilyl)pyrrole. A progenitor ″par excellence″ of 3-substituted pyrroles. Journal of Organic Chemistry 1990, 55 (26), 6317–6328. 10.1021/jo00313a019.

[ref55] Thomassen I. K.; Vazquez-Lima H.; Gagnon K. J.; Ghosh A. Octaiodoporphyrin. Inorg. Chem. 2015, 54 (23), 11493–11497. 10.1021/acs.inorgchem.5b02127. 26571017

[ref56] Higashino T.; Osuka A. 2,3,17,18-Tetrahalohexaphyrins and the First Phlorin-type Hexaphyrins. Chemistry - An Asian Journal 2013, 8 (9), 1994–2002. 10.1002/asia.201300474. 23780790

[ref57] Leroy J. Improved synthesis of 3-(trifluoromethyl)pyrrole. J. Fluorine Chem. 1991, 53 (1), 61–70. 10.1016/S0022-1139(00)82239-3.

[ref58] Bucci R.; Laguzzi G.; Pompili M. L.; Speranza M. Gas-phase heteroaromatic substitution. 12. Reaction of free trifluoromethyl cation with simple five-membered heteroarenes in the gas phase. J. Am. Chem. Soc. 1991, 113 (12), 4544–4550. 10.1021/ja00012a024.

[ref59] Naumann D.; Kischkewitz J. Trifluoromethylierungsreaktionen von CF3I, Te(CF3)2, Sb(CF3)3, Hg(CF3)2, und Cd(CF3)2·D mit furan, Thiophen, Pyrrol und p-Benzochinon [1]. J. Fluorine Chem. 1990, 46 (2), 265–281. 10.1016/S0022-1139(00)80995-1.

[ref60] Shaitanova E. N.; Gerus I. I.; Kukhar V. P. A new synthetic route to 3-polyfluoroalkyl-containing pyrroles. Tetrahedron Lett. 2008, 49 (7), 1184–1187. 10.1016/j.tetlet.2007.12.048.

[ref61] Walden D. M.; Jaworski A. A.; Johnston R. C.; Hovey M. T.; Baker H. V.; Meyer M. P.; Scheidt K. A.; Cheong P. H.-Y. Formation of Aza-ortho-quinone Methides Under Room Temperature Conditions: Cs2CO3 Effect. Journal of Organic Chemistry 2017, 82 (14), 7183–7189. 10.1021/acs.joc.7b00697. 28649838

[ref62] Shen X.; Neumann C. N.; Kleinlein C.; Goldberg N. W.; Ritter T. Alkyl Aryl Ether Bond Formation with PhenoFluor. Angewandte Chemie (International ed. in English) 2015, 54 (19), 5662–5665. 10.1002/anie.201500902. 25800679 PMC4487836

[ref63] Roughley S. D.; Jordan A. M. The Medicinal Chemist’s Toolbox: An Analysis of Reactions Used in the Pursuit of Drug Candidates. J. Med. Chem. 2011, 54 (10), 3451–3479. 10.1021/jm200187y. 21504168

[ref64] Wang Y.-W.; Zheng L.; Jia F.-C.; Chen Y.-F.; Wu A.-X. Oxidative ring-opening of isatins for the synthesis of 2-aminobenzamides and 2-aminobenzoates. Tetrahedron 2019, 75 (11), 1497–1503. 10.1016/j.tet.2019.01.067.

[ref65] Wendt M. D.; Kunzer A. R. Ortho-selectivity in SNAr substitutions of 2,4-dihaloaromatic compounds. Reactions with anionic nucleophiles. Tetrahedron Lett. 2010, 51 (23), 3041–3044. 10.1016/j.tetlet.2010.03.124.

