# Supplementary material for: Discovery of Nanomolar-Affinity Pharmacological Chaperones Stabilizing the Oncogenic p53 Mutant Y220C
Source: ACS Pharmacol Transl Sci. 2022 Oct 11;5(11):1169–80. doi: 10.1021/acsptsci.2c00164 (PMC9667543; doi:10.1021/acsptsci.2c00164)
Supplement: Supplementary file 1 — pt2c00164_si_001.pdf [file pt2c00164_si_001.pdf]

Supporting Information

Discovery of Nanomolar Affinity Pharmacological Chaperones Stabilizing the Oncogenic p53 Mutant Y220C

Joseph R. Stephenson Clarke,[a] Leon R. Douglas,[b] Patrick J. Duriez,[c] Dimitrios-Ilias Balourdas,[d] Andreas C. Joerger,[d] Raniya Khadiullina,[e] Emil Bulatov,[e] and Matthias G. J. Baud*[a]

[a] School of Chemistry and Institute for Life Sciences, University of Southampton, Southampton SO17 1BJ, United Kingdom

[b] Cancer Research UK, Somers Building, University Hospital Southampton, Tremona Road, Southampton SO16 6YD, United Kingdom

[c] Centre for Cancer Immunology, University Hospital Southampton, Coxford Road, Southampton SO16 6YD, United Kingdom

[d] i) Institute of Pharmaceutical Chemistry, Johann Wolfgang Goethe University, Max-von-Laue-Str. 9, 60438 Frankfurt am Main, Germany. ii) Buchmann Institute for Molecular Life Sciences and Structural Genomics Consortium (SGC), Max-von-Laue-Str. 15, 60438, Frankfurt am Main, Germany.

[e] Institute of Fundamental Medicine and Biology, Kazan Federal University, Kazan, Russia

*To whom correspondence should be addressed: m.baud@soton.ac.uk

Molecular Docking

Chemical structures were imported to Maestro (Schrödinger) as .sdf files and prepared using LigPrep. Receptors were generated by importing the available protein crystal structure of MB710 (**2**) (PDB:5O1I) as a .pdb file, using the Protein Preparation Wizard and defining the receptor using Receptor Grid Generation. Prepared ligands were then docked using XP precision Glide to generate docking poses and analyzed visually using Maestro.[1-5]

Protein Expression and Purification

Stabilized p53-Y220C DBD (residues 94−312) was expressed and purified as previously described.[32,49,50] Briefly, the *N*-terminal fusion protein (6xHis/lipoyl domain/TEV protease cleavage site) was overexpressed using *E. coli* Rosetta2 plac I in Terrific Broth medium at 22 °C for 16 h and purified using standard Ni-affinity chromatography protocols. After overnight digestion with TEV protease, the p53-Y220C DBD was further purified using a Heparin column and dialyzed against 25 mM KPi (pH 7.2), 150 mM NaCl, and 1 mM TCEP buffer. The purification protocol for crystallography included an additional gel filtration step after the Heparin column (Superdex 75; buffer: 25 mM KPi (pH 7.2), 150 mM NaCl, and 0.5 mM TCEP). Molecular weight and protein purity (>95%) were confirmed via SDS gel electrophoresis and ESI–MS.

Protein Crystallization and Structure Determination

Crystals of the stabilized Y220C mutant DBD were grown at 20 °C using the sitting drop vapor diffusion technique by mixing equal amounts of protein solution (5.7 mg mg/ml mutant protein in 25 mM phosphate buffer, pH 7.2, 150 mM NaCl, and 0.5 mM TCEP) and reservoir buffer (19% (w/v) polyethylene glycol 4000, 100 mM HEPES, pH 7.2, and 5 mM DTT). They were soaked for 4 h in a saturated solution of compound **21** or **22** (30 mM) in cryo buffer (19% [w/v] polyethylene glycol 4000, 20% [v/v] glycerol, 100 mM Tris, pH 7.2, 10 mM sodium phosphate, pH 7.2, 150 mM NaCl) and then flash frozen in liquid nitrogen. X-ray diffraction data sets were collected at 100 K at beamline X06SA of the Swiss Light Source, Villigen, Switzerland. Diffraction data were integrated with the program XDS[6] and scaled with AIMLESS,[7] which is part of the CCP4 package.[8] The structures were then solved by difference Fourier analysis using PHENIX[9] with PDB entry 6SHZ as a starting model. Structure refinement was performed using iterative cycles of manual model building in COOT[10] and refinement in PHENIX. Dictionary files for compounds **21** and **22** were generated using the Grade Web Server

(http://grade.globalphasing.org). In both complexes, there was significant negative difference electron density at the iodine facing subsite 2 and positive difference density protruding from the iodine position toward the solvent after refinement, as observed previously for similar compounds with the Y220C mutant, suggesting a partial, radiation-induced loss of the iodine atom.[11-12] Data collection and refinement statistics are listed in **Table S1**. Structural figures in this paper were prepared using PyMOL (www.pymol.org).

Differential Scanning Fluorimetry (DSF)

Protein thermal stabilization was determined using SYPRO orange (Life Technologies) as a dye that increases in fluorescence quantitatively upon binding to hydrophobic protein surfaces exposed upon thermal denaturation. Real-time melt analysis was recorded on a Bio-rad CFX Connect Real-time qPCR system. DSF measurements were performed using 8 µM protein and 10x SYPRO orange in assay buffer (25 mM KPi, 150 mM NaCl, 1 mM TCEP, pH 7.2) at a final DMSO concentration of 5% (v/v). ΔT_m values were calculated as $\Delta T_m = T_m$ (protein + compound) − T_m (protein). ΔT_m values are reported as an average of three independent measurements.

Isothermal Titration Calorimetry (ITC)

ITC experiments were conducted using a MicroCal iTC200 calorimeter. For forward titrations, the cell unit contained 25-50 µM protein in freshly dialyzed assay buffer (25 mM KPi, 150 mM NaCl, 1 mM TCEP, pH 7.2) with a final DMSO content of 5% (v/v), and the syringe contained 0.5-5 mM compound in the same buffer. For reverse titrations, the cell unit contained 4-15 µM compound in freshly dialyzed assay buffer with final concentration 5% (v/v) DMSO, and the syringe contained 50-150 µM protein in the same buffer. Injection steps used were 2 µL (initial injection: 0.5 µL) at a rate of 2 µL/s with 120 s or 240 s spacing, and all experiments were performed at 25-30 °C. Data were analyzed using MicroCal PEAQ-ITC analysis software.

Synthetic Chemistry

Chemicals and solvents were bought from commercial suppliers and used as supplied. Reactions were monitored by thin layer chromatography (TLC) or liquid chromatography-mass spectrometry (LC-MS)

analysis. Products were purified by flash column chromatography on silica gel (60 Å pore size, 35-60 mesh particle size) or using a Biotage Isolera One with Biotage Sfär silica D columns. TLC was performed using aluminium TLC plates, silica gel 60 coated with fluorescent indicator F254 (Merck). TLC plates were visualized using UV light (254 nm) and/or by staining with potassium permanganate followed by heating. Reactions were performed using anhydrous solvents under argon after three cycles of evacuation and purging unless otherwise indicated. Solvents were removed by rotary evaporator below 40˚C, and the compounds were further dried using high vacuum pumps.

^{1}H, ^{13}C and ^{19}F NMR spectra were recorded on a Bruker Advance III HD FT-NMR spectrometer equipped with an Ascend™ 400 magnet at 400 MHz, 101 MHz, and 376 MHz, respectively. Chemical shifts (δ) are quoted in ppm (parts per million) and referenced to solvent signals: ^{1}H δ = 7.26 ($CDCl_3$), 2.50 ($(CD_3)_2SO$), 3.31 (CD_3OD), 2.05 ($(CD_3)_2CO$); ^{13}C δ = 77.16 ($CDCl_3$), 39.52 ($(CD_3)_2SO$), 49.00 (CD_3OD), 206.26 ($(CD_3)_2CO$). ^{19}F NMR spectra were referenced externally to $CFCl_3$. Coupling constants (*J*) are given in Hz.

High-resolution mass spectra were recorded using positive/negative ion electrospray ionisation on a Bruker SolariX FT-ICR mass spectrometer equipped with a 4.7 T superconducting magnet. Infrared spectra were recorded using a Thermo Scientific Nicolet iS5 FT-IR (ATR) spectrometer with an iD7 ATR accessory. Melting points were recorded using an Electrothermal IA9300 melting point apparatus.

GENERAL PROCEDURES

General procedure 1. Diiodination of salicylic acid derivatives

The appropriate salicylic acid derivative was dissolved in AcOH (0.3 M) at room temperature and NIS (2.05 eq.) was added. The reaction was stirred until completion as monitored by LCMS. Upon completion, sat. aq. $Na_2S_2O_3$ was added (0.5 mL per 50 mL solvent) and the reaction concentrated *in vacuo*. The residue was suspended in 1M HCl (aq.), then filtered and the precipitate re-suspended and washed with 1M HCl on the filter (2x). The solid was collected and dried under high vacuum.

General procedure 2. S_NAr coupling with aryl fluoride intermediate 9

4-fluoro-2-hydroxy-3,5-diiodobenzoic acid **9** (1 eq.) was dissolved in DMSO (0.15 M), then the nucleophile (3 eq.) and Cs_2CO_3 (6 eq.) were added and the reaction heated at 150 °C for 2 hours. The solvent was removed by vacuum distillation (*ca*. 80 °C) and the residue washed with 1M HCl and extracted with 4:1 $CHCl_3$:*i*PrOH (3x). The combined organic layers were dried ($MgSO_4$), concentrated, and then the product purified by flash column chromatography.

General procedure 3. S_NAr coupling of heterocycles with protected aryl fluoride intermediate 10

Methyl 4-fluoro-3,5-diiodo-2-methoxybenzoate **10** (1 eq.), the appropriate heterocycle (1.5 eq.) and Cs_2CO_3 (3 eq.) were dissolved in DMSO (0.15 M) and the reaction heated at 70 °C for 1 hour. The reaction was added to sat. aq. $NaHCO_3$ and extracted with Et_2O (3x). The combined organic layers were washed with water (1x), sat. aq. NaCl (1x), dried ($MgSO_4$) and concentrated *in vacuo*. The product was purified by flash column chromatography.

General procedure 4. Methoxy deprotection of methyl 2-methoxybenzoate derivatives

The appropriate methyl 2-methoxybenzoate derivative was dissolved in CH_2Cl_2 (0.1M) at 0 °C, then BBr_3 (1M, CH_2Cl_2) (3 eq.) was added slowly. The reaction was stirred at 0 °C for 0.5 hours, then warmed to room temperature and stirred for a further 24 hours. The reaction was quenched by careful addition of MeOH, then washed with H_2O and extracted with 4:1 $CHCl_3$:*i*PrOH. The combined organic layers were dried ($MgSO_4$), then concentrated *in vacuo* and purified by flash column chromatography.

General procedure 5. Paal-Knorr pyrrole synthesis

Methyl 4-amino-3,5-diiodo-2-methoxybenzoate **38** (1 eq.), the appropriate dicarbonyl (1.1 eq.) and 37% HCl (aq.) (0.4 mL per 50 mL EtOH) were dissolved in EtOH (0.1 M) and heated to reflux for 15-18 hours. The reaction was quenched by dropwise addition of solid $NaHCO_3$, then the solvent was removed *in vacuo*. The residue was washed with sat. aq. $NaHCO_3$ and extracted with CH_2Cl_2 (3x). The combined organic layers were dried ($MgSO_4$), concentrated *in vacuo*, and the product was purified by flash column chromatography.

General procedure 6. Desilylation and S_NAr coupling of substituted 1-(triisopropylsilyl)pyrroles with aryl fluoride intermediate 10

Methyl 4-fluoro-3,5-diiodo-2-methoxybenzoate **10** (1 eq.), the appropriate 1-(triisopropylsilyl)pyrrole derivative (1.5 eq.), KF (3 eq.) and Cs_2CO_3 (3 eq.) were dissolved in DMSO (0.15 M) and the reaction heated at 70 °C for 1 hour. The reaction was added to sat. aq. $NaHCO_3$ and extracted with Et_2O (3x). The combined organic layers were washed with water (1x), sat. aq. NaCl (1x), dried ($MgSO_4$) and concentrated *in vacuo*. The product was purified by flash column chromatography.

General procedure 7. *O*-alkylation of phenol intermediate 57

The appropriate alkyl iodide (1.5 eq.) was added to a 25 °C solution of 7-hydroxy-6,8-diiodo-2,2-dimethyl-4*H*-benzo[*d*][1,3]dioxin-4-one **57** (1 eq.) and Cs_2CO_3 (3 eq.) in DMF (0.2 M) and the reaction stirred until completion, monitored by LCMS. Upon completion, the reaction was diluted with sat. aq. NH_4Cl and extracted with Et_2O (3x). The combined organic layers were dried ($MgSO_4$) and concentrated *in vacuo.* The product was purified by flash column chromatography.

General procedure 8. Mitsunobu alkylation of phenol intermediate 57

Triphenylphosphine (1 eq.) was added at 0 °C to a solution of 7-hydroxy-6,8-diiodo-2,2-dimethyl-4*H*-benzo[*d*][1,3]dioxin-4-one **57** (1 eq.), diisopropylazodicarboxylate (1 eq.) and the appropriate alcohol (1 eq.) in THF (0.1 M). After 30 minutes, the reaction was warmed to room temperature and stirred a further 2 hours. Upon completion, the reaction was concentrated *in vacuo*, and the product was purified by flash column chromatography.

General procedure 9. Acetonide hydrolysis

The acetonide was dissolved in a 1:1 mixture of THF:1M NaOH (aq.) (0.05 M) at room temperature and stirred until completion as monitored by TLC. The reaction was diluted with water, then acidified to pH <4 with 1M HCl (aq.) and extracted with EtOAc (3x). The combined organic layers were washed with sat. aq. NaCl, dried ($MgSO_4$), and concentrated *in vacuo.* The product was purified by flash column chromatography.

General procedure 10. *N*-alkylation of aniline intermediate 38

The appropriate alkyl iodide (1.1 eq. monoiodide, 1.5 eq. diiodide) was added to a solution of methyl 4-amino-3,5-diiodo-2-methoxybenzoate **38** (1 eq.) and Cs_2CO_3 (3 eq.) in DMF (0.1 M), and the reaction heated at 80 °C for 2-4 hours. The reaction was diluted with sat. aq. $NaHCO_3$ and extracted with Et_2O (3x). The combined organic layers were washed with sat. aq. NaCl, dried ($MgSO_4$) and concentrated *in vacuo*, and the product purified by flash column chromatography.

General procedure 11. Demethylation of methyl 2-methoxybenzoate derivatives

The appropriate methyl 2-methoxybenzoate derivative was dissolved in CH_2Cl_2 (0.1 M) at 0 °C, then BCl_3 (1M, CH_2Cl_2) (3.5 eq.) was added. The reaction was stirred at 0 °C for 5 minutes, then warmed to room temperature and stirred for 30 minutes. To the reaction was added sat. aq. $NaHCO_3$, then the mixture was extracted with CH_2Cl_2 (3x). The combined organic layers were concentrated *in vacuo.* The crude material was dissolved in a 1:1:1 mixture of THF:MeOH:1M NaOH (aq.) (0.03 M) and stirred until completion as monitored by LCMS. The reaction was diluted with water, acidified to pH <3 with 2M

HCl (aq.) and extracted with 4:1 $CHCl_3$:*i*PrOH (3x). The combined organic layers were dried ($MgSO_4$), concentrated *in vacuo,* and the product was purified by flash column chromatography.

General procedure 12. Direct amidation of ester derivatives

The appropriate ester derivative (1 eq.) was stirred in MeOH (0.2 M) and THF added until full dissolution (if required). Ethylamine (2M, MeOH) (40 eq.) was added and the reaction stirred at room temperature until completion as monitored by LCMS. The reaction was concentrated *in vacuo*, and the product was purified by flash column chromatography.

N-HETEROCYCLIC SERIES

4-fluoro-2-hydroxy-3,5-diiodobenzoic acid (9)

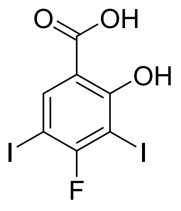

4-fluoro-2-hydroxy-3,5-diiodobenzoic acid **9** was prepared according to general procedure 1; pink solid, (9.37 g, 23.0 mmol, 86%); TLC R*f* = 0.20 (10% MeOH in CH_2Cl_2); m.p. 222-223 °C (decomposition); FT-IR (ATR) ν_{max}/cm^{-1} 3273, 1618, 1551, 1424, 1369, 1262; ^{1}H NMR δ_H (400 MHz, $(CD_3)_2SO$) 7.99 (d, *J* = 8.3 Hz, 1H); ^{13}C NMR δ_C (101 MHz, $(CD_3)_2SO$) 168.6, 167.0 (d, *J* = 7.3 Hz), 162.0 (d, *J* = 240.6 Hz), 139.1 (d, *J* = 5.1 Hz), 116.5, 75.2 (d, *J* = 27.1 Hz), 62.3 (d, *J* = 30.8 Hz); ^{19}F NMR δ_F (376 MHz, $(CD_3)_2SO$) -69.4 (s, 1F); *m/z* (ESI-) calc'd for $C_7H_2FI_2O_3$ $[M-H]^-$ 406.8083, found: 406.8080.

Methyl 4-fluoro-3,5-diiodo-2-methoxybenzoate (10)

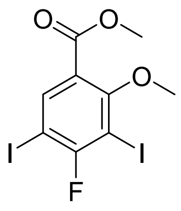

Dimethyl sulfate (0.57 mL, 6.01 mmol) was added to a solution of 4-fluoro-2-hydroxy-3,5-diiodobenzoic acid **9** (815 mg, 2.00 mmol) and K_2CO_3 (1.13 g, 8.18 mmol) in *N*-methylpyrrolidinone (8 mL) at room temperature. The reaction was stirred for 10 minutes, then heated to 80 °C for 1 hour. The reaction was cooled, added to Et_2O (150 mL) and washed with sat. aq. $NaHCO_3$ (2 x 150 mL) then sat. aq. NaCl (150 mL). The organic phase was dried ($MgSO_4$) and concentrated *in vacuo*, and the product was purified by flash column chromatography (25% CH_2Cl_2 in hexane) yielding methyl 4-fluoro-3,5-diiodo-2-methoxybenzoate **10** as a white solid (592 mg, 1.36 mmol, 68%); TLC R*f* = 0.30 (25% CH_2Cl_2 in hexane); m.p. 107 °C; FT-IR (ATR) ν_{max}/cm^{-1} 3390 br, 2950, 1690, 1521, 1280, 1240; ^{1}H NMR δ_H (400 MHz, $CDCl_3$) 8.26 (d, *J* = 7.2 Hz, 1H), 3.92 (s, 3H), 3.90 (s, 3H); ^{13}C NMR δ_C (101 MHz, $CDCl_3$) 163.7 (d, *J* = 249.4 Hz), 163.6, 162.2 (d, *J* = 4.4 Hz), 142.1 (d, *J* = 3.7 Hz), 122.9 (d, *J* = 3.7 Hz), 82.6 (d, *J* = 27.9 Hz), 74.2 (d, *J* = 30.1 Hz), 62.8, 52.9; ^{19}F NMR δ_F (376 MHz, $CDCl_3$) -61.31 (d, *J* = 6.9 Hz, 1F); *m/z* (ESI+) calc'd for $C_9H_7FI_2NaO_3$ $[M+Na]^+$ 458.8361, found: 458.8368.

2-hydroxy-3,5-diiodo-4-(1*H*-pyrazol-1-yl)-benzoic acid (17)

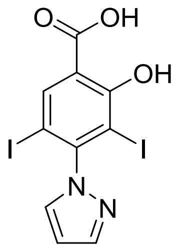

2-hydroxy-3,5-diiodo-4-(1*H*-pyrazol-1-yl)-benzoic acid **17** was synthesized according to general procedure 2; beige solid (272 mg, 0.60 mmol, 58%); TLC R*f* = 0.15 (10% MeOH in CH_2Cl_2 + 1% AcOH); m.p. 241 °C (decomposition); FT-IR (ATR) ν_{max}/cm^{-1} 3450 br, 2973, 1683, 1551, 1396; ^{1}H NMR δ_H (400 MHz, $(CD_3)_2SO$) 8.10 (s, 1H), 7.76 (dd, J_1 = 2.5 Hz, J_2 = 0.6 Hz, 1H), 7.67 (dd, J_1 = 1.8 Hz, J_2 = 0.6 Hz, 1H), 6.46 (dd, J_1 = 2.5 Hz, J_2 = 1.8 Hz, 1H); ^{13}C NMR δ_C (101 MHz, $(CD_3)_2SO$) 168.2, 165.8, 147.1, 139.8, 138.7, 131.0, 120.8, 106.3, 92.8, 78.1; *m/z* (ESI+) calc'd for $C_{10}H_7I_2N_2O_3$ $[M+H]^+$ 456.8541, found: 456.8548.

2-hydroxy-4-(1*H*-indol-1-yl)-3,5-diiodobenzoic acid (19)

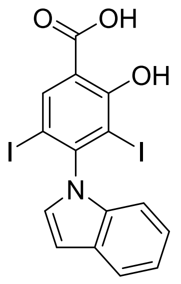

2-hydroxy-4-(indol-1-yl)-3,5-diiodobenzoic acid **19** was synthesized according to general procedure 2; red solid (662 mg, 1.31 mmol, 55%); TLC R*f* = 0.30 (15% MeOH in CH_2Cl_2); m.p. 183-184 °C (decomposition); FT-IR (ATR) ν_{max}/cm^{-1} 3396, 2973, 1669, 1551, 1527; ^{1}H NMR δ_H (400 MHz, $(CD_3)_2SO$) 8.20 (s, 1H), 7.61 (m, 1H), 7.22 (d, J = 3.3 Hz, 1H), 7.09 (m, 2H), 6.80 (m, 1H), 6.65 (dd, J_1 = 3.3 Hz, J_2 = 0.8 Hz, 1H); ^{13}C NMR δ_C (100 MHz, $(CD_3)_2SO$) 168.5, 165.6, 145.3, 139.1, 134.7, 128.0, 128.0, 122.0, 120.7, 120.6, 119.6, 110.3, 102.9, 93.5, 79.9; *m/z* (ESI+) calc'd for $C_{15}H_{10}I_2NO_3$ $[M+H]^+$ 505.8745, found: 505.8746.

Methyl 4-(1*H*-imidazol-1-yl)-3,5-diiodo-2-methoxybenzoate (12)

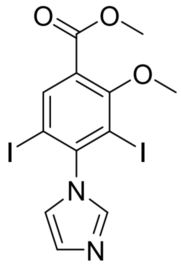

Methyl 4-(1*H*-imidazol-1-yl)-3,5-diiodo-2-methoxybenzoate **12** was prepared according to general procedure 3; white solid (157 mg, 0.32 mmol, 68%); TLC R*f* = 0.55 (5% MeOH in CH_2Cl_2); m.p. 168 °C; FT-IR (ATR) ν_{max}/cm^{-1} 3109, 2947, 1699, 1227; ^1H NMR δ_H (400 MHz, $(CD_3)_2SO$) 8.34 (s, 1H), 7.53 (br. s, 1H), 7.30 (br. s, 1H), 6.92 (br. s, 1H), 3.97 (s, 3H), 3.94 (s, 3H); ^{13}C NMR δ_C (101 MHz, $(CD_3)_2SO$) 163.6, 161.0, 146.6, 141.7, 136.2, 130.0, 127.6, 119.1, 99.5, 90.3, 62.8, 53.2; *m/z* (ESI+) calc'd for $C_{12}H_{11}I_2N_2O_3$ $[M+H]^+$ 484.8854, found: 484.8855.

2-hydroxy-4-(1*H*-imidazol-1-yl)-3,5-diiodobenzoic acid (18)

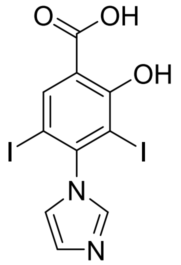

2-hydroxy-4-(1*H*-imidazol-1-yl)-3,5-diiodobenzoic acid **18** was synthesized according to general procedure 4; white solid (29.2 mg, 64.0 µmol, 58%); TLC R*f* = 0.30 (20% MeOH in CH_2Cl_2 + 0.5% AcOH); m.p. 238 °C (decomposition); FT-IR (ATR) ν_{max}/cm^{-1} 3410 br, 2973, 1669, 1552, 1526; ^1H NMR δ_H (400 MHz, $(CD_3)_2SO$) 8.09 (s, 1H), 7.62 (s, 1H), 7.15-7.03 (m, 2H); ^{13}C NMR δ_C (101 MHz, $(CD_3)_2SO$) 168.2, 165.9, 144.1, 138.7, 136.6, 128.7, 120.7, 119.6, 92.8, 78.0; *m/z* (ESI+) calc'd for $C_{10}H_7I_2N_2O_3$ $[M+H]^+$ 456.8541, found: 456.8549.

Methyl 3,5-diiodo-2-methoxy-4-(1*H*-pyrrol-1-yl)benzoate (11)

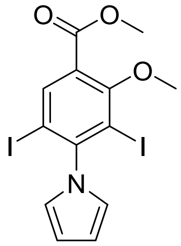

Methyl 3,5-diiodo-2-methoxy-4-(1*H*-pyrrol-1-yl)benzoate **11** was prepared according to general procedure 3; white solid (117 mg, 0.24 mmol, 45%); TLC R*f* = 0.40 (50% CH_2Cl_2 in hexane); m.p. 129-130 °C; FT-IR (ATR) ν_{max}/cm^{-1} 2942, 1710, 1227; ^1H NMR δ_H (400 MHz, $(CD_3)_2SO$) 8.32 (s, 1H), 6.60 (t, *J* = 2.1 Hz, 2H), 6.40 (t, *J* = 2.1 Hz, 2H), 3.97 (s, 3H), 3.93 (s, 3H); ^{13}C NMR δ_C (101 MHz, $(CD_3)_2SO$) 163.8, 160.8, 150.6, 141.5, 126.7, 120.5, 110.1, 100.1, 91.2, 62.7, 53.1; *m/z* (ESI+) calc'd for $C_{13}H_{12}I_2NO_3$ $[M+H]^+$ 483.8901, found: 483.8899.

2-hydroxy-3,5-diiodo-4-(1*H*-pyrrol-1-yl)benzoic acid (4)

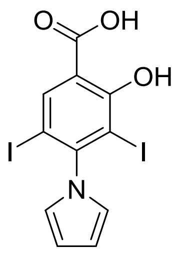

2-hydroxy-3,5-diiodo-4-(1*H*-pyrrol-1-yl)benzoic acid **4** was prepared according to general procedure 4; grey solid (36.8 mg, 80.9 μmol, 27%); TLC R*f* = 0.50 (20% MeOH in CH_2Cl_2); m.p. 199-200 °C (decomposition); FT-IR (ATR) ν_{max}/cm^{-1} 1558, 1416, 1360, 1245; ^1H NMR δ_H (400 MHz, $(CD_3)_2SO$) 11.42 (s, 1H), 8.09 (s, 1H), 6.60 (t, *J* = 2.0 Hz, 2H), 6.18 (t, *J* = 2.0 Hz, 2H); ^{13}C NMR δ_C (101 MHz, $(CD_3)_2SO$) 168.3, 165.5, 147.7, 138.7, 120.6, 120.3, 108.8, 92.8, 78.8; *m/z* (ESI+) calc'd for $C_{11}H_8I_2NO_3$ $[M+H]^+$ 455.8588, found: 455.8586.

ALKYLPYRROLE SERIES

4-amino-3,5-diiodo-2-hydroxybenzoic acid (39)

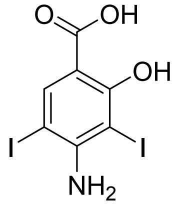

4-amino-2-hydroxybenzoic acid (2.53 g, 16.5 mmol) was dissolved in MeCN (50 mL) at 0 °C, then *N*-iodosuccinimide (7.81 g, 34.7 mmol) added in portions. After 30 minutes, 2M aq. $Na_2S_2O_3$ (1 mL) was added and the reaction concentrated *in vacuo*. The crude solid was suspended in H_2O (50 mL), filtered,

then the filter washed with H_2O (50 mL) and the precipitate dried under high vacuum yielding 4-amino-3,5-diiodo-2-hydroxybenzoic acid **39** as a black solid (6.68 g, 16.5 mmol, quant.), which was used without further purification; TLC R*f* = 0.30 (15% MeOH in CH_2Cl_2); m.p. 129-130 °C (decomposition); FT-IR (ATR) ν_{max}/cm^{-1} 3312, 3100 br., 1597; ^1H NMR δ_H (400 MHz, $(CD_3)_2SO$) 12.44 (br s, 1H), 7.99 (s, 1H), 5.89 (br s, 2H); ^{13}C NMR δ_C (101 MHz, $(CD_3)_2SO$) 170.6, 161.9, 152.8, 139.8, 103.9, 70.6, 69.1; *m/z* (ESI-) calc'd for $C_7H_4I_2NO_3$ $[M-H]^-$ 403.8286, found: 403.8274.

Methyl 4-amino-3,5-diiodo-2-methoxybenzoate (38)

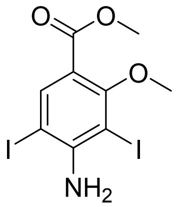

Methyl 4-amino-2-methoxybenzoate (5.43 g, 30.0 mmol) was dissolved in AcOH (190 mL), then *N*-iodosuccinimide (14.2 g, 63.0 mmol) added in portions. After 1 hour, the reaction was concentrated and the residue washed with a mixture of sat. aq. $NaHCO_3$ (400 mL) and 1M NaOH (100 mL), then extracted with EtOAc (2 x 500 mL). The organic phases were washed with sat. aq. NaCl (500 mL), dried ($MgSO_4$), then concentrated *in vacuo* and dried under high vacuum yielding methyl 4-amino-3,5-diiodo-2-methoxybenzoate **38** as a yellow solid, which was used without further purification (12.8 g, 29.5 mmol, 98%); TLC R*f* = 0.30 (5% EtOAc in petroleum ether); m.p. 127 °C; FT-IR (ATR) ν_{max}/cm^{-1} 3399, 3300, 2944, 1715, 1601, 1224; ^1H NMR δ_H (400 MHz, $CDCl_3$) 8.26 (s, 1H), 5.15 (br s, 2H), 3.87 (s, 3H), 3.85 (s, 3H); ^{13}C NMR δ_C (101 MHz, $CDCl_3$) 163.9, 161.7, 151.4, 142.5, 114.8, 81.6, 74.2, 62.1, 52.2; *m/z* (ESI+) calc'd for $C_9H_9I_2NNaO_3$ $[M+Na]^+$ 455.8564, found: 455.8570.

Methyl 4-(2,5-dimethyl-1*H*-pyrrol-1-yl)-3,5-diiodo-2-methoxybenzoate (41)

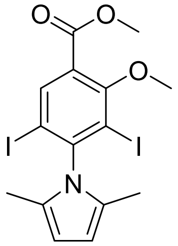

Methyl 4-(2,5-dimethyl-1*H*-pyrrol-1-yl)-3,5-diiodo-2-methoxybenzoate **41** was prepared according to general procedure 5; yellow solid (563 mg, 1.10 mmol, 22%); TLC R*f* = 0.25 (5% EtOAc in hexane); m.p.

163-164 °C; FT-IR (ATR) ν_{max}/cm^{-1} 2930, 1774, 1731, 1433, 1354, 1225; ^{1}H NMR δ_H (400 MHz, $CDCl_3$) 8.34 (s, 1H), 5.98 (s, 2H), 3.98 (s, 3H), 3.94 (s, 3H), 1.95 (s, 6H); ^{13}C NMR δ_C (101 MHz, $CDCl_3$) 163.9, 160.9, 149.0, 141.3, 126.7, 126.4 , 107.0 , 101.6, 93.2, 62.8, 53.1, 12.8 ; *m/z* (ESI+) calc'd for $C_{15}H_{16}I_2NO_3$ $[M+H]^+$ 511.9214, found: 511.9221.

4-oxopentanal (54)

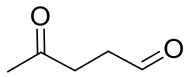

5-hydroxy-2-pentanone (599 mg, 5.87 mmol) and triethylamine (4.00 mL, 28.7 mmol) were dissolved in CH_2Cl_2 (20 mL) at 0 °C, then a solution of SO_3•pyridine (4.85 g, 30.5 mmol) in DMSO (20 mL) added. After 15 hours, the reaction was diluted with water (200 mL) and extracted with Et_2O (2 x 200 mL). The combined organic layers were washed with water (2 x 100 mL), sat. aq. NaCl (100 mL), dried ($MgSO_4$) and concentrated *in vacuo* yielding crude 4-oxopentanal **54** as a yellow oil that was used without further purification (1.07 g, crude); TLC R*f* = 0.50 (10% EtOAc in CH_2Cl_2); FT-IR (ATR) ν_{max}/cm^{-1} 2921, 1713, 1482; ^{1}H NMR δ_H (400 MHz, $CDCl_3$) 9.79 (s, 1H), 2.74 (m, 4H), 2.20 (s, 3H); ^{13}C NMR δ_C (101 MHz, $CDCl_3$) 206.5, 200.6, 37.6, 35.6, 29.9.

Methyl 4-(2-methyl-1*H*-pyrrol-1-yl)-3,5-diiodo-2-methoxybenzoate (40)

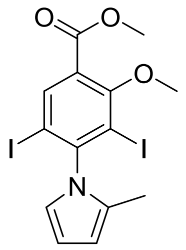

Methyl 4-(2-methyl-1*H*-pyrrol-1-yl)-3,5-diiodo-2-methoxybenzoate **40** was synthesized according to general procedure 5; pale yellow solid (1.28 g, 2.57 mmol, 35% over 2 steps); TLC R*f* = 0.30 (10% EtOAc in hexane); m.p. 115-116 °C; FT-IR (ATR) ν_{max}/cm^{-1} 3396, 3130, 1634, 1412; ^{1}H NMR δ_H (400 MHz, $CDCl_3$) 8.32 (s, 1H), 6.45 (dd, J_1 = 3.0 Hz, J_2 = 1.7 Hz, 1H), 6.29 (t, J = 3.0 Hz, 1H), 6.09 (m, 1H), 3.97 (s, 3H), 3.94 (s, 1H), 2.01 (s, 3H); ^{13}C NMR δ_C (101 MHz, $CDCl_3$) 163.9, 160.8, 149.8, 141.3, 127.9, 126.8, 118.6, 109.5, 108.1, 100.9, 92.3, 62.8, 53.1, 12.4; *m/z* (ESI+) calc'd for $C_{14}H_{14}I_2NO_3$ $[M+H]^+$ 497.9058, found: 497.9063.

Methyl 4-(3-acetyl-1*H*-pyrrol-1-yl)-3,5-diiodo-2-methoxybenzoate (42)

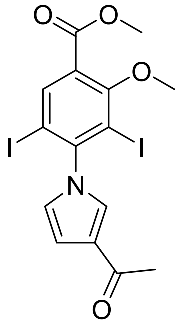

To a 0 °C solution of methyl 3,5-diiodo-2-methoxy-4-(1*H*-pyrrol-1-yl)benzoate **11** (2.47 g, 5.11 mmol) in CH_2Cl_2 (35 mL) was added Ac_2O (0.73 mL, 7.72 mmol) followed by BF_3•OEt_2 (1.90 mL, 15.4 mmol). The reaction was stirred at 0 °C for 1 hour, then at room temperature for a further 1 hour. The reaction was quench by dropwise addition of sat. aq. $NaHCO_3$ (5 mL) and stirred for 5 minutes, then diluted with sat. aq. $NaHCO_3$ (30 mL) and extracted with CH_2Cl_2 (3 x 30 mL). The combined organic layers were dried ($MgSO_4$) and concentrated *in vacuo.* Purification by flash column chromatography (20% EtOAc in petroleum ether) yielded methyl 4-(3-acetyl-1*H*-pyrrol-1-yl)-3,5-diiodo-2-methoxybenzoate **42** as a yellow solid (1.53 g, 2.92 mmol, 57%); TLC R*f* = 0.20 (20% EtOAc in petroleum ether); m.p. 48-49 °C; FT-IR (ATR) ν_{max}/cm^{-1} 3446 br, 2935, 1730, 1653, 1529, 1270, 1220; ^1H NMR δ_H (400 MHz, $CDCl_3$) 8.33 (s, 1H), 7.21 (t, *J* = 1.8 Hz, 1H), 6.82 (dd, J_1 = 3.0 Hz, J_2 = 1.8 Hz, 1H), 6.57 (dd, J_1 = 3.0 Hz, J_2 = 1.8 Hz, 1H) 3.97 (s, 3H), 3.93 (s, 3H), 2.47 (s, 3H); ^{13}C NMR δ_C (101 MHz, $CDCl_3$) 193.5, 163.6, 160.9, 149.2, 141.7, 127.8, 127.3, 125.8, 122.5, 110.6, 99.5, 90.3, 62.8, 53.2, 27.5; *m/z* (ESI+) calc'd for $C_{15}H_{14}I_2NO_4$ $[M+H]^+$ 525.9007, found: 525.9015.

3-methyltetrahydrofuran-2-ol (49)

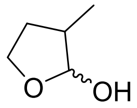

DIBAL (1M, hexane) (6.00 mL, 6.00 mmol) was added at -78 °C to a solution of α-methyl-γ-butyrolactone (560 mg, 5.60 mmol) in Et_2O (30 mL). The reaction was stirred for 30 minutes, then warmed to room temperature and quenched with sat. aq. Rochelle's salt (10 mL). The reaction was washed with sat. aq. NH_4Cl (20 mL) and extracted with Et_2O (2 x 30 mL), then dried ($MgSO_4$) and concentrated *in vacuo*. The product was purified by flash column chromatography (10% acetone in CH_2Cl_2) yielding 3-methyltetrahydrofuran-2-ol **49** (mix of diastereoisomers) as a colorless oil (462 mg, 4.52 mmol, 81%); TLC R*f* = 0.40 (10% acetone in CH_2Cl_2); FT-IR (ATR) ν_{max}/cm^{-1} 3384 br., 2963, 2878; ^1H

NMR δ_H (400 MHz, $CDCl_3$) 5.26 (d, *J* = 4.5 Hz, 0.5H), 5.09 (d, *J* = 1.2 Hz, 1H), 4.05 (m, 1.5H), 3.94 (m, 1H), 3.80 (m, 0.5H), 3.17 (m, 1.5H), 2.20 (m, 2H), 2.11 (m, 0.5H), 1.97 (m, 0.5H), 1.74 (m, 0.5H), 1.52 (m, 1H), 1.09 (d, *J* = 6.9 Hz, 1.5H), 1.02 (d, *J* = 7.1 Hz, 3H); ^{13}C NMR δ_C (101 MHz, $CDCl_3$) 104.4, 99.3, 67.4, 67.0, 40.5, 38.7, 31.7, 30.8, 17.3, 12.9; *m/z* (ESI+) calc'd for $C_5H_{10}NaO_2$ $[M+Na]^+$ 125.0573, found: 125.0571.

3-(5,5-dimethyl-1,3-dioxan-2-yl)butan-1-ol (50) and 2,2-dimethyl-3-((3-methyltetrahydrofuran-2-yl)oxy)propan-1-ol (51)

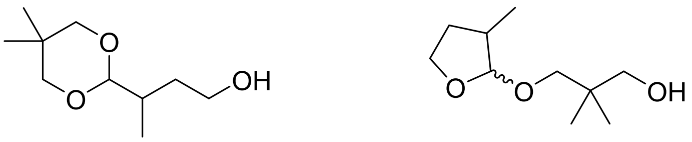

3-methyltetrahydrofuran-2-ol **49** (1.71 g, 16.7 mmol), 2,2-dimethylpropane-1,3-diol (17.5 g, 168 mmol) and TsOH•H_2O (303 mg, 1.59 mmol) were added to toluene (100 mL) and heated to 100 °C for 2 hours. The reaction was cooled, washed with sat. aq. $NaHCO_3$ (100 mL), then extracted with CH_2Cl_2 (3 x 100 mL). Then organic phases were dried ($MgSO_4$), concentrated, then purified by flash column chromatography (10% acetone in CH_2Cl_2) yielding 3-(5,5-dimethyl-1,3-dioxan-2-yl)butan-1-ol **50** (1.44 g, 7.64 mmol, 46%) and 2,2-dimethyl-3-((3-methyltetrahydrofuran-2-yl)oxy)propan-1-ol **51** (mix of diastereoisomers) (891 mg, 4.73 mmol, 28%) as colorless oils.

Recycling of by-product:

2,2-dimethyl-3-((3-methyltetrahydrofuran-2-yl)oxy)propan-1-ol **51** (189 mg, 1.01 mmol), 2,2-dimethylpropane-1,3-diol (1.05 g, 10.1 mmol) and TsOH•H_2O (19.7 mg, 0.10 mmol) were added to toluene (10 mL) and heated to 100 °C for 2 hours. The reaction was cooled, washed with sat. aq. $NaHCO_3$ (20 mL), then extracted with CH_2Cl_2 (3 x 20 mL). Then organic phases were dried ($MgSO_4$), concentrated, then purified by flash column chromatography (10% acetone in CH_2Cl_2) yielding 3-(5,5-dimethyl-1,3-dioxan-2-yl)butan-1-ol **50** (94.5 mg, 0.50 mmol, 50%) and 2,2-dimethyl-3-((3-methyltetrahydrofuran-2-yl)oxy)propan-1-ol **51** (mix of diastereoisomers) (36.2 mg, 0.19 mmol, 19%) as colorless oils.

3-(5,5-dimethyl-1,3-dioxan-2-yl)butan-1-ol **50**; TLC R*f* = 0.30 (10% acetone in CH_2Cl_2); FT-IR (ATR) ν_{max}/cm^{-1} 3379, 2953, 2870, 1393; ^1H NMR δ_H (400 MHz, $CDCl_3$) 4.28 (d, *J* = 3.8 Hz, 1H), 3.74 (m, 1H), 3.62 (m, 3H), 3.42 (m, 2H), 2.29 (br s, 1H), 1.84 (m, 2H), 1.53 (m, 1H), 1.18 (s, 3H), 0.98 (d, *J* = 7.0 Hz, 3H), 0.71 (s, 3H); ^{13}C NMR δ_C (101 MHz, $CDCl_3$) 105.0, 61.2, 35.4, 34.6, 30.3, 23.1, 21.9, 15.3; *m/z* (ESI+) calc'd for $C_{10}H_{20}NaO_3$ $[M+Na]^+$ 211.1305, found: 211.1303.

2,2-dimethyl-3-((3-methyltetrahydrofuran-2-yl)oxy)propan-1-ol **51** (mix of diastereosiomers); TLC R*f* = 0.40 (10% acetone in CH_2Cl_2); FT-IR (ATR) ν_{max}/cm^{-1} 3421 br., 2961, 2875; ^1H NMR δ_H (400 MHz, $CDCl_3$) 4.80 (d, *J* = 4.7 Hz, 0.5H) 4.66 (d, *J* = 1.6 Hz, 1H), 3.92 (m, 3H), 3.57 (d, *J* = 9.3 Hz, 0.5H), 3.53 (d, *J* = 9.4 Hz, 1H), 3.44 (m, 1.5H), 3.36 (t, J = 10.1 Hz, 1.5H), 3.21 (d, *J* = 9.4 Hz, 1H), 3.17 (d, *J* = 9.4 Hz, 0.5H), 2.72 (m, 1.5H), 2.17 (m, 2.5H), 1.99 (m, 0.5H), 1.66 (m, 0.5H), 1.49 (m, 1.5H), 1.06 (d, *J* = 6.7 Hz, 1.5H), 1.01 (d, *J* = 7.0 Hz, 3H), 0.89 (m, 9H); ^{13}C NMR δ_C (101 MHz, $CDCl_3$) 110.2, 105.0, 75.7, 75.5, 71.4, 71.1, 67.2, 67.0, 39.7, 38.5, 36.3, 36.2, 31.9, 31.3, 22.0, 21.9 , 17.6, 13.0; *m/z* (ESI+) calc'd for $C_{10}H_{20}NaO_3$ $[M+Na]^+$ 211.1305, found: 211.1302.

3-(5,5-dimethyl-1,3-dioxan-2-yl)butanal (52)

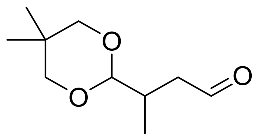

3-(5,5-dimethyl-1,3-dioxan-2-yl)butan-1-ol **50** (583 mg, 3.10 mmol) and triethylamine (2.20 mL, 15.8 mmol) were dissolved in CH_2Cl_2 (10 mL) at 0 °C, then a solution of SO_3•pyridine (2.52 g, 15.8 mmol) in DMSO (10 mL) added. After 15 hours, the reaction was added to sat. aq. NaCl (200 mL) and extracted with CH_2Cl_2 (3 x 200 mL). The organic layers were washed with sat. aq. NaCl (200 mL), dried ($MgSO_4$) and concentrated *in vacuo*, then purified by flash column chromatography (5% acetone in petroleum ether) yielding 3-(5,5-dimethyl-1,3-dioxan-2-yl)butanal **52** as a colorless oil (401 mg, 2.15 mmol, 69%); TLC R*f* = 0.35 (5% acetone in petroleum ether); FT-IR (ATR) ν_{max}/cm^{-1} 2956, 2847, 1724, 1107; ^1H NMR δ_H (400 MHz, $CDCl_3$) 9.74 (t, *J* = 2.1 Hz, 1H), 4.29 (d, *J* = 3.8 Hz, 1H), 3.58 (dt, J_1 = 11.4 Hz, J_2 = 2.3 Hz, 2H), 3.38 (m, 2H), 2.66 (ddd, J_1 = 16.1 Hz, J_2 = 5.8 Hz, J_3 = 2.5 Hz, 1H), 2.31 (m, 2H), 1.14 (s, 3H), 1.01 (d, *J* = 6.9 Hz, 3H), 0.70 (s, 3H); ^{13}C NMR δ_C (101 MHz, $CDCl_3$) 202.6, 103.8, 77.3, 77.2, 45.7, 33.2, 30.3, 23.0, 21.9, 15.3; *m/z* (ESI+) calc'd for $C_{10}H_{18}NaO_3$ $[M+Na]^+$ 209.1148, found: 209.1146.

2-hydroxy-3,5-diiodo-4-(3-methyl-1*H*-pyrrol-1-yl)benzoic acid (44)

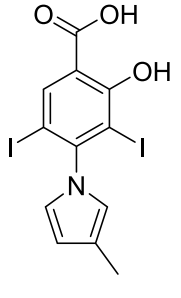

4-amino-2-hydroxy-3,5-diiodobenzoic acid (340 mg, 0.84 mmol) and 3-(5,5-dimethyl-1,3-dioxan-2-yl)butanal **52** (154 mg, 0.82 mmol) were dissolved in AcOH (8 mL) and heated to 100 °C. After 6 hours, the solvent was removed and the crude product purified by flash column chromatography (10% MeOH in CH_2Cl_2 + 0.5% AcOH) yielding 2-hydroxy-3,5-diiodo-4-(3-methyl-1*H*-pyrrol-1-yl)benzoic acid **44** as a brown solid (102 mg, 0.22 mmol, 26%); TLC R*f* = 0.25 (10% MeOH in CH_2Cl_2 + 0.5% AcOH); m.p. 178 °C (decomposition); FT-IR (ATR) ν_{max}/cm^{-1} 3407, 2972, 1618, 1557, 1416, 1350, 1234; ^1H NMR δ_H (400 MHz, $(CD_3)_2SO$) 8.09 (s, 1H), 6.49-6.47 (m, 1H), 6.38-6.36 (m, 1H), 6.03-6.00 (m, 1H), 1.91 (s, 3H); ^{13}C NMR δ_C (101 MHz, $(CD_3)_2SO$) 172.1, 168.7, 164.8, 148.3, 138.8, 120.6, 118.3, 118.2, 110.4, 92.7, 80.1, 12.0; *m/z* (ESI+) calc'd for $C_{12}H_{10}I_2NO_3$ $[M+H]^+$ 469.8745, found: 469.8745.

4-(2,5-dimethylpyrrol-1-yl)-2-hydroxy-3,5-diiodobenzoic acid (45)

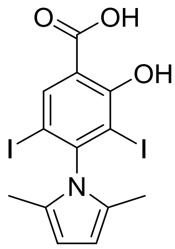

4-(2,5-dimethylpyrrol-1-yl)-2-hydroxy-3,5-diiodobenzoic acid **45** was synthesized according to general procedure 4; yellow solid (232 mg, 0.48 mmol, 50%); TLC R*f* = 0.15 (10% MeOH in CH_2Cl_2); m.p. 215 °C (decomposition); FT-IR (ATR) ν_{max}/cm^{-1} 2911, 1746, 1731, 1561, 1432, 1354, 1239; ^1H NMR δ_H (400 MHz, $(CD_3)_2SO$) 8.14 (s, 1H), 5.77 (s, 2H), 1.85 (s, 6H); ^{13}C NMR δ_C (101 MHz, $CDCl_3$) 168.4, 165.7, 145.4, 138.7, 125.2 , 120.5, 106.0 , 94.1, 80.5, 12.5 ; *m/z* (ESI+) calc'd for $C_{13}H_{12}I_2NO_3$ $[M+H]^+$ 483.8901, found: 483.8900.

2-hydroxy-3,5-diiodo-4-(2-methyl-1*H*-pyrrol-1-yl)benzoic acid (43)

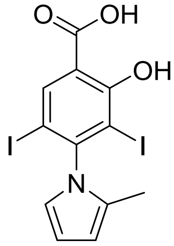

2-hydroxy-3,5-diiodo-4-(2-methyl-1*H*-pyrrol-1-yl)benzoic acid **43** was synthesized according to general procedure 4; brown solid (305 mg, 0.65 mmol, 27%); TLC R*f* = 0.25 (15% MeOH in CH_2Cl_2); m.p. 214 °C (decomposition); FT-IR (ATR) ν_{max}/cm^{-1} 2989, 2901, 1617, 1560, 1414; ^1H NMR δ_H (400 MHz,

$(CD_3)_2SO$) 8.09 (s, 1H), 6.43 (dd, J_1 = 3.0 Hz, J_2 = 1.7 Hz, 1H), 6.05 (t, J = 3.0 Hz, 1H), 5.91 (m, 1H), 1.90 (s, 3H); ^{13}C NMR δ_C (101 MHz, $(CD_3)_2SO$) 168.3, 165.5, 146.6, 138.7, 126.6, 120.5, 118.8, 108.3, 107.2, 93.5, 79.8, 12.1; *m/z* (ESI+) calc'd for $C_{12}H_{10}I_2NO_3$ $[M+H]^+$ 469.8745, found: 469.8745.

4-(3-acetyl-1*H*-pyrrol-1-yl)-2-hydroxy-3,5-diiodobenzoic acid (46)

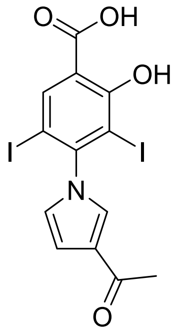

4-(3-acetyl-1*H*-pyrrol-1-yl)-2-hydroxy-3,5-diiodobenzoic acid **46** was synthesized according to general procedure 4; pink solid (764 mg, 1.54 mmol, 59%); TLC R*f* = 0.30 (20% MeOH in CH_2Cl_2); m.p. 235-236 °C (decomposition); FT-IR (ATR) ν_{max}/cm^{-1} 3372 br, 3110, 1634, 1563, 1410; ^1H NMR δ_H (400 MHz, $(CD_3)_2SO$) 8.12 (s, 1H), 7.58 (t, J = 1.8 Hz, 1H), 6.74 (dd, J_1 = 2.9 Hz, J_2 = 1.8 Hz, 1H), 6.59 (dd, J_1 = 2.9 Hz, J_2 = 1.8 Hz, 1H), 2.35 (s, 1H); ^{13}C NMR δ_C (101 MHz, $(CD_3)_2SO$) 192.4, 168.3, 165.5, 146.9, 138.8, 127.5, 126.5, 123.4, 120.1, 108.8, 92.6, 78.5, 27.0; *m/z* (ESI+) calc'd for $C_{13}H_{10}I_2NO_4$ $[M+H]^+$ 497.8694, found: 497.8703.

4-(3-ethyl-1*H*-pyrrol-1-yl)-2-hydroxy-3,5-diiodobenzoic acid (47)

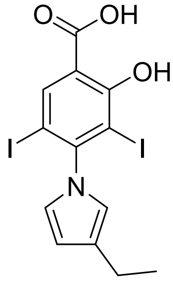

4-(3-acetyl-1*H*-pyrrol-1-yl)-2-hydroxy-3,5-diiodobenzoic acid **46** (275 mg, 0.55 mmol) and Et_3SiH (0.45 mL, 2.82 mmol) were dissolved in TFA (5.5 mL) and heated to 50 °C for 1 hour. The solvent was removed *in vacuo*, then the residue washed with 1M HCl (10 mL) and extracted with 4:1 $CHCl_3$:*i*PrOH (3 x 10 mL). The organic phases were combined, dried ($MgSO_4$), then concentrated *in vacuo* and purified by flash column chromatography (15% MeOH in CH_2Cl_2) yielding 4-(3-ethyl-1*H*-pyrrol-1-yl)-2-

hydroxy-3,5-diiodobenzoic acid **47** as a yellow solid (126 mg, 0.26 mmol, 48%); TLC R*f* = 0.25 (15% MeOH in CH_2Cl_2); m.p. 195-196 °C (decomposition); FT-IR (ATR) ν_{max}/cm^{-1} 3410 br, 2967, 1558, 1418; ^1H NMR δ_H (400 MHz, $(CD_3)_2SO$) 8.07 (s, 1H), 6.48 (t, *J* = 2.4 Hz, 1H), 6.36 (m, 1H), 6.04 (t, *J* = 2.4 Hz, 1H), 2.47 (q, *J* = 7.5 Hz, 2H), 1.17 (t, *J* = 7.5 Hz, 3H); ^{13}C NMR δ_C (101 MHz, $(CD_3)_2SO$) 168.3, 165.6, 147.9, 138.7, 125.6, 120.5, 120.3, 117.1, 108.7, 92.9, 78.9, 19.7, 15.3; *m/z* (ESI+) calc'd for $C_{13}H_{12}I_2NO_3$ $[M+H]^+$ 483.8901, found: 483.8913.

HALOGENATED PYRROLE SERIES

3-bromo-1-(triisopropylsilyl)pyrrole (26)

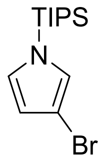

1-(triisopropylsilyl)pyrrole (3.84 g, 17.2 mmol) was dissolved in THF (35 mL), then cooled to -78 °C. *N*-bromosuccinimide (3.07 g, 17.2 mmol) was added and the reaction stirred at -78 °C for 30 minutes, then warmed to room temperature and stirred a further 2.5 hours. The reaction was concentrated *in vacuo*, then petroleum ether (100 mL) added to precipitate the succinimide by-product and the suspension filtered through celite. The filter pad was washed with petroleum ether (4 x 60 mL) and the combined filtrates concentrated *in vacuo*, then purified by flash column chromatography (hexane) yielding 3-bromo-1-(triisopropylsilyl)pyrrole **26** as a colorless oil (4.64 g, 15.3 mmol, 89%); TLC R*f* = 0.40 (hexane); ^1H NMR δ_H (400 MHz, $CDCl_3$) 6.72 (dd, J_1 = 2.6 Hz, J_2 = 1.5 Hz, 1H), 6.67 (t, *J* = 2.6 Hz, 1H), 6.29 (dd, J_1 = 2.6 Hz, J_2 = 1.5 Hz, 1H), 1.42 (m, 3H), 1.09 (d, *J* = 7.5 Hz, 18H); ^{13}C NMR δ_C (101 MHz, $CDCl_3$) 124.8, 123.5, 113.2, 98.1, 17.9, 11.7 ; *m/z* (ESI+) calc'd for $C_{13}H_{25}BrNSi$ $[M+H]^+$ 302.0934, found: 302.0936.

3-chloro-1-(triisopropylsilyl)pyrrole (28)

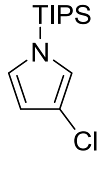

n-butyllithium (2.37 M, hexane) (1.27 mL, 2.54 mmol) was added to a -78 °C solution of 3-bromo-1-(triisopropylsilyl)pyrrole **26** (828 mg, 2.74 mmol) in THF (27 mL) and stirred for 20 minutes. A solution

of *N*-chlorosuccinimide (365 mg, 2.73 mmol) in THF (7 mL) was added, then the reaction stirred at -78 °C for 20 minutes before warming to room temperature and stirring a further 2 hours. The reaction was quenched with a few drops of sat. aq. NH_4Cl, then washed with $NaHCO_3$ (40 mL) and extracted with Et_2O (3 x 40 mL). The combined extracts were dried ($MgSO_4$), concentrated *in vacuo*, then purified by flash column chromatography (hexane) yielding 3-chloro-1-(triisopropylsilyl)pyrrole **28** as a colorless oil (353 mg, 1.37 mmol, 50%); TLC R*f* = 0.60 (hexane); FT-IR (ATR) ν_{max}/cm^{-1} 2947, 2868, 1463, 1223, 1080; ^1H NMR δ_H (400 MHz, $CDCl_3$) 6.68-6.67 (m, 1H), 6.66-6.64 (m, 1H), 6.23 (dd, J_1 = 2.9 Hz, J_2 = 1.4 Hz, 1H), 1.46-1.36 (m, 3H), 1.09 (d, *J* = 7.5 Hz, 18H); ^{13}C NMR δ_C (101 MHz, $CDCl_3$) 124.2, 120.9, 113.9, 110.9, 17.9, 11.6; *m/z* (EI+) calc'd for $C_{13}H_{24}ClNSi$ [$M^{+\bullet}$] 257.1361, found: 257.1361.

Methyl 4-(3-chloro-1*H*-pyrrol-1-yl)-3,5-diiodo-2-methoxy-benzoate (13)

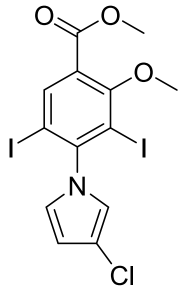

Methyl 4-(3-chloro-1*H*-pyrrol-1-yl)-3,5-diiodo-2-methoxy-benzoate **13** was prepared according to general procedure 6; white solid (57.5 mg, 0.11 mmol, 75%); TLC R*f* = 0.25 (40% CH_2Cl_2 in hexane); m.p. 108 °C; FT-IR (ATR) ν_{max}/cm^{-1} 2989, 1727, 1299, 1230; ^1H NMR δ_H (400 MHz, $CDCl_3$) 8.32 (s, 1H), 6.53 (dd, J_1 = 3.5 Hz, J_2 = 1.8 Hz, 1H), 6.35 (t, *J* = 3.5 Hz, 1H), 6.27 (dd, J_1 = 3.5 Hz, J_2 = 1.8 Hz, 1H), 3.97 (s, 3H), 3.94 (s, 3H); ^{13}C NMR δ_C (101 MHz, $CDCl_3$) 163.8, 160.8, 147.9, 141.3, 127.2, 119.6, 115.3, 110.4, 108.2, 101.0, 92.2, 62.8, 53.1; *m/z* (ESI+) calc'd for $C_{13}H_{10}ClI_2NNaO_3$ $[M+Na]^+$ 539.8331, found: 539.8335.

4-(3-chloro-1*H*-pyrrol-1-yl)-2-hydroxy-3,5-diiodobenzoic acid (20)

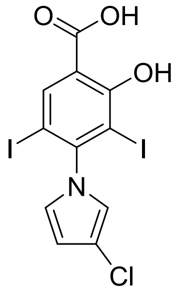

4-(3-chloro-1*H*-pyrrol-1-yl)-2-hydroxy-3,5-diiodobenzoic acid **20** was synthesized according to general procedure 4; white solid (333 mg, 0.68 mmol, 62%); TLC R*f* = 0.20 (10% MeOH in CH_2Cl_2); m.p. 220-221 °C (decomposition); FT-IR (ATR) ν_{max}/cm^{-1} 2988, 2900, 1559, 1414, 1359, 1242; ^{1}H NMR δ_H (400 MHz, $(CD_3)_2SO$) 8.11 (s, 1H), 6.67 (dd, J_1 = 3.2 Hz, J_2 = 2.0 Hz, 1H), 6.21 (m, 2H); ^{13}C NMR δ_C (101 MHz, $(CD_3)_2SO$) 168.3, 165.6, 144.8, 138.5, 120.8, 120.6, 113.8, 109.2, 107.2, 93.9, 79.6; *m/z* (ESI-) calc'd for $C_{11}H_5I_2NO_3$ $[M-H]^-$ 487.8053, found: 487.8047.

3-fluoro-1-(triisopropylsilyl)pyrrole (27)

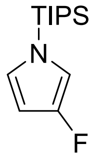

A solution of *n*-butyllithium (2.37 M, hexanes) (1.91 mL, 4.53 mmol) was added to a -78 °C solution of 3-bromo-1-(triisopropylsilyl)pyrrole **26** (1.37 g, 4.54 mmol) in THF (37 mL) and stirred for 5 minutes before addition of a solution of *N*-fluorobenzenesulfonimide (1.44 g, 4.57 mmol) in THF (8 mL). The reaction was warmed to room temperature over 15 minutes, then stirred for a further 30 minutes. The reaction was quenched with a few drops of sat. aq. NH_4Cl, then washed with sat. aq. $NaHCO_3$ (60 mL) and extracted with Et_2O (3 x 60 mL). The combined extracts were washed with sat. aq. NaCl (60 mL), dried ($MgSO_4$) and concentrated *in vacuo*, then purified by flash column chromatography (petroleum ether) yielding 3-fluoro-1-(triisopropylsilyl)pyrrole **27** as a yellow oil (523 mg, 2.17 mmol, 48%); TLC R*f* = 0.50 (petroleum ether); FT-IR (ATR) ν_{max}/cm^{-1} 2947, 2868, 1293; ^{1}H NMR δ_H (400 MHz, $CDCl_3$) 6.48 (m, 2H), 6.06 (m, 1H), 1.40 (m, 3H), 1.09 (d, *J* = 7.5 Hz, 18H); ^{13}C NMR δ_C (101 MHz, $CDCl_3$) 154.1 (d, *J* = 241.4 Hz), 121.6 (d, *J* = 8.1 Hz), 106.2 (d, *J* = 26.4 Hz), 99.7 (d, *J* = 18.3 Hz), 17.9, 11.7; ^{19}F NMR δ_F (376 MHz, $CDCl_3$) -165.68 (m, 1F); *m/z* (ESI+) calc'd for $C_{13}H_{25}FNSi$ $[M+H]^+$ 242.1735, found: 242.1730.

Methyl 3,5-diiodo-4-(3-fluoro-1*H*-pyrrol-1-yl)-2-methoxybenoate (14)

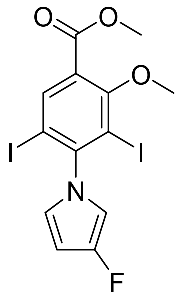

Methyl 3,5-diiodo-4-(3-fluoro-1*H*-pyrrol-1-yl)-2-methoxybenoate **14** was prepared according to general procedure 6; beige solid (731 mg, 1.46 mmol, 73%); TLC R*f* = 0.20 (25% CH_2Cl_2 in hexane); m.p. 96 °C; FT-IR (ATR) ν_{max}/cm^{-1} 2990, 1730, 1566, 1427, 1226; ^{1}H NMR δ_H (400 MHz, $CDCl_3$) 8.30 (s, 1H), 6.39 (ddd, J_1 = 4.1 Hz, J_2 = 2.6 Hz, J_3 = 1.8 Hz, 1H), 6.31 (ddd, J_1 = 4.1 Hz, J_2 = 3.2 Hz, J_3 = 2.6 Hz, 1H), 6.16 (dd, J_1 = 3.2 Hz, J_2 = 1.8 Hz, 1H), 3.96 (s, 3H), 3.92 (s, 3H); ^{13}C NMR δ_C (101 MHz, $CDCl_3$) 163.7, 160.8, 153.2 (d, *J* = 241.4 Hz), 150.0, 141.5, 127.0, 118.1 (d, *J* = 5.9 Hz), 104.3 (d, *J* = 29.3 Hz), 100.2, 99.1 (d, *J* = 16.9 Hz), 91.2, 62.8, 53.1; ^{19}F NMR δ_F (376 MHz, $CDCl_3$) -164.67 (t, *J* = 3.5 Hz, 1F); *m/z* (ESI+) calc'd for $C_{13}H_{11}FI_2NO_3$ [M+H]$^+$ 501.8807, found: 501.8819.

4-(3-fluoro-1*H*-pyrrol-1-yl)-2-hydroxy-3,5-diiodobenzoic acid (21)

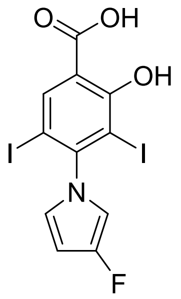

4-(3-fluoro-1*H*-pyrrol-1-yl)-2-hydroxy-3,5-diiodobenzoic acid **21** was prepared according to general procedure 4; purple solid (33.4 mg, 71 µmol, 36%); TLC R*f* = 0.15 (10% MeOH in CH_2Cl_2); m.p. 270-271 °C (decomposition); FT-IR (ATR) ν_{max}/cm^{-1} 2988, 1732, 1566, 1418, 1229; ^{1}H NMR δ_H (400 MHz, $(CD_3)_2SO$) 8.06 (s, 1H) 6.64-6.62 (m, 1H), 6.47-6.44 (m, 1H), 6.07 (dt, J_1 = 3.2 Hz, J_2 = 2.0 Hz, 1H); ^{13}C NMR δ_C (101 MHz, $(CD_3)_2SO$) 168.1, 165.9, 151.7 (d, *J* = 236.0 Hz), 147.1, 138.6, 120.6, 118.6 (d, *J* = 5.7 Hz), 104.5 (d, *J* = 28.4 Hz), 97.3 (d, *J* = 16.7 Hz), 93.2, 78.4; ^{19}F NMR δ_F (376 MHz, $(CD_3)_2SO$) -168.47 (m, 1F); *m/z* (ESI+) calc'd for $C_{11}H_7FI_2NO_3$ [M+H]$^+$ 473.8494, found: 473.8498.

3,4-diiodo-1-(triisopropylsilyl)pyrrole (29)

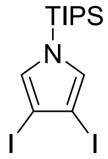

A solution of 1-(triisopropylsilyl)pyrrole **25** (5.00 g, 22.4 mmol) in Et_2O (5 mL) was added dropwise to a solution of iodine (8.51 g, 33.5 mmol) and H_5IO_6 (2.52 g, 11.1 mmol) in Et_2O (50 mL) and the reaction stirred at room temperature. After 1 hour, the reaction was quenched with sat. aq. $Na_2S_2O_3$ (2 mL) and washed with sat. aq. $Na_2S_2O_3$ (4 x 50 mL) followed by water (50 mL) and sat. aq. NaCl (50 mL). The organic phase was dried ($MgSO_4$) and concentrated *in vacuo* yielding 3,4-diiodo-1-(triisopropylsilyl)pyrrole **29** as beige crystals (9.12 g, 19.2 mmol, 86%); TLC R*f* = 0.75 (petroleum ether); m.p. 78-79 °C; FT-IR (ATR) ν_{max}/cm^{-1} 2945, 2864, 1458; ^1H NMR δ_H (400 MHz, $CDCl_3$) 6.79 (s, 2H), 1.47-1.35 (m, 3H), 1.08 (d, *J* = 7.5 Hz, 18H); ^{13}C NMR δ_C (101 MHz, $CDCl_3$) 129.8, 75.3, 17.8, 11.6; *m/z* (ESI+) calc'd for $C_{13}H_{23}I_2NSi$ [$M^{+\bullet}$] 474.9684, found: 474.9689.

3,4-difluoro-1-(triisopropylsilyl)-pyrrole (30)

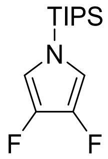

At -78 °C, a solution of *n*BuLi (2.41 M, hexanes) (4.30 mL, 10.4 mmol) was added to a solution of 3,4-diiodo-1-(triisopropylsilyl)pyrrole **29** (4.92 g, 10.4 mmol) in THF (100 mL) and stirred for 5 minutes before addition of a solution of NFSI (3.28 g, 10.4 mmo) in THF (10 mL). The reaction was warmed to room temperature and stirred for 30 minutes, then cooled again to -78 °C. A solution of *n*BuLi (2.41 M, hexanes) (4.30 mL, 10.4 mmol) was added, and the reaction was stirred for 5 minutes before addition of a solution of NFSI (3.28 g, 10.4 mmol) in THF (10 mL). The reaction was warmed to room temperature and stirred for 30 minutes, then quenched with sat. aq. NH_4Cl (0.5 mL). The reaction was washed with sat. aq. $NaHCO_3$ (120 mL) and extracted with Et_2O (3 x 120 mL). The organic phases were washed with brine (120 mL), dried ($MgSO_4$), then concentrated *in vacuo*. The crude oil was purified by flash column chromatography (hexane) yielding 3,4-difluoro-1-(triisopropylsilyl)-pyrrole **30** as a yellow solid (857 mg, 3.30 mmol, 32%); TLC R*f* = 0.65 (hexane); m.p. 41-42 °C; FT-IR (ATR) ν_{max}/cm^{-1} 2867, 1569, 1338; ^1H NMR δ_H (400 MHz, $CDCl_3$) 6.32 (s, 2H), 1.42-1.31 (m, 3H), 1.08 (d, *J* = 7.5 Hz, 18H); ^{13}C NMR δ_C (101 MHz, $CDCl_3$) 140.7 (dd, J_1 = 241.0 Hz, J_2 = 12.8 Hz), 105.4 (dd, J_1 = 18.7 Hz, J_2 = 5.5 Hz), 17.8, 11.5; ^{19}F NMR δ_F (376 MHz, $CDCl_3$) -178.0 (s, 2F); *m/z* (ESI+) calc'd for $C_{13}H_{24}F_2NSi$ $[M+H]^+$ 260.1641, found: 260.1638.

Methyl 4-(3,4-difluoro-1*H*-pyrrol-1-yl)-3,5-diiodo-2-methoxybenzoate (15)

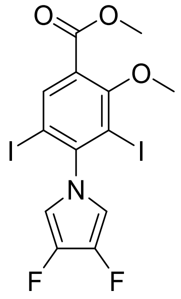

Methyl 4-(3,4-difluoro-1*H*-pyrrol-1-yl)-3,5-diiodo-2-methoxybenzoate **15** was synthesized according to general procedure 6; (216 mg, 0.42 mmol, 80%); TLC R*f* = 0.15 (25% CH_2Cl_2 in hexane); m.p. 128-129 °C; FT-IR (ATR) ν_{max}/cm^{-1} 3120, 1704, 1568, 1431, 1350, 1240; ^1H NMR δ_H (400 MHz, $CDCl_3$) 8.29 (s, 1H), 6.25 (d, *J* = 1.1 Hz, 2H), 3.96 (s, 3H), 3.92 (s, 3H); ^{13}C NMR δ_C (101 MHz, $CDCl_3$) 163.6, 160.8, 149.3, 141.6, 140.0 (dd, J_1 = 242.1 Hz, J_2 = 11.7 Hz), 127.3, 103.5, (m), 100.3, 91.2, 62.8, 53.1; ^{19}F NMR δ_F (376 MHz, $CDCl_3$) -178.0 (s, 2F); *m/z* (ESI+) calc'd for $C_{13}H_{10}F_2I_2NO_3$ $[M+H]^+$ 519.8713, found: 519.8710.

4-(3,4-difluoro-1*H*-pyrrol-1-yl)-2-hydroxy-3,5-diiodobenzoic acid (22)

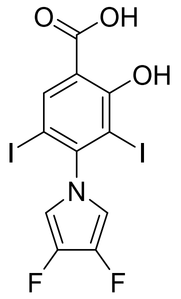

4-(3,4-difluoro-1*H*-pyrrol-1-yl)-2-hydroxy-3,5-diiodobenzoic acid **22** was prepared according to general procedure 4; brown solid (93.1 mg, 0.19 mmol, 60%); TLC R*f* = 0.30 (15% MeOH in CH_2Cl_2); m.p. 217-218 °C (decomposition); FT-IR (ATR) ν_{max}/cm^{-1} 3125, 2954, 1571, 1413, 1385, 1239; ^1H NMR δ_H (400 MHz, $(CD_3)_2SO$) 8.04 (s, 1H), 6.71 (d, *J* = 1.5 Hz, 2H); ^{13}C NMR δ_C (101 MHz, $(CD_3)_2SO$) 168.3, 165.4, 146.9, 138.7, 138.1 (dd, J_1 = 237.0 Hz, J_2 = 11.7 Hz), 120.2, 104.2 (m), 93.4, 79.3; ^{19}F NMR δ_F (376 MHz, $(CD_3)_2SO$) -180.7 (s, 2F); *m/z* (ESI+) calc'd for $C_{11}H_6F_2I_2NO_3$ $[M+H]^+$ 491.8400, found: 491.8409.

3-iodo-1-(triisopropylsilyl)pyrrole (31)

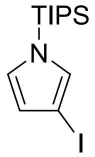

A solution of *N*-iodosuccinimide (4.92 g, 21.9 mmol) in acetone (70 mL) was added at -78 °C to a solution of 1-(triisopropylsilyl)pyrrole **25** (4.06 g, 18.2 mmol) in acetone (110 mL). The reaction was stirred at -78 °C for 1 hour, then at room temperature for 4 hours. The reaction was concentrated *in vacuo*, then petroleum ether (200 mL) was added to precipitate the succinimide by-product and the suspension filtered through a plug of silica. The filter pad was washed thoroughly with petroleum ether and the combined filtrate concentrated *in vacuo* to a colorless oil. 3-iodo-1-(triisopropylsilyl)pyrrole **31** was used in the next step without further purification (6.20 g, crude); TLC R*f* = 0.50 (hexane); FT-IR (ATR) ν_{max}/cm^{-1} 2945, 2867, 1464, 1206; ^1H NMR δ_H (400 MHz, $CDCl_3$) 6.79 (dd, J_1 = 2.5 Hz, J_2 = 1.3 Hz, 1H), 6.66 (t, J = 2.5 Hz, 1H), 6.36 (dd, J_1 = 2.5 Hz, J_2 = 1.3 Hz, 1H), 1.42 (m, 3H), 1.09 (d, J = 7.6 Hz, 18H); ^{13}C NMR δ_C (101 MHz, $CDCl_3$) 128.9, 125.9, 117.7, 62.3, 17.9, 11.7; *m/z* (EI+) calc'd for $C_{13}H_{24}INSi$ [$M^{+\bullet}$] 349.0717, found: 349.0721.

3-iodo-1*H*-pyrrole (32)

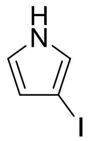

3-iodo-1-(triisopropylsilyl)pyrrole **31** (6.20 g, crude) was dissolved in THF (33 mL) under Ar, then TBAF (1M, THF) (27 mL, 27 mmol) added and the reaction stirred at room temperature for 1 hour. sat. aq. $NaHCO_3$ (100 mL) was added and the mixture extracted with Et_2O (3 x 100 mL). The organic layer washed with sat. aq. NaCl (100 mL), dried ($MgSO_4$) and concentrated *in vacuo* to an orange oil. 3-iodo-1*H*-pyrrole **32** was used in the next step without further purification (8.01 g, crude); TLC R*f* = 0.10 (petroleum ether); FT-IR (ATR) ν_{max}/cm^{-1} 3274 br., 2941, 2864, 1463; ^1H NMR δ_H (400 MHz, $CDCl_3$) 8.42 (br. s, 1H), 6.87-6.84 (m, 1H), 6.71 (q, J = 2.6 Hz, 1H), 6.32 (td, J_1 = 2.6 Hz, J_2 = 1.5 Hz, 1H); ^{13}C NMR δ_C (101 MHz, $CDCl_3$) 123.0, 119.8, 115.9, 60.0; *m/z* (EI+) calc'd for C_4H_4IN [$M^{+\bullet}$] 192.9384, found: 192.9383.

3-iodo-1-(4-toluenesulfonyl)pyrrole (33)

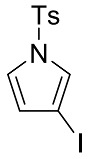

A 0 °C solution of 3-iodo-1*H*-pyrrole **32** (8.01 g, crude) in THF (20 mL) was added to a 0 °C solution of NaH (60%, mineral oil) (1.10 g, 27.5 mmol) in THF (20 mL) and stirred for 15 minutes. A 0 °C solution of 4-toluenesulfonyl chloride (5.21 g, 27.3 mmol) in THF (20 mL) was added and the reaction stirred for 10 minutes before warming to room temperature and stirring for 1 hour. The reaction was quenched by slow addition of H_2O (1 mL), then sat. aq. $NaHCO_3$ (100 mL) was added and the mixture extracted with CH_2Cl_2 (3 x 100 mL). The organic layer was washed with sat. aq. NaCl (100 mL), dried ($MgSO_4$) and concentrated *in vacuo*. The crude oil was purified by flash column chromatography (5% EtOAc in petroleum ether) yielding 3-iodo-1-tosylpyrrole **33** as a white solid (5.31 g, 15.3 mmol, 84% over 3 steps). Triisopropylsilanol by-product could also be removed by trituration in hexane; TLC R*f* = 0.25 (5% EtOAc in petroleum ether); m.p. 97-98 °C; FT-IR (ATR) ν_{max}/cm^{-1} 2988, 1370, 1168; ^1H NMR δ_H (400 MHz, $CDCl_3$) 7.75 (d, *J* = 8.1 Hz, 2H), 7.31 (d, *J* = 8.1 Hz, 2H), 7.22 (dd, J_1 = 2.3 Hz, J_2 = 1.5 Hz, 1H), 7.03 (dd, J_1 = 3.2 Hz, J_2 = 2.3 Hz, 1H), 6.34 (dd, J_1 = 3.2 Hz, J_2 = 1.5 Hz, 1H), 2.42 (s, 3H); ^{13}C NMR δ_C (101 MHz, $CDCl_3$) 145.7, 135.7, 130.3 , 127.2 , 124.8, 122.2, 120.5, 66.7, 21.8; *m/z* (ESI+) calc'd for $C_{11}H_{10}INNaO_2S$ $[M+Na]^+$ 369.9369, found: 369.9374.

1-tosyl-3-(trifluoromethyl)-pyrrole (34)

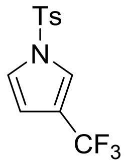

Methyl fluorosulfonyldifluoroacetate (4.50 mL, 35.3 mmol) was added to a solution of 3-iodo-1-tosylpyrrole **33** (2.02 g, 5.82 mmol), CuI (1.33 g, 6.98 mmol) and HMPA (5.80 mL, 29.1 mmol) in DMF (58 mL), then heated to 70 °C for 20 hours. The reaction was then cooled to room temperature, poured carefully onto sat. aq. $NaHCO_3$ (400 mL), then extracted with Et_2O (3 x 400 mL). The organic layer was washed with sat. aq. NaCl (400 mL), dried ($MgSO_4$) and concentrated *in vacuo*. The crude oil was purified by flash column chromatography (20% CH_2Cl_2 in petroleum ether) yielding 1-tosyl-3-(trifluoromethyl)-pyrrole **34** as a white solid (1.04 g, 3.59 mmol, 62%); TLC R*f* = 0.20 (20% CH_2Cl_2 in petroleum ether); m.p. 62-63 °C; FT-IR (ATR) ν_{max}/cm^{-1} 2988, 1375, 1172; ^1H NMR δ_H (400 MHz, $CDCl_3$) 7.79 (d, *J* = 8.4 Hz, 2H), 7.47 (m, 1H), 7.34 (d, *J* = 8.4 Hz, 2H), 7.17 (m, 1H), 6.43 (m, 1H), 2.43 (s, 3H); ^{13}C NMR δ_C (101 MHz, $CDCl_3$) 146.2, 135.2, 130.5 , 127.4 , 122.6 (q, *J* = 267.0 Hz), 121.8, 120.1 (q, *J* =

5.3 Hz), 119.6 (q, J = 37.9 Hz), 110.3 (q, J = 2.4 Hz), 21.8; ^{19}F NMR δ_F (376 MHz, $CDCl_3$) -58.71 (s, 3F); *m/z* (EI+) calc'd for $C_{12}H_{10}F_3NO_2S$ [$M^{+\bullet}$] 289.0378, found: 289.0378.

3-(trifluoromethyl)-1*H*-pyrrole (35)

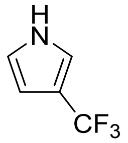

1-tosyl-3-(trifluoromethyl)-pyrrole **34** (563 mg, 1.95 mmol) and magnesium (471 mg, 19.4 mmol) were dissolved in dry MeOH (20 mL) under Ar and sonicated at room temperature for 30 minutes. The reaction was added to sat. aq. NH_4Cl (200 mL) and extracted with Et_2O (3 x 200 mL). The organic layer was washed with sat. aq. NaCl (200 mL), dried ($MgSO_4$), and concentrated *in vacuo* yielding 3-(trifluoromethyl)-1*H*-pyrrole **35** as a yellow oil (192 mg, 1.42 mmol, 73%); TLC R*f* = 0.20 (25% Et_2O in pentane); FT-IR (ATR) ν_{max}/cm^{-1} 3486, 1362; ^{1}H NMR δ_H (400 MHz, $CDCl_3$) 8.44 (m, 1H), 7.11 (br s, 1H), 6.81 (m, 1H), 6.43 (m, 1H); ^{13}C NMR δ_C (101 MHz, $CDCl_3$) 124.1 (q, J = 265.7 Hz), 119.1, 117.7 (q, J = 4.8 Hz), 115.4 (q, J = 36.8 Hz), 106.5 (q, J = 2.9 Hz); ^{19}F NMR δ_F (376 MHz, $CDCl_3$) -56.79 (s, 3F); *m/z* (EI+) calc'd for $C_5H_4F_3N$ [$M^{+\bullet}$] 135.0290, found: 135.0290.

Methyl 3,5-diiodo-2-methoxy-4-(3-trifluoromethyl-1*H*-pyrrol-1-yl)benzoate (16)

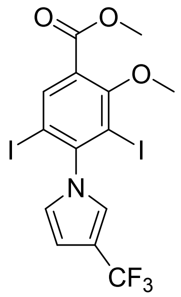

Methyl 3,5-diiodo-2-methoxy-4-(3-trifluoromethyl-1*H*-pyrrol-1-yl)benzoate **16** was prepared according to general procedure 3; white solid (73.1 mg, 0.133 mmol, 64%); TLC R*f* = 0.10 (20% CH_2Cl_2 in hexane); m.p. 96-97 °C; FT-IR (ATR) ν_{max}/cm^{-1} 2952, 1732, 1278, 1226; ^{1}H NMR δ_H (400 MHz, $CDCl_3$) 8.32 (s, 1H), 6.94-6.91 (m, 1H), 6.61-6.59 (m, 1H), 6.57-6.55 (m, 1H), 3.97 (s, 3H), 3.93 (s, 3H); ^{13}C NMR δ_C (101 MHz, $CDCl_3$) 163.6, 160.9, 149.2, 141.7, 127.4, 123.7 (q, J = 266.3 Hz), 122.0, 120.4 (q, J = 4.7 Hz), 116.9 (q, J = 37.4 Hz), 108.0 (q, J = 2.9 Hz), 99.7, 90.6, 62.8, 53.2; ^{19}F NMR δ_F (376 MHz, $CDCl_3$) -57.6 (s, 3F); *m/z* (ESI+) calc'd for $C_{14}H_{11}F_3I_2NO_3$ [M+H]$^+$ 551.8775, found: 551.8770.

2-hydroxy-3,5-diiodo-4-(3-(trifluoromethyl)-1*H*-pyrrol-1-yl)benzoic acid (23)

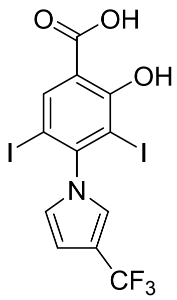

In a sealed microwave tube, iodotrimethylsilane (60.0 µL, 0.42 mmol) was added to a solution of methyl 3,5-diiodo-2-methoxy-4-(3-trifluoromethyl-1*H*-pyrrol-1-yl)benzoate **16** (40.0 mg, 72.6 µmol) in CH_2Cl_2 (1.45 mL) and heated to 50 °C for 24 hours. The reaction was quenched with MeOH, concentrated *in vacuo*, and purified by flash column chromatography yielding 2-hydroxy-3,5-diiodo-4-(3-(trifluoromethyl)-1*H*-pyrrol-1-yl)benzoic acid **23** as a yellow solid (11.9 mg, 22.8 µmol, 31%); TLC R*f* = 0.20 (15% MeOH in CH_2Cl_2); m.p. 147 °C (decomposition); FT-IR (ATR) ν_{max}/cm^{-1} 3422 br., 1558, 1420; ^{1}H NMR δ_H (400 MHz, $(CD_3)_2CO$) 14.70 (br. s, 1H), 8.48 (br. s, 1H), 7.12 (br. s, 1H), 6.73 (br. s, 1H), 6.49 (br. s, 1H); ^{13}C NMR δ_C (101 MHz, $(CD_3)_2CO$) 172.7, 163.4, 148.5, 142.2, 125.1 (q, *J* = 265.0 Hz), 123.8, 123.6 (m), 121.8 (q, *J* = 4.8 Hz), 115.8 (q, *J* = 37.0 Hz), 107.4 (m), 90.1, 82.3; ^{19}F NMR δ_F (376 MHz, $(CD_3)_2CO$) -57.5 (s, 3F); *m/z* (ESI+) calc'd for $C_{12}H_7F_3I_2NO_3$ $[M+H]^+$ 523.8462, found: 523.8471.

4-(HETERO)ALKYLSALICYLIC ACID SERIES

7-hydroxy-2,2-dimethyl-4*H*-benzo[*d*][1,3]dioxin-4-one (56)

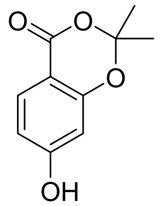

Trifluoroacetic anhydride (22.5 mL, 160 mmol) and acetone (13.7 mL, 187 mmol) were added at 0 °C to a suspension of 2,4-dihydroxybenzoic acid (5.01 g, 32.3 mmol) in trifluoroacetic acid (45.5 mL) and stirred for 24 hours. The reaction was concentrated *in vacuo*, then washed with sat. aq. $NaHCO_3$ (100 mL) and extracted with EtOAc (4 x 100 mL). The organic layers were washed sequentially with H_2O (150 mL) and sat. aq. NaCl (150 mL), dried ($MgSO_4$) and concentrated *in vacuo*. The crude solid was triturated in dichloromethane, recovered by filtration, then dried under high vacuum yielding 7-

hydroxy-2,2-dimethyl-4*H*-benzo[*d*][1,3]dioxin-4-one **56** as a white solid which was used in the next step without further purification (2.79 g, 14.4 mmol, 45%); TLC R*f* = 0.60 (20% EtOAc in CH_2Cl_2); m.p. 187-188 °C; FT-IR (ATR) ν_{max}/cm^{-1} 3142 br, 1697, 1616, 1258; ^1H NMR δ_H (400 MHz, $(CD_3)_2SO$) 10.82 (s, 1H), 7.69 (d, *J* = 8.6 Hz, 1H), 6.60 (dd, J_1 = 8.6 Hz, J_2 = 2.3 Hz, 1H), 6.38 (d, *J* = 2.3 Hz, 1H), 1.65 (s, 6H); ^{13}C NMR δ_C (101 MHz, $(CD_3)_2SO$) 165.2, 160.1, 157.4, 131.1, 111.5, 105.9, 104.4, 102.6, 25.3 ; *m/z* (ESI+) calc'd for $C_{10}H_{10}NaO_4$ $[M+Na]^+$ 217.0471, found: 217.0475.

7-hydroxy-6,8-diiodo-2,2-dimethyl-4*H*-benzo[*d*][1,3]dioxin-4-one (57)

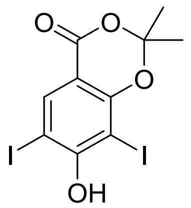

7-hydroxy-2,2-dimethyl-4*H*-benzo[*d*][1,3]dioxin-4-one **56** (2.61 g, 13.5 mmol) was dissolved in THF (25 mL) at 0 °C and *N*-iodosuccinimide (6.36 g, 28.3 mmol) added in portions. After 1 hour, 2M aq. NaS_2O_3 (1 mL) was added, then the solvent removed *in vacuo*. The residue was dissolved in EtOAc (250 mL) and washed with a mixture of H_2O (200 mL) and sat. aq. NaCl (20 mL), then with sat. aq. NaCl (200 mL). The organic layer was dried ($MgSO_4$), concentrated *in vacuo*, then purified by flash column chromatography (20% EtOAc in CH_2Cl_2) yielding 7-hydroxy-6,8-diiodo-2,2-dimethyl-4*H*-benzo[*d*][1,3]dioxin-4-one **57** as a brown solid (4.40 g, 9.87 mmol, 73%); TLC R*f* = 0.50 (20% EtOAc in CH_2Cl_2); m.p. 174-5 °C; FT-IR (ATR) ν_{max}/cm^{-1} 3305, 1708, 1582, 1554, 1423, 1272, 1246, 1228; ^1H NMR δ_H (400 MHz, $(CD_3)_2SO$) 8.10 (s, 1H), 1.69 (s, 6H); ^{13}C NMR δ_C (101 MHz, $(CD_3)_2SO$) 162.8, 158.5, 156.9, 138.7, 107.5, 107.1, 78.8, 76.7, 25.4 ; *m/z* (ESI+) calc'd for $C_{10}H_9I_2O_4$ $[M+H]^+$ 446.8585, found: 446.8578.

6,8-diiodo-7-methoxy-2,2-dimethyl-4*H*-benzo[*d*][1,3]dioxin-4-one (58)

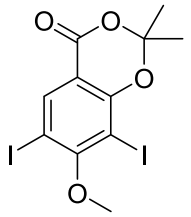

6,8-diiodo-7-methoxy-2,2-dimethyl-4*H*-benzo[*d*][1,3]dioxin-4-one **58** was synthesized according to general procedure 7; white solid (202 mg, 0.44 mmol, 44%); TLC R*f* = 0.35 (50% CH_2Cl_2 in petroleum ether); m.p. 98-99 °C; FT-IR (ATR) ν_{max}/cm^{-1} 2979, 2943, 1724, 1583, 1407, 1379, 1276, 1216; ^1H NMR

δ_H (400 MHz, $CDCl_3$) 8.38 (s, 1H), 3.92 (s, 3H), 1.78 (s, 6H); ^{13}C NMR δ_C (101 MHz, $CDCl_3$) 165.7, 158.9, 157.6, 140.3, 112.1, 107.9, 82.4, 82.1, 61.0, 26.0 ; *m/z* (ESI+) calc'd for $C_{11}H_{11}I_2O_4$ $[M+H]^+$ 460.8741, found: 460.8743.

6,8-diiodo-2,2-dimethyl-7-ethoxy-4*H*-benzo[*d*][1,3]dioxin-4-one (59)

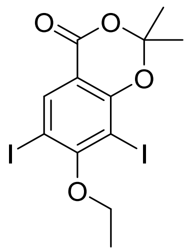

6,8-diiodo-2,2-dimethyl-7-ethoxy-4*H*-benzo[*d*][1,3]dioxin-4-one **59** was synthesized according to general procedure 7; white solid (203 mg, 0.43 mmol, 74%); TLC R*f* = 0.40 (50% CH_2Cl_2 in petroleum ether); m.p. 88-89 °C; FT-IR (ATR) ν_{max}/cm^{-1} 3060, 2978, 2943, 1725, 1583, 1407, 1379, 1277, 1212; ^1H NMR δ_H (400 MHz, $CDCl_3$) 8.37 (s, 1H), 4.11 (q, *J* = 7.0 Hz, 2H), 1.77 (s, 6H), 1.57 (t, *J* = 7.1 Hz, 3H); ^{13}C NMR δ_C (101 MHz, $CDCl_3$) 165.0, 159.0, 157.5, 140.2, 111.8, 107.8, 83.0, 82.5, 70.0, 26.0 , 15.6; *m/z* (ESI+) calc'd for $C_{12}H_{13}I_2O_4$ $[M+H]^+$ 474.8898, found: 474.8900.

6,8-diiodo-2,2-dimethyl-7-propoxy-4*H*-benzo[*d*][1,3]dioxin-4-one (60)

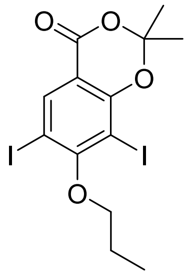

6,8-diiodo-2,2-dimethyl-7-propoxy-4*H*-benzo[*d*][1,3]dioxin-4-one **60** was synthesized according to general procedure 8; white solid (186 mg, 0.38 mmol, 68%); TLC R*f* = 0.75 (50% CH_2Cl_2 in petroleum ether); m.p. 123 °C; FT-IR (ATR) ν_{max}/cm^{-1} 2980, 2942, 2875, 1726, 1581, 1406, 1390, 1277, 1225; ^1H NMR δ_H (400 MHz, $CDCl_3$) 8.38 (s, 1H), 4.01 (t, *J* = 6.6 Hz, 2H), 1.99 (m, 2H), 1.77 (s, 6H), 1.14 (t, *J* = 7.4 Hz, 3H); ^{13}C NMR δ_C (101 MHz, $CDCl_3$) 164.9, 159.0, 157.6, 140.3, 111.8, 107.8, 82.9, 82.4, 75.4, 26.0 , 23.6, 10.7; *m/z* (ESI+) calc'd for $C_{13}H_{15}I_2O_4$ $[M+H]^+$ 488.9054, found: 488.9060.

6,8-diiodo-2,2-dimethyl-7-(3,3,3-trifluoropropoxy)-4*H*-benzo[*d*][1,3]dioxin-4-one (61)

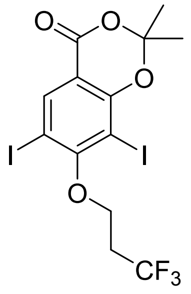

6,8-diiodo-2,2-dimethyl-7-(3,3,3-trifluoropropoxy)-4*H*-benzo[*d*][1,3]dioxin-4-one **61** was synthesized according to general procedure 8; white solid (388 mg, 0.72 mmol, 63%); TLC R*f* = 0.75 (50% CH_2Cl_2 in petroleum ether); m.p. 110-111 °C; FT-IR (ATR) ν_{max}/cm^{-1} 3000, 2943, 1725, 1583, 1409, 1391, 1278, 1225; ^1H NMR δ_H (400 MHz, $CDCl_3$) 8.39 (s, 1H), 4.25 (t, *J* = 6.8 Hz, 2H), 2.83 (qt, J_1 = 10.5 Hz, J_2 = 6.8 Hz, 2H), 1.78 (s, 6H); ^{13}C NMR δ_C (101 MHz, $CDCl_3$) 163.9, 158.8, 157.6, 140.4, 125.8 (q, *J* = 276.6 Hz), 112.4, 108.0, 82.4, 82.3, 66.0 (q, *J* = 3.6 Hz), 34.8 (q, *J* = 29.6 Hz), 26.0; ^{19}F NMR δ_F (376 MHz, $CDCl_3$) -63.9 (t, *J* = 10.4 Hz, 3F); *m/z* (ESI+) calc'd for $C_{13}H_{12}F_3I_2O_4$ $[M+H]^+$ 542.8772, found: 542.8765.

2-hydroxy-3,5-diiodo-4-(2,2,2-trifluoroethoxy)benzoic acid (24)

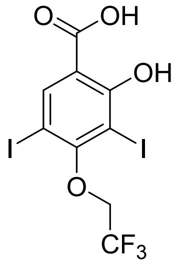

NaH (60%, mineral oil) (331 mg, 8.28 mmol) was added to a solution of 2,2,2-trifluoroethanol (0.50 mL, 6.84 mmol) in DMF (4 mL) at 0 °C and warmed to room temperature over 30 minutes. 4-fluoro-2-hydroxy-3,5-diiodobenzoic acid **9** (280 mg, 0.69 mmol) was added and the reaction heated to 150 °C for 20 hours. The reaction was cooled to room temperature, added to 1M HCl (80 mL), then extracted with 4:1 $CHCl_3$:*i*PrOH (2 x 80 mL). The organic phases were dried ($MgSO_4$), concentrated *in vacuo*, then purified by flash column chromatography (10% MeOH in CH_2Cl_2) yielding 2-hydroxy-3,5-diiodo-4-(2,2,2-trifluoroethoxy)benzoic acid **24** as a pink solid (72.8 mg, 0.15 mmol, 22%); TLC R*f* = 0.25 (10% MeOH in CH_2Cl_2); m.p. 218-219 °C (decomposition); FT-IR (ATR) ν_{max}/cm^{-1} 2997, 1774, 1729, 1583, 1413, 1279; ^1H NMR δ_H (400 MHz, $(CD_3)_2SO$) 8.01 (s, 1H), 4.47 (q, *J* = 8.8 Hz); ^{13}C NMR δ_C (101 MHz, $(CD_3)_2SO$) 168.7, 166.5, 157.3, 139.7, 123.2 (q, *J* = 278.4 Hz), 118.4, 84.4, 71.6, 67.6 (q, *J* = 34.0 Hz); ^{19}F

NMR δ_F (376 MHz, $(CD_3)_2SO$) -67.43 (t, *J* = 8.7 Hz, 3F); *m/z* (ESI-) calc'd for $C_9H_4F_3I_2O_4$ $[M-H]^-$ 486.8157, found: 486.8163.

2-hydroxy-3,5-diiodo-4-methoxybenzoic acid (62)

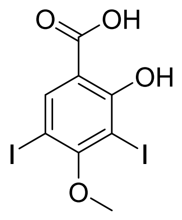

2-hydroxy-3,5-diiodo-4-methoxybenzoic acid **62** was synthesized according to general procedure 9; white solid (120 mg, 0.34 mmol, 85%); TLC R*f* = 0.45 (20% MeOH in CH_2Cl_2); m.p. 197-198 °C (decomposition); FT-IR (ATR) ν_{max}/cm^{-1} 3581, 2938, 1553, 1409, 1344, 1253; ^1H NMR δ_H (400 MHz, $(CD_3)_2SO$) 8.03 (s, 1H), 3.71 (s, 3H); ^{13}C NMR δ_C (101 MHz, CD_3OD) 168.7, 166.2, 161.1, 139.2, 117.4, 84.4, 72.4, 59.8; *m/z* (ESI+) calc'd for $C_8H_6I_2O_4$ $[M+H]^+$ 420.8428, found: 420.8433.

4-ethoxy-2-hydroxy-3,5-diiodobenzoic acid (63)

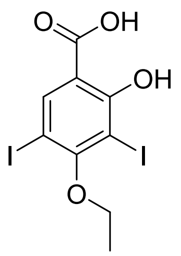

4-ethoxy-2-hydroxy-3,5-diiodobenzoic acid **63** was synthesized according to general procedure 9; beige solid (134 mg, 0.31 mmol, 77%); R*f* = 0.35 (20% MeOH in CH_2Cl_2); m.p. 194-195 °C (decomposition); FT-IR (ATR) ν_{max}/cm^{-1} 2972, 1613, 1555, 1409, 1343, 1252; ^1H NMR δ_H (400 MHz, $(CD_3)_2SO$) 8.02 (s, 1H), 3.92 (q, *J* = 7.0 Hz, 2H), 1.41 (t, *J* = 7.0 Hz, 3H); ^{13}C NMR δ_C (101 MHz, $(CD_3)_2SO$) 168.8, 166.1, 160.1, 139.2, 117.6, 84.8, 73.1, 68.3, 15.4; *m/z* (ESI-) calc'd for $C_9H_7I_2O_4$ $[M-H]^-$ 432.8439, found: 432.8429.

2-hydroxy-3,5-diiodo-4-propoxybenzoic acid (64)

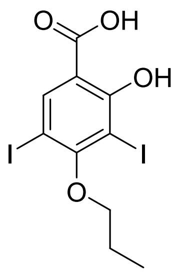

2-hydroxy-3,5-diiodo-4-propoxybenzoic acid **64** was synthesized according to general procedure 9; pink solid (126 mg, 0.28 mmol, 88%); R*f* = 0.40 (20% MeOH in CH_2Cl_2); m.p. 181-182 °C (decomposition); FT-IR (ATR) ν_{max}/cm^{-1} 3485, 2461, 1611, 1553, 1411, 1336, 1251; ^1H NMR δ_H (400 MHz, $(CD_3)_2SO$) 8.01 (s, 1H), 3.83 (t, *J* = 6.5 Hz, 2H), 1.83 (m, 2H), 1.06 (t, *J* = 7.5 Hz, 3H); ^{13}C NMR δ_C (101 MHz, $(CD_3)_2SO$) 168.9, 166.0, 160.1, 139.3, 117.3, 84.8, 73.8, 73.1, 23.0, 10.6; *m/z* (ESI-) calc'd for $C_{10}H_9I_2O_4$ $[M-H]^-$ 446.8596, found: 446.8595.

2-hydroxy-3,5-diiodo-4-(3,3,3-trifluoropropoxy)benzoic acid (65)

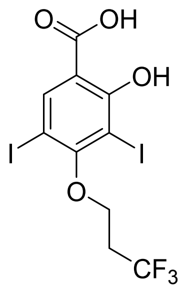

2-hydroxy-3,5-diiodo-4-(3,3,3-trifluoropropoxy)benzoic acid **65** was synthesized according to general procedure 9; pink solid (291 mg, 0.58 mmol, 81%); R*f* = 0.45 (20% MeOH in CH_2Cl_2); m.p. 182-183 °C (decomposition); FT-IR (ATR) ν_{max}/cm^{-1} 3405, 2966, 1612, 1553, 1413, 1337, 1250; ^1H NMR δ_H (400 MHz, $(CD_3)_2SO$) 8.04 (s, 1H), 4.08 (t, *J* = 5.9 Hz, 2H), 2.88 (qt, J_1 = 11.3 Hz, J_2 = 5.9 Hz, 2H); ^{13}C NMR δ_C (101 MHz, $(CD_3)_2SO$) 168.9, 165.9, 159.8, 139.4, 126.6 (d, *J* = 276.8 Hz), 117.5, 84.6, 72.9, 65.2 (q, *J* = 3.3 Hz), 33.8 (q, *J* = 28.3 Hz); ^{19}F NMR δ_F (376 MHz, $(CD_3)_2SO$) -57.97 (t, *J* = 11.3 Hz, 3F); *m/z* (ESI-) calc'd for $C_{10}H_6F_3I_2O_4$ $[M-H]^-$ 500.8313, found: 500.8324.

2-hydroxy-3,5-diiodo-4-methylbenzoic acid (74)

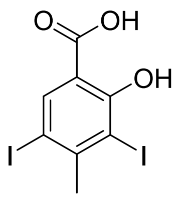

2-hydroxy-3,5-diiodo-4-methylbenzoic acid **74** was synthesized according to general procedure 1; beige solid (175 mg, 0.43 mmol, 88%); TLC R*f* = 0.15 (10% MeOH in CH_2Cl_2); m.p. 193 °C; FT-IR (ATR) ν_{max}/cm^{-1} 2947, 1699, 1227; ^1H NMR δ_H (400 MHz, $(CD_3)_2SO$) 8.17 (s, 1H), 2.73 (s, 3H); ^{13}C NMR δ_C (101 MHz, $(CD_3)_2SO$) 170.3, 160.3, 150.2, 139.2, 112.7, 92.6, 86.6, 35.3; *m/z* (ESI-) calc'd for $C_8H_5I_2O_3$ $[M-H]^-$ 402.8334, found: 402.8338.

4-ethyl-2-hydroxy-3,5-diiodobenzoic acid (75)

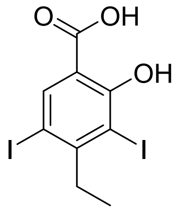

4-ethyl-2-hydroxy-3,5-diiodobenzoic acid **75** was synthesized according to general procedure 1; beige solid (79.9 mg, 0.19 mmol, 98%); TLC R*f* = 0.10 (10% MeOH in CH_2Cl_2); m.p. 188-189 °C (decomposition); FT-IR (ATR) ν_{max}/cm^{-1} 2969, 1667, 1225; ^1H NMR δ_H (400 MHz, $(CD_3)_2SO$) 8.16 (s, 3H), 3.08 (q, *J* = 7.5 Hz, 2H), 1.07 (t, *J* = 7.5 Hz, 3H); ^{13}C NMR δ_C (101 MHz, CD_3OD) 171.8, 162.5, 156.1, 141.8, 113.9, 91.2, 84.9, 41.7, 12.7; *m/z* (ESI+) calc'd for $C_9H_9I_2O_3$ $[M+H]^+$ 418.8636, found: 418.8635.

2-hydroxy-3,5-diiodo-4-propylbenzoic acid (76)

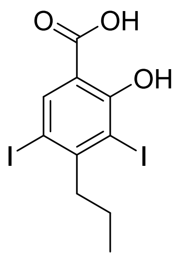

2-hydroxy-3,5-diiodo-4-propylbenzoic acid **76** was synthesized according to general procedure 1; brown solid (71.5 mg, 0.21 mmol, 80%); TLC R*f* = 0.20 (15% MeOH in CH_2Cl_2); m.p. 180-181 °C (decomposition); FT-IR (ATR) ν_{max}/cm^{-1} 2955, 2865, 1670, 1225; ^1H NMR δ_H (400 MHz, $(CD_3)_2SO$) 8.16 (s, 1H), 3.04-2.98 (m, 2H), 1.53-1.43 (m, 2H), 1.03 (t, *J* = 7.3 Hz, 3H); ^{13}C NMR δ_C (101 MHz, $(CD_3)_2SO$)

170.2, 160.6, 152.9, 139.6, 113.1, 92.2, 85.8, 48.3, 21.2, 14.0; *m/z* (ESI+) calc'd for $C_{10}H_{11}I_2O_3$ $[M+H]^+$ 432.8792, found: 432.8796.

Methyl 4-(ethylamino)-3,5-diiodo-2-methoxybenzoate (66)

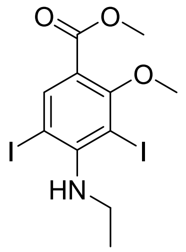

Methyl 4-(ethylamino)-3,5-diiodo-2-methoxybenzoate **66** was synthesized according to general procedure 10; yellow oil (525 mg, 1.14 mmol, 57%); TLC R*f* = 0.35 (5% EtOAc in petroleum ether); FT-IR (ATR) ν_{max}/cm^{-1} 3337, 2947, 1722, 1562, 1421, 1224; ^1H NMR δ_H (400 MHz, $CDCl_3$) 8.31 (s, 1H), 4.05 (br. t, *J* = 7.2 Hz, 1H), 3.89 (s, 3H), 3.86 (s, 3H), 3.39 (quint, *J* = 7.2 Hz, 2H), 1.28 (t, *J* = 7.2 Hz, 3H); ^{13}C NMR δ_C (101 MHz, $CDCl_3$) 163.9, 161.4, 155.6, 143.4, 118.9, 92.2, 82.5, 62.2, 52.5, 44.1, 16.2; *m/z* (ESI+) calc'd for $C_{11}H_{14}I_2NO_3$ $[M+H]^+$ 461.9058, found: 461.9067.

Methyl 3,5-diiodo-2-methoxy-4-(pyrrolidin-1-yl)benzoate (69)

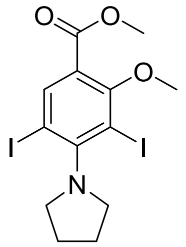

Methyl 3,5-diiodo-2-methoxy-4-(pyrrolidin-1-yl)benzoate **69** was synthesized according to general procedure 10; yellow solid (335 mg, 0.69 mmol, 34%); TLC R*f* = 0.20 (5% EtOAc in petroleum ether); m.p. 63 °C; FT-IR (ATR) ν_{max}/cm^{-1} 2954, 2832, 1730, 1702, 1420, 1233; ^1H NMR δ_H (400 MHz, $CDCl_3$) 8.29 (s, 1H), 3.91 (s, 3H), 3.87 (s, 3H), 3.34 (m, 4H), 2.08 (m, 4H); ^{13}C NMR δ_C (101 MHz, $CDCl_3$) 164.1, 161.1, 155.7, 141.9, 124.0, 103.7, 95.6, 62.4, 52.7, 49.1 , 26.9; *m/z* (ESI+) calc'd for $C_{13}H_{16}I_2NO_3$ $[M+H]^+$ 487.9214, found: 487.9220.

4-(ethylamino)-2-hydroxy-3,5-diiodo-benzoic acid (68) and 4-(ethylamino)-2-hydroxy-3-iodo-benzoic acid (67)

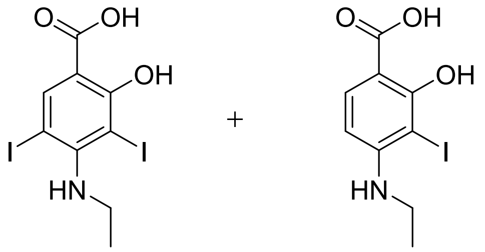

4-(ethylamino)-2-hydroxy-3,5-diiodo-benzoic acid **68** was synthesized according to general procedure 11; isolated as a 10:1 mixture of **68**:**67** (162 mg, 0.38 mmol, 33% **68**); TLC R*f* = 0.15 (15% MeOH in CH_2Cl_2); m.p. 147 °C (decomposition); FT-IR (ATR) ν_{max}/cm^{-1} 3415 br, 1604, 1424; ^{1}H NMR δ_H (400 MHz, $(CD_3)_2SO$) 8.01 (s, 1H), 7.56 (d, *J* = 8.7 Hz, 0.1H), 6.02 (d, *J* = 8.7 Hz, 0.1H), 4.91 (br. s, 0.1H), 3.80 (br. s, 1H), 3.30-3.23 (m, 0.2H), 3.16 (q, *J* = 7.1 Hz, 2H), 1.15 (t, *J* = 7.1 Hz, 3H), 1.12-1.08 (m, 0.3H); ^{13}C NMR δ_C (101 MHz, $(CD_3)_2SO$) 169.2, 165.1, 152.7, 140.1, 115.5, 86.0, 73.9, 43.1, 15.7; *m/z* (ESI+) calc'd for $C_9H_{10}I_2NO_3$ $[M+H]^+$ 433.8745, found: 433.8753.

2-hydroxy-3,5-diiodo-4-(pyrrolidin-1-yl)benzoic acid (70)

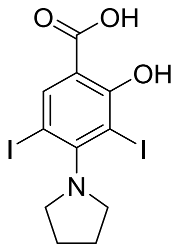

2-hydroxy-3,5-diiodo-4-(pyrrolidin-1-yl)benzoic acid **70** was synthesized according to general procedure 11; (197 mg, 0.43 mmol, 64%); TLC R*f* = 0.25 (15% MeOH in CH_2Cl_2; m.p. 146 °C (decomposition); FT-IR (ATR) ν_{max}/cm^{-1} 3421 br, 2970, 2901, 1654, 1406; ^{1}H NMR δ_H (400 MHz, CD_3OD) 8.31 (s, 1H), 3.36 (m, 4H), 2.10 (m, 4H); ^{13}C NMR δ_C (101 MHz, $(CD_3)_2SO$) 169.8, 162.7, 154.7, 139.5, 114.5, 94.3, 87.6, 48.7 , 26.4; *m/z* (ESI+) calc'd for $C_{11}H_{12}I_2NO_3$ $[M+H]^+$ 459.8901, found: 459.8913.

AMINOBENZOTHIAZOLE SERIES

Methyl 2-amino-4,6-difluorobenzoate (79)

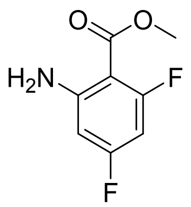

4,6-difluoro-1*H*-indole-2,3-dione **78** (10.2 g, 55.8 mmol) and Cs_2CO_3 (36.4 g, 112 mmol) were stirred in MeOH (110 mL) in a 20 °C water bath. *tert*-butylhydroperoxide (70% aq.) (15.3 mL, 111 mmol) was added over 15 minutes, then the reaction stirred at 20 °C - 30 °C for 2 hours. The reaction was added to sat. aq. $NaHCO_3$ (1 L) and extracted with EtOAc (3 x 1 L). The organic phases were washed with sat. aq. $Na_2S_2O_3$ (500 mL) followed by sat. aq. NaCl (500 mL), then dried ($MgSO_4$) and concentrated *in vacuo* yielding methyl 2-amino-4,6-difluorobenzoate **79** as a yellow solid that was use without further purification (8.42 g, 45.0 mmol, 81%); TLC R*f* = 0.20 (10% EtOAc in heptane); m.p. 81-82 °C; FT-IR (ATR) ν_{max}/cm^{-1} 3457, 3356, 1671, 1591, 1266; ^1H NMR δ_H (400 MHz, $(CD_3)_2SO$) 6.95 (br s, 2H), 6.39 (ddd, J_1 = 12.0 Hz, J_2 = 2.6 Hz, J_3 = 1.5 Hz, 1H), 6.30 (ddd, J_1 = 12.0 Hz, J_2 = 9.4 Hz, J_3 = 2.6 Hz, 1H), 3.79 (s, 3H); ^{13}C NMR δ_C (101 MHz, $(CD_3)_2SO$) 165.7 (d, J = 3.7 Hz), 165.5 (dd, J_1 = 96.5 Hz, J_2 = 17.2 Hz), 163.0 (dd, J_1 = 104.2 Hz, J_2 = 17.6 Hz), 153.6 (dd, J_1 = 15.4 Hz, J_2 = 7.3 Hz), 97.4 (dd, J_1 = 24.2 Hz, J_2 = 2.9 Hz), 97.0 (dd, J_1 = 14.3 Hz, J_2 = 2.6 Hz), 91.4 (t, J = 27.9 Hz), 51.8; ^{19}F NMR δ_F (376 MHz, $(CD_3)_2SO$) -103.1 (br t, J = 12.1 Hz, 1F), -105.2 (q, J = 12.1 Hz, 1F); *m/z* (ESI+) calc'd for $C_8H_8F_2NO_2$ $[M+H]^+$ 188.0518, found: 188.0514.

Methyl 2-amino-4-fluoro-6-methoxybenzoate (80)

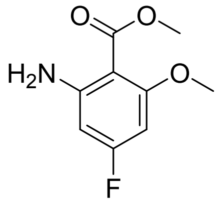

A solution of NaOMe (5.4M, MeOH) (3.80 mL, 20.5 mmol) was added to a solution of methyl 2-amino-4,6-difluorobenzoate **79** (3.49 g, 18.6 mmol) in 1,4-dioxane (37 mL) and heated to 80 °C for 2 hours. The reaction was cooled to room temperature, quenched with AcOH (0.20 mL), then concentrated *in vacuo*. The residue was washed with $NaHCO_3$ (100 mL) and extracted with Et_2O (3 x 100 mL). The organic phases were washed with NaCl (100 mL), dried ($MgSO_4$) concentrated *in vacuo* and purified by flash column chromatography (10% EtOAc in hexane) yielding methyl 2-amino-4-fluoro-6-methoxybenzoate **80** as a yellow solid (2.59 g, 13.0 mmol, 70%); TLC R*f* = 0.15 (10% EtOAc in hexane); m.p. 63-64 °C; FT-IR (ATR) ν_{max}/cm^{-1} 3452, 3346, 1664, 1623, 1585, 1430; ^1H NMR δ_H (400 MHz,

$(CD_3)_2SO$) 6.14-6.09 (m, 3H), 6.07 (dd, J_1 = 11.5 Hz, J_2 = 2.5 Hz), 3.73 (s, 3H), 3.70 (s, 3H); ^{13}C NMR δ_C (101 MHz, $(CD_3)_2SO$) 167.2, 165.0 (d, *J* = 243.6 Hz), 161.3 (d, *J* = 13.9 Hz), 151.4 (d, *J* = 15.4 Hz), 100.3 (d, *J* = 2.2 Hz), 94.0 (d, *J* = 24.9 Hz), 87.5 (d, *J* = 27.1 Hz), 56.0, 51.5; ^{19}F NMR δ_F (376 MHz, $(CD_3)_2SO$) -107.2 (t, *J* = 10.4 Hz, 1F); *m/z* (ESI+) calc'd for $C_9H_{10}F_2NNaO_3$ $[M+Na]^+$ 222.0537, found: 222.0537.

Methyl 2-amino-7-fluoro-5-methoxybenzo[*d*]thiazole-4-carboxylate (81)

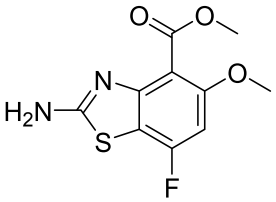

A solution of bromine (2.40 mL, 46.8 mmol) in AcOH (48 mL) was added dropwise over 30 minutes to a 10 °C solution of methyl 2-amino-4-fluoro-6-methoxybenzoate **80** (8.58 g, 43.1 mmol) and KSCN (12.6 g, 129 mmol) in AcOH (96 mL). The reaction was stirred at 10 °C for 30 minutes, then heated to 65 °C for 3 hours. The reaction was cooled, concentrated *in vacuo*, then dissolved in EtOAc (500 mL) and washed with $NaHCO_3$ (2 x 500 mL), then NaCl (500 mL). The organic layer was dried ($MgSO_4$) and concentrated *in vacuo*, then the residue triturated in 0 °C CH_2Cl_2 (30 mL). The precipitate was collected by filtration and washed with 0 °C CH_2Cl_2 (2 x 5 mL) yielding methyl 2-amino-7-fluoro-5-methoxybenzo[*d*]thiazole-4-carboxylate **81** as a yellow solid, which was used without further purification (6.71 g, 26.2 mmol, 61%); TLC R*f* = 0.50 (10% MeOH in CH_2Cl_2); m.p. 210-211 °C; FT-IR (ATR) ν_{max}/cm^{-1} 3101, 2951, 1689, 1585, 1540, 1267; ^{1}H NMR δ_H (400 MHz, $(CD_3)_2SO$) 8.04 (s, 2H), 6.80 (d, *J* = 11.7 Hz, 1H), 3.78 (s, 3H), 3.77 (s, 3H); ^{13}C NMR δ_C (101 MHz, $(CD_3)_2SO$) 168.6, 166.2, 156.4 (d, *J* = 245.0 Hz), 155.9 (d, *J* = 10.3 Hz), 152.9 (d, *J* = 5.9 Hz), 109.4 (d, *J* = 3.7 Hz), 108.4 (d, *J* = 16.9 Hz), 93.1 (d, *J* = 24.2 Hz), 56.6, 52.1; ^{19}F NMR δ_F (376 MHz, $(CD_3)_2SO$) -109.8 (d, *J* = 8.7 Hz, 1F); *m/z* (ESI+) calc'd for $C_{10}H_9FN_2NaO_3S$ $[M+Na]^+$ 279.0210, found: 279.0206.

Methyl 2-(diethylamino)-7-fluoro-5-methoxybenzo[*d*]thiazole-4-carboxylate (82)

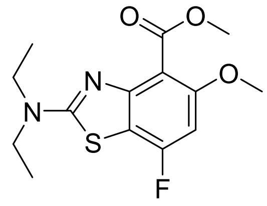

Bromoethane (460 µL, 6.20 mmol) was added to a solution of methyl 2-amino-7-fluoro-5-methoxybenzo[*d*]thiazole-4-carboxylate **81** (762 mg, 2.97 mmol) and Cs_2CO_3 (2.42 g, 7.43 mmol) in DMF (10 mL) and the reaction heated to 60 °C for 4.5 hours. The reaction was cooled to room temperature, then washed with $NaHCO_3$ (100 mL) and extracted with Et_2O (3 x 100 mL). The organic phases were washed with NaCl (100 mL), dried ($MgSO_4$), then concentrated *in vacuo* and the residue purified by flash column chromatography (20% EtOAc in hexane) yielding methyl 2-(diethylamino)-7-fluoro-5-methoxybenzo[*d*]thiazole-4-carboxylate **82** as a yellow solid (525 mg, 1.68 mmol, 57%); TLC R*f* = 0.20 (20% EtOAc in hexane); m.p. 104-105 °C; FT-IR (ATR) ν_{max}/cm^{-1} 2982, 1732, 1540, 1266; ^1H NMR δ_H (400 MHz, $(CD_3)_2SO$) 6.82 (d, *J* = 11.7 Hz, 1H), 3.80 (s, 3H), 3.79 (s, 3H) 3.52 (q, *J* = 7.1 Hz, 4H) 1.20 (t, *J* = 7.1 Hz, 6H); ^{13}C NMR δ_C (101 MHz, $(CD_3)_2SO$) 168.0 (d, *J* = 1.5 Hz), 166.0, 156.5 (d, *J* = 245.0 Hz), 156.2 (d, *J* = 11.0 Hz), 153.1 (d, *J* = 5.9 Hz), 109.5 (d, *J* = 3.7 Hz), 107.9 (d, *J* = 16.9 Hz), 92.9 (d, *J* = 24.2 Hz), 56.6, 51.9, 45.5, 12.5; ^{19}F NMR δ_F (376 MHz, $(CD_3)_2SO$) -109.2 (d, *J* = 10.4 Hz, 1F); *m/z* (ESI+) calc'd for $C_{14}H_{17}FN_2NaO_3S$ $[M+H]^+$ 335.0836, found: 335.0835.

Methyl 2-(diethylamino)-7-fluoro-6-iodo-5-methoxybenzo[*d*]thiazole-4-carboxylate (83)

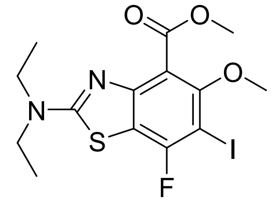

Methyl 2-(diethylamino)-7-fluoro-5-methoxybenzo[*d*]thiazole-4-carboxylate **82** (1.30 g, 4.16 mmol) was dissolved in AcOH (8.5 mL) at 25 °C, then *N*-Iodosuccinimide (970 mg, 4.31 mmol) added. After 1 hour, the reaction was concentrated *in vacuo* and washed with $NaHCO_3$ (50 mL), and extracted with EtOAc (3 x 50 mL). The organic phases were washed with H_2O (2 x 50 mL) followed by sat. aq. NaCl (50 mL), then dried ($MgSO_4$) and concentrated *in vacuo* yielding methyl 2-(diethylamino)-7-fluoro-6-iodo-5-methoxybenzo[*d*]thiazole-4-carboxylate **83** as a yellow solid that was used without further purification (1.75 g, 4.00 mmol, 96%); TLC R*f* = 0.30 (10% EtOAc in hexane); m.p. 79-80 °C; FT-IR (ATR) ν_{max}/cm^{-1} 2975, 2937, 1732, 1532, 1259; ^1H NMR δ_H (400 MHz, $(CD_3)_2SO$) 3.86 (s, 3H), 3.79 (s, 3H), 3.53 (q, *J* = 7.1 Hz, 4H) 1.20 (t, *J* = 7.1 Hz, 6H); ^{13}C NMR δ_C (101 MHz, $(CD_3)_2SO$) 168.0 (d, *J* = 1.5 Hz), 165.4, 156.0 (d, *J* = 5.9 Hz), 155.3 (d, *J* = 243.6 Hz), 153.1 (d, *J* = 5.1 Hz), 114.8 (d, *J* = 3.7 Hz), 112.6 (d, *J* = 19.8 Hz), 69.6 (d, *J* = 24.9 Hz), 62.4, 52.4, 45.7, 12.4; ^{19}F NMR δ_F (376 MHz, $(CD_3)_2SO$) -89.2 (s, 1F); *m/z* (ESI+) calc'd for $C_{14}H_{17}FIN_2O_3S$ $[M+H]^+$ 438.9983, found: 438.9982.

Methyl 2-(diethylamino)-7-(3-chloro-1*H*-pyrrol-1-yl)-6-iodo-5-methoxybenzo[*d*]thiazole-4-carboxylate (86)

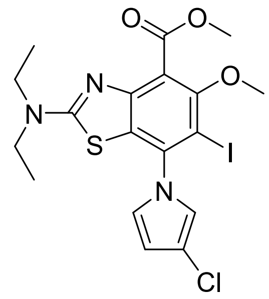

Methyl 2-(diethylamino)-7-fluoro-6-iodo-5-methoxybenzo[*d*]thiazole-4-carboxylate **83** (224 mg, 0.51 mmol), 3-chloro-1-(triisopropylsilyl)pyrrole (166 mg, 0.64 mmol), KF (77.0 mg, 1.33 mmol) and Cs_2CO_3 (432 mg, 1.33 mmol) were dissolved in DMSO (3.5 mL) and heated to 70 °C for 1 hour. The reaction was cooled to room temperature, washed with sat. aq. $NaHCO_3$ (35 mL) and extracted with EtOAc (2 x 35 mL). The organic phases were wash with sat. aq. NaCl (35 mL), dried ($MgSO_4$) and concentrated *in vacuo*, then purified by flash column chromatography yielding methyl 2-(diethylamino)-7-(3-chloro-1*H*-pyrrol-1-yl)-6-iodo-5-methoxybenzo[*d*]thiazole-4-carboxylate **86** as a beige solid (133 mg, 0.26 mmol, 50%); TLC R*f* = 0.25 (10% EtOAc in hexane); m.p. 172-173 °C; FT-IR (ATR) ν_{max}/cm^{-1} 3100, 2970, 2935, 1730, 1535, 1277; ^1H NMR δ_H (400 MHz, $CDCl_3$) 6.75-6.73 (m, 1H), 6.67 (t, *J* = 2.7 Hz, 1H), 6.32 (dd, J_1 = 2.7 Hz, J_2 = 1.7 Hz, 1H), 4.01 (s, 3H), 3.92 (s, 3H), 3.51 (q, *J* = 7.2 Hz, 4H), 1.23 (t, *J* = 7.2 Hz, 6H); ^{13}C NMR δ_C (101 MHz, $CDCl_3$) 168.8, 166.3, 156.3, 152.5, 137.1, 127.9, 121.3, 118.3, 118.2, 113.9, 110.5, 83.7, 63.0, 52.9, 45.9, 12.8; *m/z* (ESI+) calc'd for $C_{18}H_{20}ClIN_3O_3S$ $[M+H]^+$ 519.9953, found: 519.9959.

7-(3-chloro-1*H*-pyrrol-1-yl)-2-(diethylamino)-5-hydroxy-6-iodobenzo[*d*]thiazole-4-carboxylic acid (89)

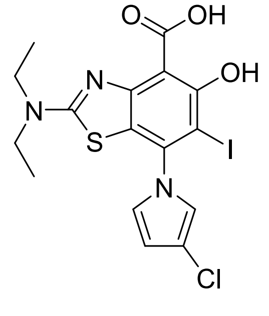

7-(3-chloro-1*H*-pyrrol-1-yl)-2-(diethylamino)-5-hydroxy-6-iodobenzo[*d*]thiazole-4-carboxylic acid **89** was prepared according to general procedure 4; yellow solid (67.6 mg, 0.14 mmol, 73%); TLC R*f* = 0.35

(CH_2Cl_2); m.p. 172-173 °C (decomposition); FT-IR (ATR) ν_{max}/cm^{-1} 2973, 1669, 1521, 1289; ^1H NMR δ_H (400 MHz, $(CD_3)_2SO$) 14.47 (br. s, 1H), 12.7 (br. s, 1H), 7.18 (dd, J_1 = 2.5 Hz, J_2 = 1.7 Hz, 1H), 7.02 (dd, J_1 = 3.1 Hz, J_2 = 2.5 Hz, 1H), 6.39 (dd, J_1 = 3.1 Hz, J_2 = 1.7 Hz, 1H), 3.63-3.53 (m, 4H), 1.23 (t, J = 7.1 Hz, 6H); ^{13}C NMR δ_C (101 MHz, $(CD_3)_2SO$) 170.5, 169.1, 160.8 151.9, 140.1, 121.9, 118.7, 118.3, 112,5, 110.2, 100.7, 79.5, 46.8, 12.1; *m/z* (ESI-) calc'd for $C_{16}H_{14}ClN_3O_3S$ $[M-H]^-$ 489.9495, found: 489.9484.

Methyl 2-(diethylamino)-7-(3-fluoro-1*H*-pyrrol-1-yl)-6-iodo-5-methoxybenzo[*d*]thiazole-4-carboxylate (84)

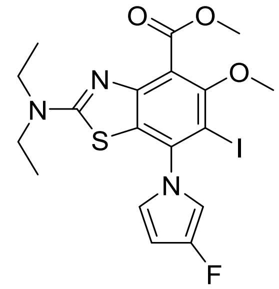

Methyl 2-(diethylamino)-7-fluoro-6-iodo-5-methoxybenzo[*d*]thiazole-4-carboxylate **83** (198 mg, 0.45 mmol), 3-fluoro-1-(triisopropylsilyl)pyrrole **27** (193 mg, 0.80 mmol), KF (76.0 mg, 1.31 mmol) and Cs_2CO_3 (443 mg, 1.36 mmol) were dissolved in DMSO (3 mL) and heated to 50 °C for 4 hours, monitored by LCMS. The reaction was cooled to room temperature, washed with sat. aq. $NaHCO_3$ (30 mL) and extracted with EtOAc (3 x 30 mL). The organic phases were washed with H_2O (30 mL) followed by sat. aq. NaCl (30 mL), then dried ($MgSO_4$) and concentrated *in vacuo*. The residue was purified by flash column chromatography (5% acetone in hexane) yielding methyl 2-(diethylamino)-7-(3-fluoro-1*H*-pyrrol-1-yl)-6-iodo-5-methoxybenzo[*d*]thiazole-4-carboxylate **84** as a beige solid (124 mg, 0.25 mmol, 54%); TLC R*f* = 0.15 (5% acetone in hexane); m.p. 191-192 °C (decomposition); FT-IR (ATR) ν_{max}/cm^{-1} 2936, 1730, 1537, 1257; ^1H NMR δ_H (400 MHz, $CDCl_3$) 6.58-6.55 (m, 1H), 6.51 (dt, J_1 = 4.1 Hz, J_2 = 3.2 Hz, 1H), 6.15 (dd, J_1 = 3.2 Hz, J_2 = 1.8 Hz, 1H), 4.01 (s, 3H), 3.92 (s, 3H), 3.56-3.47 (m, 4H), 1.24 (t, J = 7.2 Hz, 6H); ^{13}C NMR δ_C (101 MHz, $CDCl_3$) 168.9, 166.3, 156.4, 152.9 (d, J = 241.4 Hz), 152.5, 137.5, 128.0, 118.8 (d, J = 5.1 Hz), 118.2, 104.8 (d, J = 28.6 Hz), 99.0 (d, J = 16.9 Hz), 83.9, 63.0, 52.8, 45.9, 12.8; ^{19}F NMR δ_F (376 MHz, $CDCl_3$) -164.3 (t, J = 4.3 Hz, 1F); *m/z* (ESI+) calc'd for $C_{18}H_{20}FIN_3O_3S$ $[M+H]^+$ 504.0249, found: 504.0259.

2-(diethylamino)-7-(3-fluoro-1*H*-pyrrol-1-yl)-5-hydroxy-6-iodobenzo[*d*]thiazole-4-carboxylic acid (87)

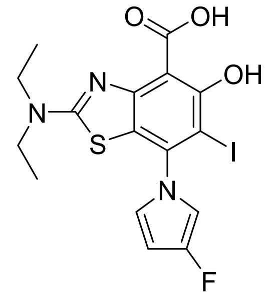

2-(diethylamino)-7-(3-fluoro-1*H*-pyrrol-1-yl)-5-hydroxy-6-iodobenzo[*d*]thiazole-4-carboxylic acid **87** was prepared according to general procedure 4; yellow solid (31.3 mg, 65.9 µmol, 79%); TLC R*f* = 0.30 (30% EtOAc in hexane); m.p. 178-179 °C; FT-IR (ATR) ν_{max}/cm^{-1} 1546, 1242; ^1H NMR δ_H (400 MHz, $(CD_3)_2CO$) 14.42 (br. s, 1H), 12.94 (br. s, 1H), 6.89-6.87 (m, 1H), 6.79 (dd, J_1 = 4.2 Hz, J_2 = 3.3 Hz, 1H), 6.24 (dd, J_1 = 3.3 Hz, J_2 = 1.8 Hz, 1H), 3.71 (br. s, 4H), 1.35 (t, *J* = 7.2 Hz, 6H); ^{13}C NMR δ_C (101 MHz, $(CD_3)_2CO$) 171.9, 171.1, 154.0 (d, *J* = 239.6, Hz), 153.6, 142.0, 121.6, 120.2 (d, *J* = 5.7, Hz), 113.7, 105.8 (d, *J* = 29.3, Hz), 101.8, 100.0 (d, *J* = 17.4, Hz), 78.8, 48.0, 12.7; ^{19}F NMR δ_F (376 MHz, $(CD_3)_2CO$) -165.7 (t, 1F); *m/z* (ESI+) calc'd for $C_{16}H_{16}FIN_3O_3S$ $[M+H]^+$ 475.9936, found: 475.9940.

Methyl 2-(diethylamino)-7-(3,4-difluoro-1*H*-pyrrol-1-yl)-6-iodo-5-methoxybenzo[*d*]thiazole-4-carboxylate (85)

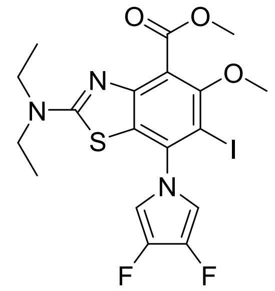

Methyl 2-(diethylamino)-7-fluoro-6-iodo-5-methoxybenzo[*d*]thiazole-4-carboxylate **83** (324 mg, 0.74 mmol), 3,4-difluoro-1-(triisopropylsilyl)pyrrole **30** (290 mg, 1.12 mmol), KF (129 mg, 2.22 mmol) and Cs_2CO_3 (740 mg, 2.27 mmol) were dissolved in DMSO (5 mL) and heated to 50 °C for 2 hours, monitored by LCMS. The reaction was cooled to room temperature, washed with sat. aq. $NaHCO_3$ (50 mL) and extracted with EtOAc (3 x 50 mL). The organic phases were washed with H_2O (50 mL) followed by sat. aq. NaCl (50 mL), then dried ($MgSO_4$) and concentrated *in vacuo*. The residue was purified by flash column chromatography (15% EtOAc in hexane) yielding methyl 2-(diethylamino)-7-(3,4-difluoro-1*H*-pyrrol-1-yl)-6-iodo-5-methoxybenzo[*d*]thiazole-4-carboxylate **85** as a white solid (281 mg, 0.54 mmol, 73%); TLC R*f* = 0.25 (15% EtOAc in hexane); m.p. 219 °C; FT-IR (ATR) ν_{max}/cm^{-1} 3095, 2972, 1729, 1537, 1260; ^1H NMR δ_H (400 MHz, $CDCl_3$) 6.43 (d, *J* = 1.3 Hz, 2H), 4.01 (s, 3H), 3.91 (s, 3H), 3.52 (q, *J* = 7.2 Hz,

4H), 1.24 (t, *J* = 7.2 Hz, 6H); ^{13}C NMR δ_C (101 MHz, $CDCl_3$) 168.8, 166.2, 156.4, 152.6, 139.8 (dd, J_1 = 242.1 Hz, J_2 = 11.7 Hz), 136.9, 128.1, 118.4, 104.1 (m), 83.9, 63.0, 52.9, 46.0, 12.8; ^{19}F NMR δ_F (376 MHz, $CDCl_3$) -177.7 (s, 2F); *m/z* (ESI+) calc'd for $C_{18}H_{19}F_2IN_3O_3S$ $[M+H]^+$ 522.0154, found: 522.0158.

2-(diethylamino)-7-(3,4-difluoro-1*H*-pyrrol-1-yl)-5-hydroxy-6-iodobenzo[*d*]thiazole-4-carboxylic acid (88)

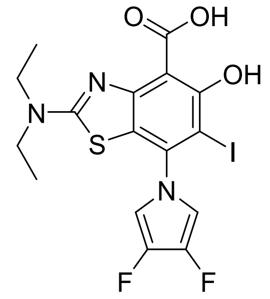

2-(diethylamino)-7-(3,4-difluoro-1*H*-pyrrol-1-yl)-5-hydroxy-6-iodobenzo[*d*]thiazole-4-carboxylic acid **88** was prepared according to general procedure 4; yellow solid (111 mg, 0.23 mmol, 74%); TLC R*f* = 0.35 (CH_2Cl_2); m.p. 216-217 °C; FT-IR (ATR) ν_{max}/cm^{-1} 2978, 1671, 1546; ^{1}H NMR δ_H (400 MHz, $(CD_3)_2CO$) 14.40 (br. s, 1H), 12.86 (s, 1H), 6.90 (d, *J* = 1.3 Hz, 2H), 3.81-3.62 (m, 4H), 1.35 (t, *J* = 7.2 Hz, 6H); ^{13}C NMR δ_C (101 MHz, $(CD_3)_2CO$) 170.8, 170.0, 161.6, 152.6, 140.3, 139.6 (dd, J_1 = 240.3 Hz, J_2 = 11.7 Hz), 119.4, 104.4 (m), 100.8, 78.1, 45.8, 11.7; ^{19}F NMR δ_F (376 MHz, $(CD_3)_2CO$) -180.0 (s, 2F); *m/z* (ESI+) calc'd for $C_{16}H_{15}F_2IN_3O_3S$ $[M+H]^+$ 493.9841, found: 493.9840.

Glide Docking Data

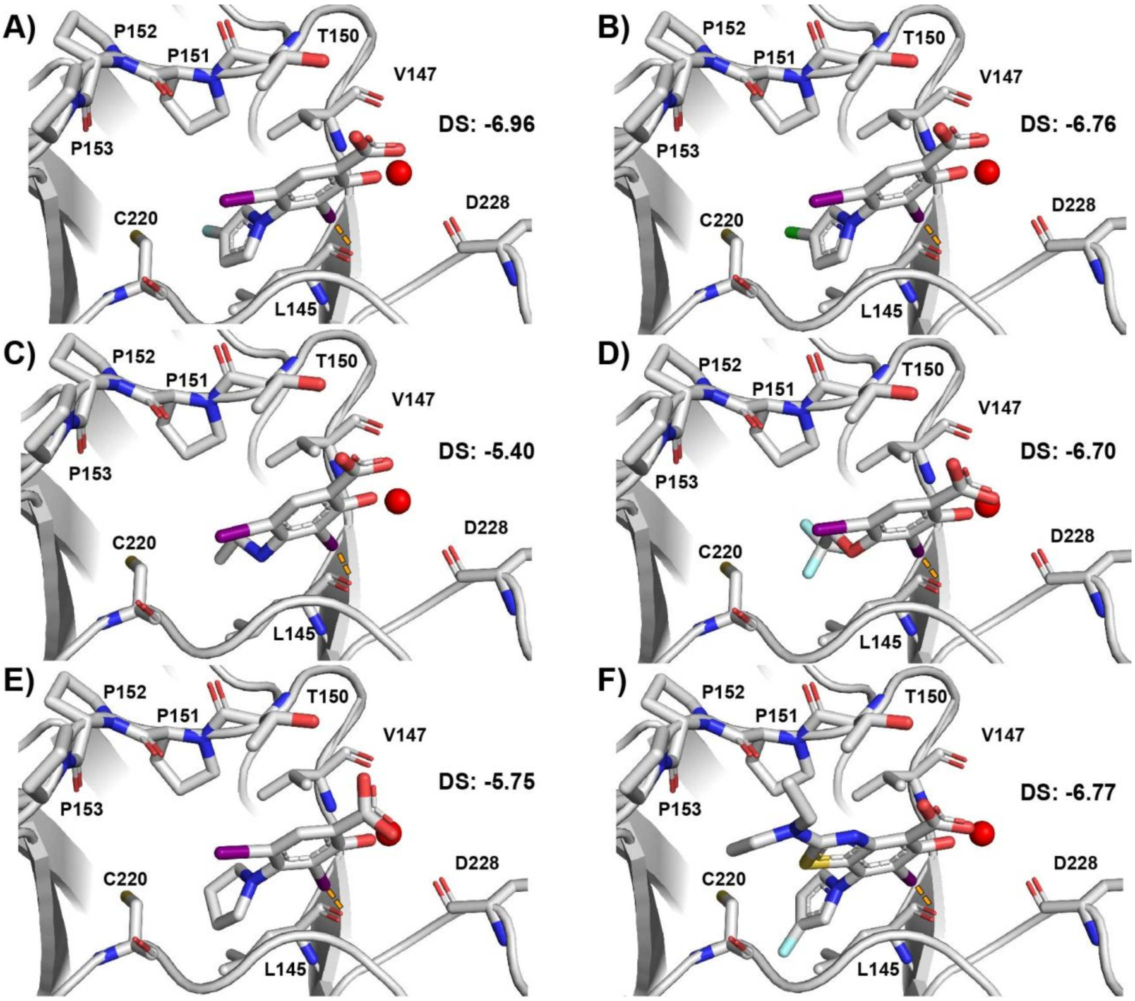

Figure S1. Docking studies. Representative Glide docking poses for iodophenol ligands showing key residues and docking scores (DS). **A**) JC694 (**21**); **B)** JC390 (**20**); **C)** JC563 (**67**); **D)** JC258 (**24**); **E)** JC558 (**70**); **F)** JC744 (**86**).

Biophysical Evaluation Data (DSF, ITC, XRD)

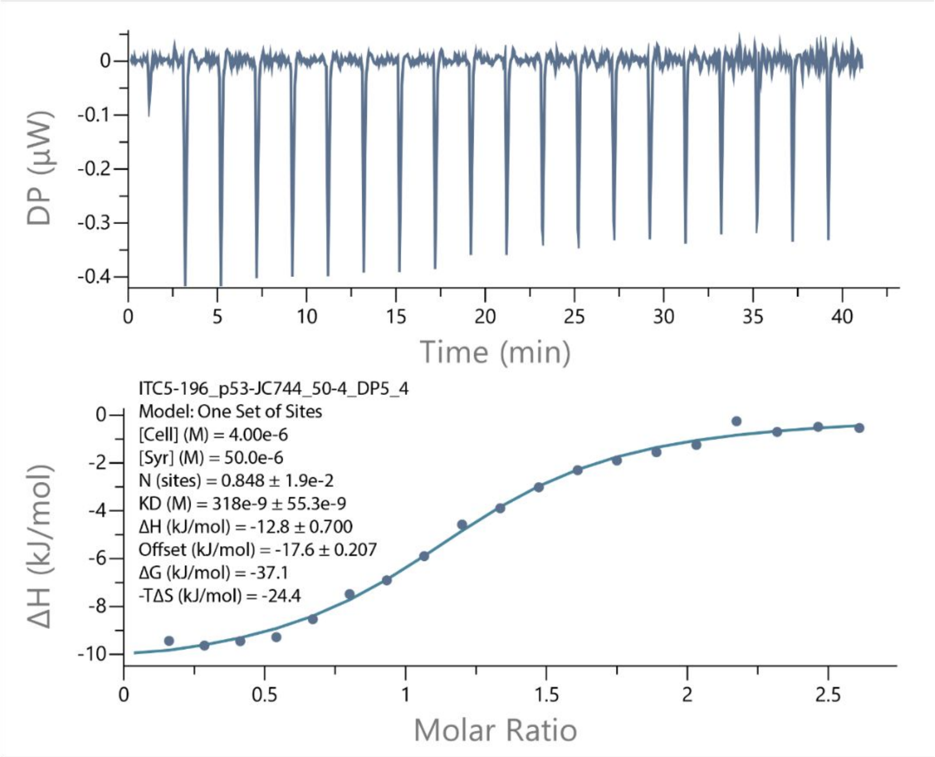

Figure S2. Isothermal titration calorimetry (ITC) titration curve showing binding of JC744 (**86**) to T-p53-Y220C (stabilized DBD, residues 94-312). Reverse titration of protein (50 µM) into JC744 (**86**) (4 µM) at 25 °C.

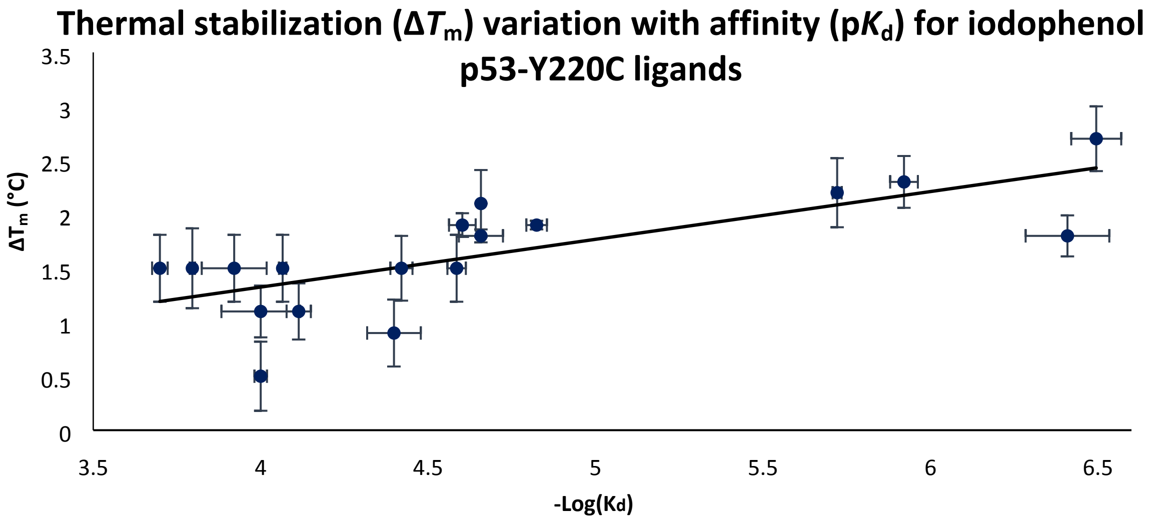

Figure S3. Plotted relationship between thermal stabilization (ΔT_m) of p53-Y220C and affinity (pK_d) of iodophenol ligands with standard deviations.

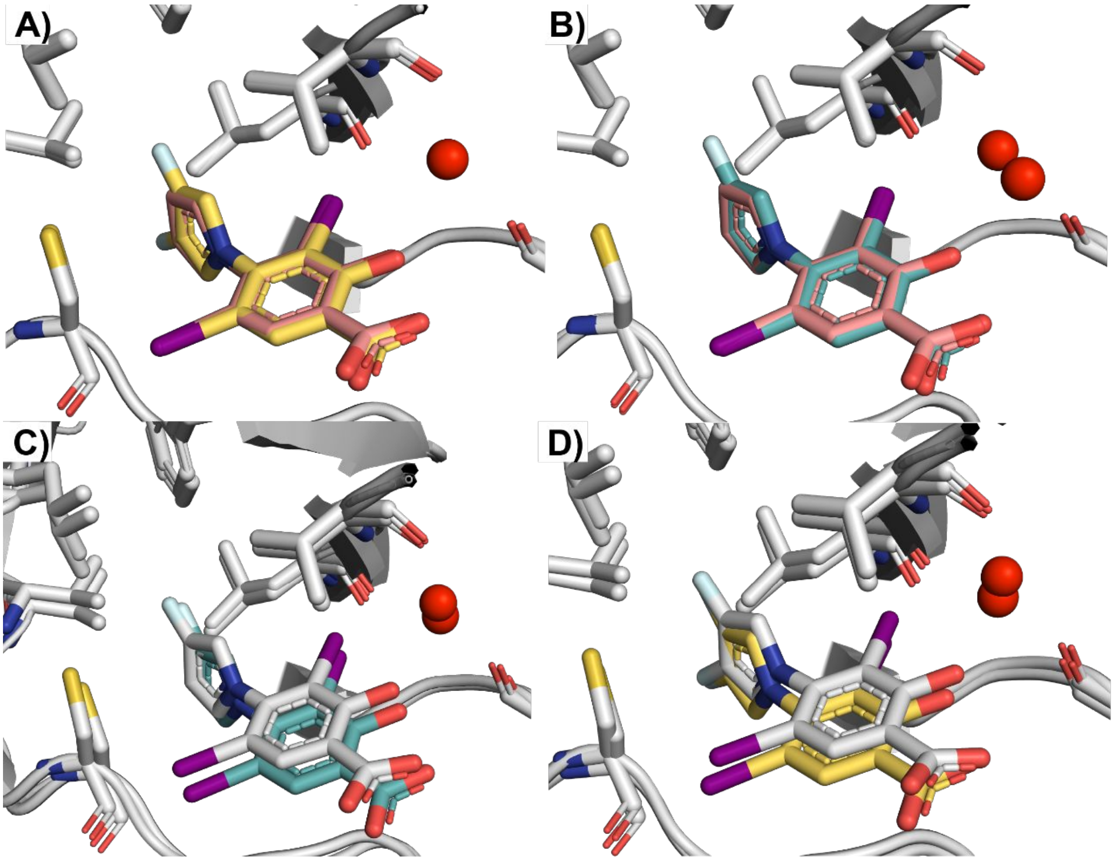

Figure S4. Co-crystal structures of iodophenol ligands (**4**, **21** and **22**) with QM-p53-Y220C. **A**) Overlay of **4** (pink sticks,) and **21** (blue sticks); **B**) Overlay of **4** (pink sticks,) and **22** (yellow sticks); **C**) Overlay of **21** with the docked structure (white sticks); **D**) Overlay of **22** with the docked structure (white sticks).

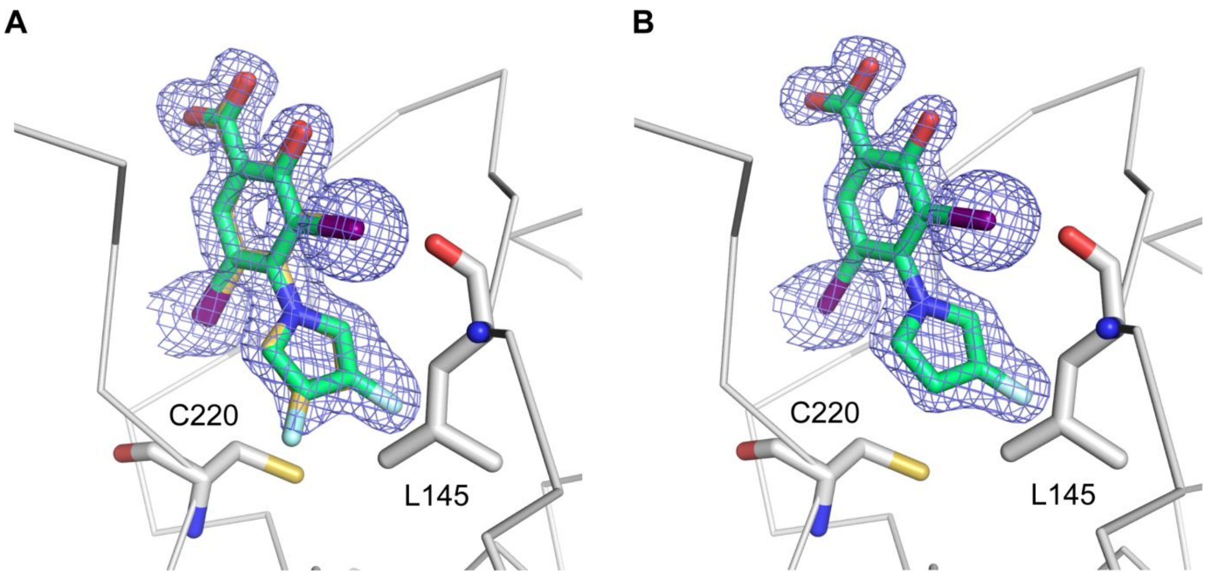

Figure S5. $2F_o$-F_c electron density map for compound **21** (green stick model) bound to the Y220C mutant. The electron density is shown at a contour level of 1.2 σ for both molecules in the asymmetric unit: (**A**) chain A and (**B**) chain B. The electron density in chain A indicated the presence of an alternative conformation at lower occupancy (shown in yellow) where the pyrrole moiety is rotated by 180° (aS conformer).

Table S1. X-ray data collection and refinement statistics of p53-Y220C ligand structures

Complex	JC694 (21)	JC769 (22)
Data Collection		
Space Group	$P2_12_12_1$	$P2_12_12_1$
a (Å)	64.98	65.12
b (Å)	71.06	71.07
c (Å)	105.09	105.32
Molecules per asymmetric unit	2	2
Resolution (Å)[a]	49.5-1.46 (1.48-1.46)	48.01-1.47 (1.50-1.47)
Unique reflections	84,274	83,639
Completeness (%)[a]	99.2 (98.2)	99.9 (99.9)
Multiplicity[a]	5.6 (5.8)	6.0 (6.2)
R_{merge} (%)[a]	6.9 (82.1)	6.1 (109.1)
CC(1/2)[a]	0.999 (0.911)	0.999 (0.842)
Mean $I/\sigma(I)$[a]	12.9 (2.3)	15.4 (1.7)
Refinement		
R_{work}, (%)[b]	15.7	16.4
R_{free}, (%)[b]	19.1	19.3
No. protein atoms[c]	3119	3140
No. zinc atoms	2	2
No. ligand atoms	60	48
No. water molecules	434	440
RMSD bonds (Å)	0.007	0.006
RMSD angles (°)	0.88	0.84
Mean *B* (Å^2)	21.2	23.3
Ramachandran favored (%)[d]	99.8	99.8
Ramachandran outliers (%)[d]	0.0	0.0
PDB code	8A31	8A32

[a]Values in parentheses are for the highest-resolution shell.

[b]R_{work} and R_{free} = $\sum||F_{obs}| - |F_{calc}||/\sum|F_{obs}|$, where R_{free} was calculated with 5 % of the reflections chosen at random and not used in the refinement.

[c]Number includes alternative conformations.

[d]MolProbity statistics[13]

UHPLC traces of representative products assayed *in vitro*

Top 5 Peak Report - UV

Sample ID: JC562 F2 (pyraz)	Submitter: Niama Ezzaidi
Group: Baud, M	Project: RP LC C18 custom
Acquisition Date: 05/04/2022 14:40:50	Instrument: Blue RP UHPLC-MS (B30:1023)
Experiment: BLUE ESIPOSNEG C18 5 min	
Filename: JC562_F2_pyraz_Niama_Ezzaidi__Baud_M__109618.pdf	

Absorbance, NL 1.322E06

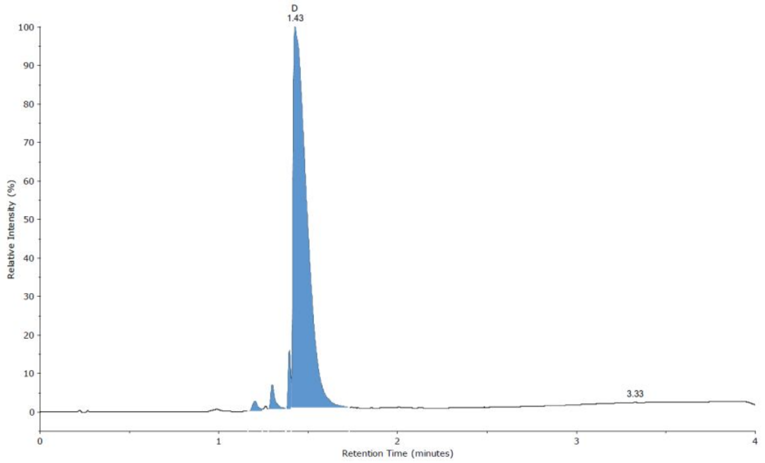

	RT Mins	Height	Height %	Area	Absolute Area %	Relative Area %
A	1.20	31238	2.47	72362	1.01	1.07
B	1.30	82247	6.51	133995	1.87	1.97
C	1.40	150895	11.94	179931	2.51	2.65
D	1.43	1264197	100.00	6786820	94.61	100.00

Figure S5. UHPLC trace of pyrazole derivative **17**.

Top 5 Peak Report - UV

Sample ID: JC413 F2 (Pyr-Me2)	Submitter: Niama Ezzaidi
Group: Baud, M	Project: RP LC C18 custom
Acquisition Date: 05/04/2022 15:12:38	Instrument: Blue RP UHPLC-MS (B30:1023)
Experiment: BLUE ESIPOSNEG C18 5 min	
Filename: JC413_F2_Pyr_Me2_Niama_Ezzaidi__Baud_M__109623.pdf	

Absorbance, NL 1.324E06

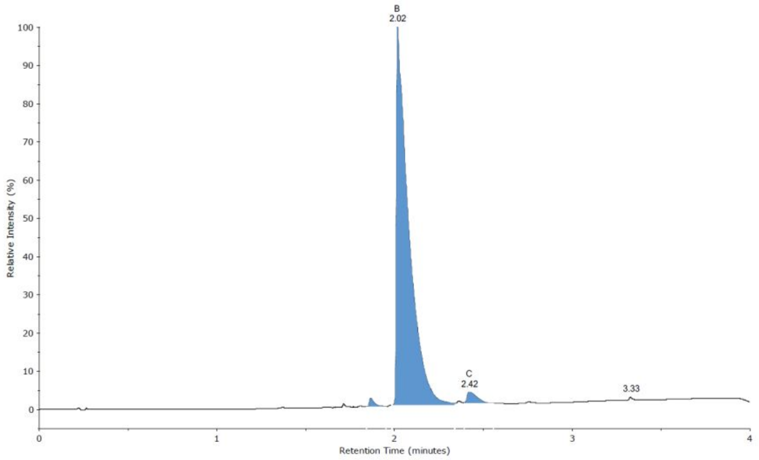

	RT Mins	Height	Height %	Area	Absolute Area %	Relative Area %
A	1.87	28706	2.20	57528	0.90	0.93
B	2.02	1307028	100.00	6195647	96.77	100.00
C	2.42	37665	2.88	148970	2.33	2.40

Figure S6. UHPLC trace of dimethylated pyrrole derivative **45**.

Top 5 Peak Report - UV

Sample ID: JC434P (OMe)
Group: Baud, M
Acquisition Date: 05/04/2022 14:28:13
Experiment: BLUE ESIPOSNEG C18 5 min
Filename: JC434P_OMe_Niama_Ezzaidi__Baud_M__109616.pdf

Submitter: Niama Ezzaidi
Project: RP LC C18 custom
Instrument: Blue RP UHPLC-MS (B30:1023)

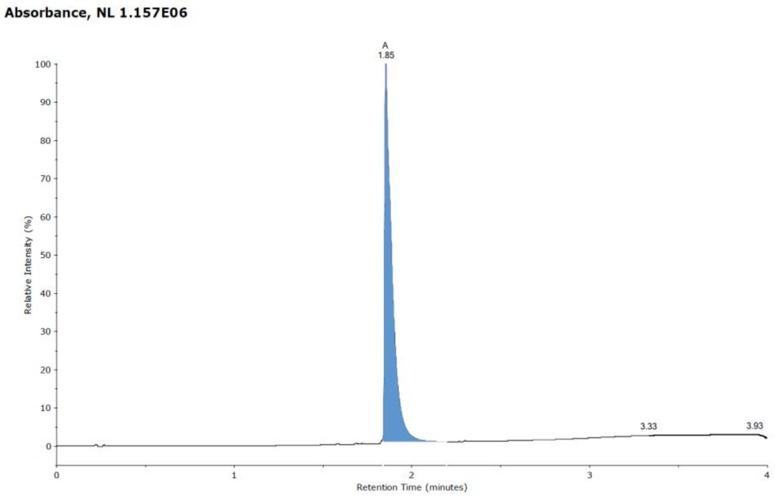

	RT Mins	Height	Height %	Area	Absolute Area %	Relative Area %
A	1.85	1141752	100.00	3179096	100.00	100.00

Figure S7. UHPLC trace of methoxy derivative **62**.

Top 5 Peak Report - UV

Sample ID: JC_925 (F1) (2F)
Group: Baud, M
Acquisition Date: 04/04/2022 17:15:40
Experiment: BLUE ESIPOSNEG C18 5 min
Filename: JC_925_F1_2F_Niama_Ezzaidi__Baud_M__109578.pdf

Submitter: Niama Ezzaidi
Project: RP LC C18 custom
Instrument: Blue RP UHPLC-MS (B30:1023)

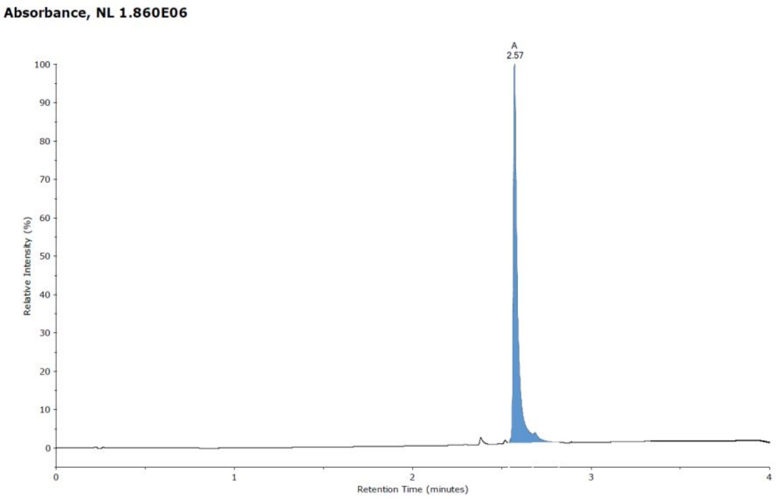

	RT Mins	Height	Height %	Area	Absolute Area %	Relative Area %
A	2.57	1816374	100.00	2787463	96.06	100.00
B	2.69	47745	2.63	114350	3.94	4.10

Figure S8. UHPLC trace of aminobenzothiazole derivative **88**.

References

1. Schrödinger *Maestro*, Schrödinger, LLC: New York, NY, 2019.

2. Schrödinger *LigPrep*, Schrodinger, LLC: New York, NY, 2019.

3. Schrödinger *Glide*, Schrödinger, LLC: New York, NY, 2019.

4. Schrödinger *Receptor Grid Generation*, Schrödinger, LLC: New York, NY, 2019.

5. Schrödinger *Protein Preparation Wizard*, Schrödinger LLC: New York, NY, 2019.

6. Kabsch, W., XDS. *Acta Crystallogr D Biol Crystallogr* **2010,** *66* (Pt 2), 125-132.

7. Evans, P. R.; Murshudov, G. N., How good are my data and what is the resolution? *Acta Crystallogr D Biol Crystallogr* **2013,** *69* (Pt 7), 1204-14.

8. Winn, M. D.; Ballard, C. C.; Cowtan, K. D.; Dodson, E. J.; Emsley, P.; Evans, P. R.; Keegan, R. M.; Krissinel, E. B.; Leslie, A. G.; McCoy, A.; McNicholas, S. J.; Murshudov, G. N.; Pannu, N. S.; Potterton, E. A.; Powell, H. R.; Read, R. J.; Vagin, A.; Wilson, K. S., Overview of the CCP4 suite and current developments. *Acta Crystallogr D Biol Crystallogr* **2011,** *67* (Pt 4), 235-42.

9. Liebschner, D.; Afonine, P. V.; Baker, M. L.; Bunkóczi, G.; Chen, V. B.; Croll, T. I.; Hintze, B.; Hung, L. W.; Jain, S.; McCoy, A. J.; Moriarty, N. W.; Oeffner, R. D.; Poon, B. K.; Prisant, M. G.; Read, R. J.; Richardson, J. S.; Richardson, D. C.; Sammito, M. D.; Sobolev, O. V.; Stockwell, D. H.; Terwilliger, T. C.; Urzhumtsev, A. G.; Videau, L. L.; Williams, C. J.; Adams, P. D., Macromolecular structure determination using X-rays, neutrons and electrons: recent developments in Phenix. *Acta crystallographica. Section D, Structural biology* **2019,** *75* (Pt 10), 861-877.

10. Emsley, P.; Lohkamp, B.; Scott, W. G.; Cowtan, K., Features and development of Coot. *Acta Crystallogr D Biol Crystallogr* **2010,** *66* (Pt 4), 486-501.

11. Joerger, A. C.; Bauer, M. R.; Wilcken, R.; Baud, M. G. J.; Harbrecht, H.; Exner, T. E.; Boeckler, F. M.; Spencer, J.; Fersht, A. R., Exploiting Transient Protein States for the Design of Small-Molecule Stabilizers of Mutant p53. *Structure* **2015,** *23* (12), 2246-2255.

12. Wilcken, R.; Liu, X.; Zimmermann, M. O.; Rutherford, T. J.; Fersht, A. R.; Joerger, A. C.; Boeckler, F. M., Halogen-Enriched Fragment Libraries as Leads for Drug Rescue of Mutant p53. *Journal of the American Chemical Society* **2012,** *134* (15), 6810-6818.

13. Williams, C. J.; Headd, J. J.; Moriarty, N. W.; Prisant, M. G.; Videau, L. L.; Deis, L. N.; Verma, V.; Keedy, D. A.; Hintze, B. J.; Chen, V. B.; Jain, S.; Lewis, S. M.; Arendall, W. B., 3rd; Snoeyink, J.; Adams, P. D.; Lovell, S. C.; Richardson, J. S.; Richardson, D. C., MolProbity: More and better reference data for improved all-atom structure validation. *Protein science : a publication of the Protein Society* **2018,** *27* (1), 293-315.
